# Multifaceted mechanistic exploration of *Geranium wilfordii* Maxim. in asthma treatment: integrating network pharmacology, machine learning, Mendelian randomization and experimental validation

**DOI:** 10.3389/fcell.2026.1761424

**Published:** 2026-03-25

**Authors:** Yingxiang Wu, Kui He, Peng Luo, Tianqian Li, Zhiyan Lu, Ting Fu, Yunchang He, Zhenqi Liu, Fengle Liu, Yang Jin, Yan Wang

**Affiliations:** 1 Department of Pharmacy, Traditional Chinese Medicine Hospital of Dali Bai Autonomous Prefecture, Dali, Yunnan, China; 2 Basic Medical College, Chengdu University of Traditional Chinese Medicine, Chengdu, Sichuan, China; 3 School of Pharmacy, Dali University, Dali, Yunnan, China; 4 Yunnan Key Laboratory of Screening and Research on Anti-pathogenic Plant Resources from Western Yunnan (Cultivation), Dali University, Dali, Yunnan, China

**Keywords:** *Geranium wilfordii* Maxim., machine learning, Mendelian randomization, network pharmacology, single-cell RNA sequencing

## Abstract

**Background:**

Asthma, a chronic inflammatory respiratory disease with significant global health burden, faces limitations in current therapies, necessitating novel therapeutic strategies. The plant *Geranium wilfordii* Maxim. (GWM), a traditional Chinese herbal medicine with diverse pharmacological activities and clinical applications, has been traditionally used in the treatment of rheumatism, numbness, infectious diseases, dermatosis, tumors and other disease by the Bai, Miao, Yi minority people of Southwest China for generations. Earlier research in our lab also demonstrated that GWM exhibits anti-asthmatic activity, but the mechanism of action remains unclear.

**Aim:**

To investigate the anti-asthmatic mechanisms of GWM by identifying compounds and elucidating key molecular targets involved in immune cell regulation through integrated computational and experimental approaches.

**Methods:**

We employed UPLC-QE-Orbitrap-MS to identify active compounds. Network pharmacology and machine learning analyses were conducted to identify key hub genes, followed by validation through Mendelian randomization analysis, molecular docking, and animal models. Immune infiltration and single-cell RNA sequencing analyses using publicly available Gene Expression Omnibus (GEO) datasets, combined with mediation Mendelian randomization (MR), were performed to elucidate the underlying cellular mechanisms.

**Results:**

A total of 43 compounds were identified in GWM. Network pharmacology and machine learning prioritized NOTCH2, HDAC2, and MAPK1 as hub targets, validated using MR and molecular docking. Subsequent *in vivo* experiments validated the regulatory effects of GWM on the expression levels of these hub genes and demonstrated its therapeutic efficacy in asthma. Further analysis showed that GWM regulation of these hub genes may subsequently affect the activity of CD4^+^ T cells and regulatory T cells, potentially contributing to its therapeutic effects against asthma.

**Conclusion:**

This study provides novel evidence for the potential therapeutic activity of GWM in asthma. By targeting key hub genes such as NOTCH2, HDAC2, and MAPK1, GWM may modulate immune cell activity, thereby contributing to its anti-asthmatic effects.

## 1 Introduction

Asthma, a chronic inflammatory respiratory disease characterized by airway hyperresponsiveness (AHR), inflammation, and remodeling (Chen et al., 2023), remains a significant global health challenge, with an estimated 300 million individuals affected worldwide (Tong et al., 2022). A substantial disease burden has been imposed by this condition, marked by impaired quality of life and increased mortality (Walter et al., 2015; Yuan L. et al., 2025). Disproportionately high rates of asthma-related complications and mortality have been observed in low- and middle-income countries, where the majority of cases are concentrated (Morgan et al., 2019; Mortimer et al., 2022). The pathogenesis of asthma is driven by complex interactions between genetic predisposition and environmental triggers such as air pollution and respiratory infections, which collectively promote chronic airway inflammation (Singh et al., 2023). Current therapies, including inhaled corticosteroids (ICS) and long-acting β_2_-agonists (LABA), form the cornerstone of asthma management yet face significant clinical limitations. The consistent adherence to daily routines is frequently hindered by behavioral inconsistencies, challenges in accessing medications, and the complexities associated with inhaler device usage (Anderson et al., 2024). Moreover, prolonged ICS use is associated with systemic adverse effects such as infections and reduced bone mineral density (Shang et al., 2022), while LABA monotherapy may elevate cardiovascular risks (Bian et al., 2020). Additionally, long-term use of these agents may lead to drug tolerance, potentially diminishing therapeutic efficacy (Barnes, 2013). For patients inadequately controlled by high-dose ICS/LABA, the Global Initiative for Asthma (GINA) 2024 guidelines prioritize biologics targeting specific inflammatory pathways, such as omalizumab, as preferred add-on therapies, owing to their ability to reduce exacerbations and improve lung function in phenotype-specific subgroups including eosinophilic or allergic asthma (Rajvanshi et al., 2024). Nevertheless, their widespread application remains constrained by prohibitive costs and hypersensitivity risks (Yang and Castells, 2024). Meanwhile, non-pharmacological strategies such as allergen avoidance are rendered impractical by the ubiquity of environmental triggers like particulate matter and pollen (Thomas et al., 2022). These multifaceted limitations underscore the critical need for cost-effective therapeutic strategies capable of concurrently addressing immune dysregulation and airway repair.

Traditional Chinese Medicine (TCM) has demonstrated therapeutic benefits across various disease types by leveraging its multi-target and holistic regulatory mechanisms, as well as its use of natural ingredients, wide availability, affordability, and minimal side effects, making it particularly valuable for patients who cannot tolerate Western medications (Wang Y. et al., 2025; Ying et al., 2025; Yuan Y. et al., 2025). Recent advances highlight its potential in alleviating asthma through holistic regulation and immunomodulatory effects of herbal compounds (Li et al., 2025b). Among these, *Geranium wilfordii* Maxim. (GWM), first documented in the Ming Dynasty ethnopharmacological text Dian Nan Ben Cao (Lan, 1959) and traditionally used by the Bai, Miao, Yi, Mongolian, and Tibetan minority groups for hundreds of years to treat inflammatory diseases including rheumatism and dermatological disorders (He et al., 2022), has shown promise in preliminary asthma studies. Clinical observations suggest that GWM-containing formulations may alleviate asthma symptoms, positioning it as a potential adjunctive therapy (Xing and Zhang, 2012). The chemical profile of GWM is characterized by abundant polyphenols, tannins, and flavonoids, which collectively contribute to its anti-inflammatory activity (Huang et al., 2015). Among these components, key bioactive constituents identified in GWM have demonstrated independent anti-asthmatic effects in preclinical models, suppressing AHR and eosinophilic infiltration (Cesarone et al., 2019; Chen and Ko, 2021; Xu et al., 2023). Given this, we hypothesize that GWM, through its diverse components working in concert, exerts its anti-asthmatic effects through modulation of inflammatory pathways and immune responses. However, systematic investigations into the multi-component synergy of GWM and its interactions with asthma-related pathophysiological networks remain lacking.

Network pharmacology facilitates a systems-level analysis of interactions between compounds, targets, and diseases, which is particularly valuable for investigating the multi-component nature of TCM (Liu et al., 2025). Machine learning employs computational algorithms to decipher complex patterns within high-dimensional biological datasets, enabling the discovery of pivotal molecular regulators (He et al., 2025; Lalman et al., 2025; Li et al., 2025a). Single-cell RNA sequencing (scRNA-seq) provides cellular-resolution transcriptomic data, uncovering cellular heterogeneity, rare cell states, and gene regulatory networks to reveal disease mechanisms and developmental trajectories (Gul and Zhang, 2025). Mendelian randomization (MR), an analytical approach utilizing progress in expansive genetic association analyses, infers causal links through inherited variants anchored in principles of Mendelian inheritance (Davey Smith et al., 2020; Wang et al., 2023). To investigate the therapeutic role of GWM in asthma and its underlying immune mechanisms, we utilized ultra-high performance liquid chromatography-quadrupole-electrostatic field orbitrap high-resolution mass spectrometry (UPLC-QE-Orbitrap-MS) to identify 43 compounds, followed by network pharmacology and machine learning to highlight three key hub genes (NOTCH2, HDAC2, and MAPK1). Findings were validated through MR analysis, molecular docking, and animal models. Additionally, immune infiltration analysis, scRNA-seq leveraging publicly available GEO datasets and mediation MR provided insights into potential cellular mechanisms. Furthermore, integrating immune infiltration analysis and scRNA-seq based on publicly available GEO datasets, combined with mediation MR, revealed that GWM modulates these hub genes, which in turn regulate the activity of CD4^+^ T cells and regulatory T cells (Tregs), thereby exerting therapeutic effects against asthma.

## 2 Materials and methods

### 2.1 Materials and reagents

GWM was collected from Xizhou Town, Dali City of Yunnan Province located in the southwest of China, which was authenticated by Professor Dequan Zhang (Department of Pharmacognosy, Dali University, China), and the studied specimens were deposited at the authors’ laboratory. Ovalbumin (OVA, Grade V and Grade VI) was purchased from Sigma-Aldrich (St. Louis, MO, United States). Dexamethasone was purchased from Zhejiang Xianju Pharmaceutical Co., Ltd (Shanghai, China). Mouse serum and bronchoalveolar lavage fluid (BALF) enzyme-linked immunosorbent assay (ELISA) kits were provided by Nanjing Jiancheng Bioengineering Institute (Nanjing, China). Wright-Giemsa staining solution was purchased from Beijing Solarbio Science & Technology Co., Ltd. (Beijing, China). The RNA Easy Fast Tissue/Cell Kit was purchased from TIANGEN BIOTECH (BEIJING) Co., Ltd. (Beijing, China). The FastKing RT Kit was provided by TIANGEN BIOTECH (BEIJING) Co., Ltd. (Beijing, China). The SuperReal PreMix Plus (SYBR Green) was obtained from TIANGEN BIOTECH (BEIJING) Co., Ltd. (Beijing, China). Sodium carboxymethyl cellulose (CMC-Na) was supplied by Sinopharm Chemical Reagent Co., Ltd (Shanghai, China). Acetonitrile (HPLC grade), methanol (HPLC grade), and formic acid (LC/MS grade) were all bought from Fisher Scientific (Waltham, United States). Stainless steel beads (3 mm diameter) were purchased from Aladdin Biochemical Technology Co., Ltd (Shanghai, China). Deionized water (18 MΩ) was prepared with a Milli-Q® system (Millipore, United States).

### 2.2 Sample preparation

Approximately 100 mg of GWM sample, ground with liquid nitrogen, was weighed into a 1.5 mL centrifuge tube. Then, 1 mL of 70% methanol-water solution (v/v, containing 4 μg/mL L-2-chlorophenylalanine) was added, followed by vortexing, shaking for 1 min, and adding stainless steel beads. The sample was pre-cooled at −40 °C for 2 min, ground in a mill (60 Hz, 2 min), and subjected to ultrasonic extraction in an ice bath for 60 min. After cooling at −40 °C for 30 min, it was centrifuged (12,000 rpm, 4 °C, 10 min), and the supernatant was filtered through a 0.22 μm organic phase membrane. The filtrate was stored overnight at 4 °C, re-centrifuged under the same conditions, and re-filtered. Finally, 200 μL of the supernatant was transferred to a liquid chromatography-mass spectrometry injection vial for analysis.

### 2.3 Liquid chromatography-mass spectrometry conditions

Sample detection was performed on an ACQUITY UPLC I-Class Plus system coupled with QE-Orbitrap mass spectrometer. Chromatographic separation was achieved on an ACQUITY UPLC HSS T3 column (100 mm × 2.1 mm, 1.8 μm, Waters, Milford, MA, United States) at 45 °C with a flow rate of 0.35 mL/min. The mobile phase consisted of 0.1% formic acid in water (A) and acetonitrile (B), with gradient conditions detailed in Supplementary Table S1. The injection volume was 5 μL. Samples were analyzed using a HESI source in both positive and negative ionization modes under Data-Dependent Acquisition mode on a Full MS/dd-MS2 (Top 8). The spray voltage was set at 3800 V for positive ion mode and −3000 V for negative ion mode. The capillary temperature and auxiliary gas heater temperature were set at 320 °C and 350 °C, respectively. Mass spectra were acquired over an *m/z* range of 100–1,200 Da, with MS and MS/MS resolutions of 70,000 and 17,500, respectively. The collision energies were set to 10, 20, and 40 eV.

### 2.4 Data processing

The raw data underwent comprehensive processing using Progenesis QI v3.0 software, including baseline filtering, peak identification, integration, retention time correction, alignment, and normalization. Compound identification included retention time within 0.2 min of database standards, primary molecular weight error under 5 ppm, and MS/MS spectral matching with standards, referencing the TCM database.

### 2.5 Compound targets prediction for GWM

Compound information (names, structures, SMILES) of GWM components was retrieved from PubChem. Targets were predicted using five platforms: with specific parameters: for BATMAN-TCM, a score cutoff of 20 and an adjusted P-value <0.05 were applied; for PharmMapper, the ‘Maximum Generated Conformations’ was set to 1,000 and the target set was restricted to ‘Human Protein Targets Only (v2010, 2241)’; for DrugBank, SuperPred, and SwissTargetPrediction, all predicted targets were collected without applying specific probability or confidence score thresholds. Human-specific targets were converted to standardized gene symbols via UniProt. Redundant entries were then automatically removed based on the official Gene Symbols to establish a unique set of compound targets. The GWM-ingredient-target network was visualized using Cytoscape (v3.10.1).

### 2.6 Disease targets identification for asthma

#### 2.6.1 Collection of asthma targets in GeneCards database

Asthma-associated genes were identified using the GeneCards database with asthma as the keyword, specifically restricted to *Homo sapiens*.

#### 2.6.2 Identification of differentially expressed genes in asthma

GSE118875 (GPL20301 platform) from the GEO database was first subjected to principal component analysis (PCA) for outlier detection, then analyzed for differentially expressed genes (DEGs) between control and asthma groups using DESeq2 (*P* < 0.05, |log2FC| > 0.5). Volcano plots were generated using the ggplot2 package to display these DEGs, along with heatmaps of the top 50 upregulated DEGs and the top 50 downregulated DEGs ranked by |log2FC| created using the circlize package.

#### 2.6.3 Weighted gene co-expression network analysis

The WGCNA R package was utilized to construct weighted gene co-expression networks for identifying asthma-associated gene modules using the GSE152004 dataset (Platform GPL11154). Genes were filtered by applying a threshold to retain those with variability exceeding the maximum of the first quartile. Samples were clustered to screen and exclude outliers. A soft threshold power was selected to achieve scale-free network topology (scale-free R^2^ > 0.9). The gene co-expression similarity matrix was transformed into a topological overlap matrix, and hierarchical clustering with dynamic tree-cutting (minModuleSize = 40, mergeCutHeight = 0.25) partitioned genes into distinct modules. Correlation matrices between module eigengenes and clinical traits were calculated, while Spearman correlation coefficients between module features and disease phenotypes were computed using the Hmisc R package. Heatmaps generated via the pheatmap R package visually represented module-trait associations and inter-module relationships.

The final high-confidence asthma targets were determined by taking the intersection of the GeneCards results, DEGs, and WGCNA core modules.

### 2.7 Construction of protein-protein interaction network

Compound targets were compiled as the union of BATMAN-TCM, DrugBank, PharmMapper, SuperPred, and SwissTargetPrediction databases, while disease targets were defined by the intersection of GeneCards, asthma-related DEGs, and WGCNA modules. To construct the protein-protein interaction (PPI) network, overlapping targets from the integration of compound and disease targets were imported into the STRING database, selecting *Homo sapiens* as the reference organism and setting the interaction score threshold to 0.4. Isolated nodes were excluded for clarity, and the final network was visualized using the network package (an R package).

### 2.8 Gene ontology and kyoto encyclopedia of genes and genomes enrichment analysis

Gene Ontology (GO) and Kyoto Encyclopedia of Genes and Genomes (KEGG) Pathway analyses were performed using the clusterProfiler package. GO analysis covered biological process, cellular component, and molecular function, with *P* < 0.05 considered significant.

### 2.9 Construction of the drug-compounds-targets-pathways-disease interaction network

The drug-compounds-targets-pathways-disease interactions were visualized in Cytoscape (v3.10.1) to reveal how GWM potentially exerts its therapeutic effects on asthma through key pathways and targets.

### 2.10 Identification of hub genes

The GSE41861 and GSE64913 datasets (GPL570) were retrieved from the GEO, with batch effects removed using the sva package in R and outliers excluded based on PCA plot. Samples were split into 70% training and 30% internal validation sets. External validation datasets (GSE114669, GSE51392, GSE44037; GPL13158) underwent the same preprocessing.

Hub genes were identified through PPI network analysis and machine learning. The top 30 genes by degree centrality in the PPI network were selected as potential hub genes. Five machine learning algorithms—Random Forest (RF), Support Vector Machine-Recursive Feature Elimination (SVM-RFE), Least Absolute Shrinkage and Selection Operator (LASSO), eXtreme Gradient Boosting (XGBoost), and Boruta—were used with overlapping genes from compound and disease targets as input features. Boruta (via the Boruta package) validated feature significance by comparing each variable with randomized shadow features under a significance threshold of p = 0.01 and a maximum of 100 runs. SVM-RFE (e1071 package) recursively removed low-weight features using 10-fold cross-validation, identifying genes based on maximal cross-validation accuracy. LASSO (glmnet package) applied L1 regularization to reduce non-essential coefficients to zero, with the lambda penalty optimized via 10-fold cross-validation to minimize classification error. RF (randomForest package) assessed feature importance using the Mean Decrease Gini Index and built a model with 500 decision trees. XGBoost (xgboost package) was trained using a tree-based booster (max_depth = 6, eta = 0.3, nrounds = 35) and ranked feature importance using Gain values, reflecting each feature’s contribution to performance. SHapley Additive exPlanations (SHAP) interpreted individual gene contributions to the model.

Genes overlapping between the PPI top 30 and results from five machine learning algorithms were identified as core candidates. These underwent validation via differential expression analysis (Wilcoxon rank-sum test, *P* < 0.05) across training, internal, and external datasets. Diagnostic performance was assessed with receiver operating characteristic (ROC) curves (pROC package), and genes with area under the curve (AUC) > 0.7 in all datasets were retained as hub genes.

### 2.11 Nomogram construction and validation

To evaluate the predictive capacity of hub genes for asthma risk (defined as a binary variable: control vs. asthma), a nomogram was developed using the rms package in R with training cohort data. This tool translates multivariate regression outputs into point-based risk estimates for gene expression patterns. Calibration curves assessed agreement between predicted and observed outcomes, while decision curve analysis (DCA) using the rmda package quantified net clinical benefit across risk thresholds. Concurrently, clinical impact curves (CIC) visualized links between risk cutoff values (ranging from 0 to 1) and true positive detection rates, assuming a population size of 1,000.

### 2.12 Mendelian randomization analysis integrating eQTL data

To investigate the causal relationship between gene expression levels and asthma risk, a MR analysis was conducted using expression quantitative trait loci (eQTL) as instrumental variables (IVs). Genetic instruments were derived from genome-wide eQTL data for the identified hub genes were obtained from the eQTLGen consortium (whole blood) via the IEU OpenGWAS database, with variants selected at a significance threshold of *P* < 1 × 10^−5^. To ensure the independence of the IVs, single nucleotide polymorphisms (SNPs) were clumped for independence based on linkage disequilibrium (LD) with r2 < 0.001 within 10 kb. Variants with weak instrument bias (*F* < 10) were excluded. The F-statistic was used to assess the strength of the instrumental variables. For each SNP, the proportion of variance in the exposure explained by the genetic variant (*R* ^*2*^) was first estimated using the formula: *R* ^*2*^ = 2*β* ^2^f (1−f)/(2*β* ^2^f (1−f)+2Nf(1−f)SE^2^), where *β* is the effect size, f isthe effect allele frequency, SE is the standard error, and N is the sample size. The F-statistic was then calculated as: *F* = *R* ^*2*^(N−2)/(1−*R* ^*2*^). Outcome data for asthma susceptibility were obtained from the FinnGen Release 12 cohort, comprising 47,300 cases and 259,839 controls. Exposure and outcome datasets were harmonized to align effect alleles, with incompatible allele pairs excluded. Causal estimates were derived using inverse-variance weighted (IVW) regression as the primary method, complemented by MR-Egger, weighted median, simple mode, and weighted mode methods for robustness. In cases of inconsistency across methods, the IVW estimate was prioritized due to its higher statistical power under balanced pleiotropy assumptions. Sensitivity analyses included Cochran’s *Q* test for heterogeneity, MR-Egger intercept evaluation for horizontal pleiotropy, and leave-one-out validation to identify influential SNPs (Burgess et al., 2017). To validate the directionality of causal associations, the Steiger test was performed for genes showing significant causal relationships with asthma (Hemani et al., 2017). Only genes with a Steiger test *P*-value <0.05 (indicating correct directionality) were retained for downstream analysis. In addition, scatter plots and funnel plots were generated to visually assess the robustness of the findings. A forest plot was also constructed to summarize the causal relationship between individual SNPs and asthma risk. All analyses were conducted using the TwoSampleMR package in R.

### 2.13 Molecular docking verification

The structures of GWM components were obtained from PubChem and energy-minimized using Chem3D software (v20.0). The crystal structures of hub genes were retrieved from the Protein Data Bank. Molecular docking simulations were performed with AutoDock Vina (v1.1.2), and binding affinity values, expressed in kcal/mol, were calculated for each ligand-receptor complex.

### 2.14 Gene Set Enrichment Analysis

To investigate the biological functions and signaling pathways related to key targets, Spearman correlation analysis was conducted between the key targets and all other genes in the training set using the psych R package, ranking genes by Spearman correlation coefficients in descending order. Gene Set Enrichment Analysis (GSEA) was performed with the c2. cp.kegg_medicus.v2024.1. Hs.symbols.gmt dataset from the MSigDB database as the background. The clusterProfiler R package was used, applying thresholds of *P* < 0.05, FDR <0.25, and |NES| > 1. Results, visualized via the enrichplot R package, highlighted the top 5 significantly enriched terms based on |NES| in descending order.

### 2.15 Experimental validation

#### 2.15.1 Preparation of GWM extract

350 g GWM was immersed in 3.5 L 70% methanol (1:10, w/v) overnight and extracted by ultrasonication (40 kHz, 150W) for 1 h. The extracted solution was concentrated under reduced pressure until no methanol remained and freeze-dried. The yield was 15.08% (w/w, relative to dry starting material).

#### 2.15.2 Animals and grouping

Female BALB/c mice (6–8 weeks, 18–22 g, specific pathogen-free) from Spiber Biotechnology Co., Ltd. (Beijing) were housed at the Animal Center of Dali University under a controlled environment (22 °C ± 2 °C, 50% ± 10% humidity, 12-h light/dark cycle) with *ad libitum* access to chow and water. All experimental procedures adhered to the Guideline for Ethical Review of Laboratory Animal Welfare (China, 2018), with a commitment to humane handling and minimizing any discomfort or pain to laboratory animals throughout the study. Experimental protocols followed guidelines approved by the Institutional Animal Care and Use Committee of Dali University (Approval No. 2024-PZ-063). After a 7-day acclimatization period, a total of 36 animals were randomly allocated (Excel RAND function) into six experimental groups (n = 6 per group): healthy control group (Control), ovalbumin-induced asthma model group (OVA), OVA-challenged groups treated with the GWM extract at low-dose (GWM_L: 0.585 g/kg), medium-dose (GWM_M: 1.17 g/kg), or high-dose (GWM_H: 2.34 g/kg), and OVA-challenged group receiving dexamethasone (DEX: 1.0 mg/kg) (Huang M. et al., 2020) as a positive control. To minimize potential confounders, mice were randomized by body weight; treatments were administered in random order; cage positions were rotated weekly; and measurements and sample collections were performed in a fixed sequence.

The required sample size (36 subjects, 6 per group) was estimated using G*Power 3.1.9.7, based on an effect size (f = 0.7) derived from preliminary IL-4 level measurements. The calculation was performed with a significance threshold (α) of 0.05 and a statistical power (1-β) of 0.80. The recommended clinical dose of GWM is 9 g daily, as approved by the 2025 edition of the Chinese Pharmacopoeia. In the current study, the equivalent medium dose (GWM_M) in mice was derived from the 9 g human clinical dose, calculated to be 1.17 g/kg (assuming a 70 kg human) using a body surface area conversion factor of 9.1. Additionally, the low (GWM_L, 0.585 g/kg) and high (GWM_H, 2.34 g/kg) doses were set at 0.5 and 2 times the equivalent clinical dose (GWM_M, 1.17 g/kg), respectively.

#### 2.15.3 Asthma model establishment and treatment

The asthma model involved sensitization and challenge phases, as illustrated in the experimental timeline (Figure 12A) to induce allergic sensitization and trigger airway inflammation. During sensitization (days 0, 7, and 14), antigen-exposed groups (OVA, GWM_L, GWM_M, GWM_H, and DEX) were given three intraperitoneal injections of 500 μg grade VI ovalbumin in 200 μL phosphate-buffered saline (PBS), while control group received the same PBS volume. From day 21 to day 62 (challenge phase), antigen-exposed groups received five weekly intranasal doses of 50 μL 1% grade V OVA in PBS, while control animals were given equivalent PBS volumes.

The lyophilised GWM powder was dissolved in 0.5% CMC-Na to prepare a suspension. Mice in the GWM treatment groups were administered this suspension intragastrically. Thirty minutes before each antigen challenge, GWM_L, GWM_M, GWM_H, and the positive control group (DEX) received their respective treatments via oral gavage, while control and OVA groups received equivalent volumes of 0.5% CMC-Na vehicle. Mice were anesthetized with isoflurane at an induction concentration of 3% and maintained at 1.5% in oxygen via a vaporizer in an induction chamber. Following anesthesia, biological specimens, including bronchoalveolar lavage fluid (BALF), serum, and pulmonary tissues, were collected for subsequent analysis. Animals were then euthanized by cervical dislocation under deep anesthesia to minimize suffering.

#### 2.15.4 Histological examination

Lung tissues were fixed in 4% paraformaldehyde, embedded in paraffin, and sectioned at 5 μm for hematoxylin-eosin (HE) staining. First, the sections were deparaffinized and rehydrated. Sections were then stained with hematoxylin (3–5 min) and eosin (15 s), dehydrated, cleared with xylene, and mounted with neutral balsam. Finally, images were acquired using PANNORAMIC Digital Slide Scanners (Pannoramic MIDI; 3DHISTECH, Budapest, Hungary).

#### 2.15.5 BALF processing and inflammatory cell enumeration

BALF samples were analyzed for inflammatory cell infiltration. Lavage fluid obtained via tracheal cannulation with ice-cold PBS was centrifuged at 2,800 rpm for 5 min at 4 °C to isolate cellular components. Cell pellets were resuspended in PBS for analysis. Total white blood cells (WBC) and subsets (lymphocytes, monocytes, and neutrophils) were quantified using an automated hematology analyzer (BC-2800Vet, Mindray). Eosinophils were enumerated using cytospin preparation and Wright-Giemsa staining, followed by identification through PANNORAMIC Digital Slide Scanners (Pannoramic MIDI; 3DHISTECH) based on their characteristic cytoplasmic granules and bilobed nuclei.

#### 2.15.6 Measurement of cytokines and immunoglobulins

IL-4 in serum and IL-5, IL-13, and IgE in BALF supernatant were quantified using commercial ELISA kits following the standard protocol. Briefly, pre-coated 96-well plates were incubated with diluted samples and standards along with biotinylated antigen at 37 °C for 30 min. After washing, avidin-conjugated horseradish peroxidase was added for a 30-min incubation at 37 °C. The chromogenic reaction was developed by sequentially adding chromogen solutions A and B and incubating at 37 °C for 10 min. The reaction was stopped with 2 M sulfuric acid, and the absorbance was measured at 450 nm using a microplate reader (Thermo Fisher Scientific, Waltham, MA, United States). All assays were performed in duplicate, and sample dilutions were adjusted to ensure that all measurements fell within the linear range of the standard curves.

#### 2.15.7 RNA extraction and gene expression analysis

To validate the expression of hub genes involved in asthma, total RNA was extracted from lung tissues of three mice per group using the RNA Easy Fast Tissue/Cell Kit. RNA concentration and purity were assessed using a Nucleic Acid Analyzer (Luye Bio-Tech Co., Ltd., Yantai, Shandong, China); only samples meeting the standard quality criteria (A_260_/A_280_ of 1.8–2.2 and A_260_/A_230_ of 2.0–2.5) were used for subsequent analysis. cDNA was synthesized using the FastKing RT Kit (With gDNase). Quantitative PCR was performed using SuperReal PreMix Plus (SYBR Green) on the Pangaea Rapid Fluorescence PCR System (Aperbio, Suzhou, China), with three technical replicates per sample. Relative gene expression levels were normalized to β-actin as the reference gene. Primer sequences are listed in Supplementary Table S2. PCR was performed in a total volume of 20 μL with initial denaturation at 95 °C for 15 min, followed by 40 cycles of 95 °C for 10 s and 60 °C for 32 s. A melting curve analysis was conducted from 60 °C to 95 °C with increments of 0.5 °C every 5 s.

### 2.16 Single-cell RNA sequencing data analysis

scRNA-seq data (GSE130148) from the GEO database was processed using Seurat. Quality control excluded cells with RNA counts outside 200–30000, fewer than 200 or more than 7,000 genes, mitochondrial gene expression >20%, or red blood cell gene expression >3%. Data were normalized using the LogNormalize method (scale factor = 10,000) and subjected to PCA on highly variable genes, with JackStraw Plot and Elbow Plot determining the optimal principal component. Data were then clustered (FindClusters, resolution = 0.8) and visualized by Uniform Manifold Approximation and Projection (UMAP). Cell annotation utilized marker genes and SingleR. Cell trajectories were reconstructed with Monocle 2. Cells were categorized into positive and negative groups based on a binary expression threshold (>0) of the hub genes. A normalized UMI matrix from Seurat objects built the CellDataSet, with size factors and gene dispersions calculated. DDRTree-based dimensionality reduction inferred developmental trajectories. Intercellular communication was analyzed using CellChat with the CellChatDB.human database. Overexpressed ligands or receptors in cell groups were quantified, with outgoing and incoming signal intensities compared between subtypes. Interaction weights, pathway activities, and ligand-receptor contributions were visualized through heatmaps, circle plots, hierarchy charts, and bubble diagrams.

### 2.17 Immune cell infiltration analysis

The relative abundance of 22 immune cell types were quantified using the R package CIBERSORT. Histograms showing immune cell distributions were generated with ggplot2, excluding samples with *P* > 0.05. Differential infiltration levels between groups were compared with the Wilcoxon rank-sum test, visualized as boxplots. Spearman correlation analysis, performed with the psych package, assessed associations among differentially infiltrated immune cells (*P* < 0.05, |cor| > 0.3), with results displayed as correlation heatmaps. Associations between hub genes and differentially infiltrated immune cells were also evaluated using Spearman correlation and visualized via lollipop plots.

### 2.18 Mediation analysis

Mediation analysis employed two-step MR to assess whether 731 GWAS Catalog immune cells (Orrù et al., 2020) mediated causal pathways between hub genes and asthma. The total causal effect of hub genes on asthma outcomes was estimated via MR, producing total effect estimates, consistent with prior MR analyses using eQTL data. Reverse MR was then conducted to test if asthma causally affects hub genes. MR analyses evaluated the effects of hub genes on immune cells (β1) and immune cells on asthma (β2). The selection of instrumental variables for the 731 immune cell traits, including P-value thresholds and local LD clumping, followed the same rigorous criteria as described in the primary Mendelian randomization analysis. The indirect effect was obtained by multiplying β1 and β2, while the direct effect was calculated by subtracting the indirect effect from the total effect. Mediation percentage was calculated as the ratio of the indirect to total effect, with 95% confidence intervals estimated by the delta method. Sensitivity analyses assessed robustness, including heterogeneity testing and horizontal pleiotropy evaluation. For cases with heterogeneity, a random-effects IVW model, less sensitive to weaker SNP-exposure associations, was used to ensure result stability (Feng et al., 2024).

### 2.19 Statistical analysis

All data analyses were conducted using R software (v 4.3.1) and SPSS (v 26.0). Continuous variables were assessed for normality using the Shapiro-Wilk test. For those meeting the normality assumption, results were presented as the mean ± standard deviation (SD), and one-way ANOVA was used to analyze group differences. Homogeneity of variances was checked using the Levene test. If homogeneity was met (*P* ≥ 0.05), the least significant difference test was applied for *post hoc* comparisons; if violated (*P* < 0.05), Dunnett’s T3 method was used. If the normality assumption was not met, data were described using the median and interquartile range, and group differences were analyzed using the Kruskal-Wallis test. Statistical significance was set at *P* < 0.05 (two-sided). Sample outliers were detected using Principal Component Analysis (PCA) and interquartile range (IQR)-based thresholds, with PCA identifying samples deviating from the main data structure and IQR identifying samples exceeding 1.5 × IQR from the median.

## 3 Results

### 3.1 Compound identification and profiling

**FIGURE 1:**
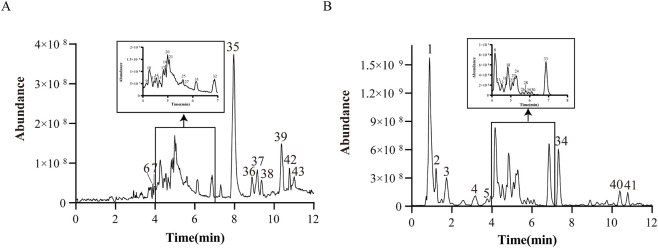
Base peak chromatograms of GWM obtained by UPLC-QE-Orbitrap-MS analysis. **(A)** Base peak chromatogram in positive ionization mode. **(B)** Base peak chromatogram in negative ionization mode.

The base peak chromatograms in both positive and negative ion modes are presented in Figure 1 using the optimal conditions. A total of 43 compounds were identified in GWM (22 in positive ion mode and 21 in negative ion mode) through comparison with reference standards, compound databases, and published literature (Yang et al., 2011; Li, 2020; Zeng et al., 2023; Chen and Lan, 2025; Quan et al., 2025). These compounds include 20 flavonoids, 9 phenylpropanoids, 6 terpenoids, 4 phenols, 2 sugars and glycosides, 1 organic acid, and 1 other compound. Detailed information on these compounds is provided in Table 1.

**TABLE 1:** Characterization of chemical constituents of *Geranium wilfordii* Maxim. Extract by UPLC-QE-Orbitrap-MS.

No.	t_R_ (min)	Identification	Measured (*m/z*, mode)	ppm	Elemental composition	Molecular weight (Da)	MS/MS (*m/z*)	Source
1	0.96	3-Galloylquinic acid	343.0671 (−)	1.75	C_14_H_16_O_10_	344.0743	343.0667,191.0563,169.0143,127.0399,111.0450	a
2	1.21	1-beta-D-Arabinofuranosyluracil	243.0623 (−)	2.47	C_9_H_12_N_2_O_6_	244.0695	200.0558,183.0409,153.0307,140.0356, 110.0249, 82.0300	b
3	1.74	Gallic acid	169.0144 (−)	4.14	C_7_H_6_O_5_	170.0215	169.0144,151.0031, 125.0246, 97.0279,69.0347	a
4	3.19	Protocatechuic acid	153.0194 (−)	3.92	C_7_H_6_O_4_	154.0266	153.0181,136.0156, 109.0297, 108.0202,91.0176	c
5	3.79	4- O-beta-Glucopyranosyl-cis-coumaric acid	325.0923 (−)	2.46	C_15_H_18_O_8_	326.1002	371.0987 [M-H + HCOO]^-^, 325.0923 [M-H]^-^,209.0093,163.0402,119.0504	b
6	3.92	Procyanidin B1	579.1490 (+)	−2.24	C_30_H_26_O_12_	578.1424	427.1028, 409.0919, 301.0708, 289.0704, 271.0602	d
7	3.98	Brevifolincarboxylic acid	293.0287 (+)	−3.41	C_13_H_8_O_8_	292.0219	275.0184,265.0340,247.0234, 219.0285, 191.0336	e
8	4.16	Catechin	289.0716 (−)	1.38	C_15_H_14_O_6_	290.0790	289.0716,245.0820,205.0501, 151.0404, 109.0296	d
9	4.16	Epicatechin	291.0859 (+)	−3.44	C_15_H_14_O_6_	290.0790	291.0861,207.065, 165.0545, 139.0389,123.0442	d
10	4.28	Myricetin 3-beta-D-glucopyranoside	481.0979 (+)	−0.62	C_21_H_20_O_13_	480.0904	319.0447, 301.0347,290.0420, 273.0397,85.0290	d
11	4.31	Roseoside	385.1874 (−)	3.12	C_19_H_30_O_8_	386.1941	431.1925 [M-H + HCOO]^-^, 385.1874 [M-H]^-^,223.1341, 205.1240, 179.0566,153.0921	f
12	4.46	Quercetin 3-sambubioside	597.1471 (+)	−0.67	C_26_H_28_O_16_	596.1377	597.1471,465.1026,345.0591, 303.0498, 285.0392	d
13	4.51	Myricetin 3-O-rutinoside	625.1414 (−)	1.44	C_27_H_30_O_17_	626.1483	625.1410,316.0224,299.0182, 287.0203, 271.0247	d
14	4.54	Rutin	611.1608 (+)	−0.65	C_27_H_30_O_16_	610.1534	611.1594,465.1019, 303.0497, 85.0289, 71.0498	d
15	4.68	Hyperoside	465.1030 (+)	−0.65	C_21_H_20_O_12_	464.0955	465.1044,303.0497,303.0127, 216.0065, 85.0289	d
16	4.72	3,4,8,9,10-Pentahydroxy Urolithin	275.0197 (−)	1.82	C_13_H_8_O_7_	276.0270	275.0195,258.0168,247.0249 229.0142, 203.0347	a
17	4.82	Kaempferitrin	579.1710 (+)	−0.69	C_27_H_30_O_14_	578.1636	433.1127,397.0904, 287.0549, 129.0547, 85.0290	d
18	4.85	7-[(beta-D-Glucopyranosyl)oxy]-3',4',5,8-tetrahydroxyflavone	463.0886 (−)	1.94	C_21_H_20_O_12_	464.0955	463.0881,300.0275, 271.0251,283.0252,257.0449	d
19	4.93	Kaempferol-3-O-glucorhamnoside	595.1658 (+)	−0.84	C_27_H_30_O_15_	594.1585	449.1115, 433.113, 287.0549,329.0669, 85.0290	d
20	5.02	Isovanillin	153.0546 (+)	−3.27	C_8_H_8_O_3_	152.0473	153.0546,125.0598, 109.0652,111.0443,93.0339	a
21	5.08	Vomifoliol	207.1378 (+)	−3.38	C_13_H_20_O_3_	224.1412	207.1378 [M + H-H_2_O]^+^,189.1273, 161.1324,123.0826,95.0860	g
22	5.09	Sophorabioside	577.1567 (−)	1.73	C_27_H_30_O_14_	578.1636	623.1625 [M-H + HCOO]^-^ 577.1567 [M-H]^-^, 431.0978, 285.0401, 169.0142	d
23	5.23	Biorobin	593.1515 (−)	1.52	C_27_H_30_O_15_	594.1585	593.1508,327.0516,284.0327, 255.0301, 227.0354	d
24	5.30	Aviculin	505.2075 (−)	0.20	C_26_H_34_O_10_	506.2152	551.2141 [M-H + HCOO]^-^ 505.2075 [M-H]^-^,359.1500, 344.1262, 59.0140	e
25	5.59	Aurantio-obtusin beta-D-glucoside	493.1343 (+)	−0.61	C_23_H_24_O_12_	492.1268	493.1329,373.0899,331.0809, 316.0577, 298.0472	e
26	5.62	Hypoletin-7-O-beta-D-xylopyranoside	433.0776 (−)	1.15	C_20_H_18_O_11_	434.0849	867.1632 [2M-H]^-^,433.0776 [M-H]-,300.0275 271.0248,166.9989	d
27	5.69	Kaempferol-7-O-rhamnoside	433.1112 (+)	−5.31	C_21_H_20_O_10_	432.1056	433.1112,287.0548, 153.0181,121.0295,85.0287	d
28	5.79	Gallocatechin	287.0564 (−)	2.79	C_15_H_14_O_7_	306.0740	287.0560 [M-H-H_2_O]^-^, 259.0611, 201.0567,151.0038,125.0239	d
29	5.95	Methyl p-coumarate	177.0559 (−)	3.95	C_10_H_10_O_3_	178.0630	177.0562,162.0327,145.0295,123.0454,117.0348	e
30	6.11	Apigenin	269.0454 (−)	2.60	C_15_H_10_O_5_	270.0528	269.0454,241.0501,225.0570, 151.0042,117.0349	d
31	6.15	Syringaresinol	401.1597 (+)	−0.75	C_22_H_26_O_8_	418.1628	401.1597 [M + H-H_2_O]^-^, 383.1486, 371.1496, 330.1096,217.0854	e
32	6.89	Medioresil	371.1480 (+)	−4.04	C_21_H_24_O_7_	388.1522	371.1470 [M + H-H_2_O]^+^, 353.1386, 341.1377, 339.1227,217.0858	e
33	6.9	Kaempferol	285.0405 (−)	2.10	C_15_H_10_O_6_	286.0477	285.0406,257.0525,211.0442,185.0635,151.0031	d
34	7.31	Isorhamnetin	315.0512 (−)	2.22	C_16_H_12_O_7_	316.0583	315.0513,300.0276, 271.0261, 164.0113, 151.0031	d
35	8.00	Dihydroactinidiolide	181.1219 (+)	−5.52	C_11_H_16_O_2_	180.1150	181.1219,163.1116,153.1272, 137.0964, 125.0966	f
36	8.87	Magnolin	417.1919 (+)	−3.01	C_23_H_28_O_7_	416.1835	399.1796 [M + H-H_2_O]^+^, 381.1695, 369.1691,368.1599,151.0753	e
37	9.07	Yangambin	429.1911 (+)	−0.47	C_24_H_30_O_8_	446.1941	429.1911 [M + H-H2O]^+^,411.1794 399.1796,384.1553,343.1174	e
38	9.39	Kobusin	353.1381 (+)	−2.27	C_21_H_22_O_6_	370.1416	353.1381 [M + H-H_2_O]^+^,308.1042,283.0964, 231.1016,201.0908, 135.0440	e
39	10.36	Momordicine I	455.3502 (+)	−5.05	C_30_H_48_O_4_	472.3553	455.3102 [M + H-H_2_O]^+^, 437.3440, 409.3464, 313.2734,271.2052, 233.1891	f
40	10.39	Pinobanksin 3-acetate	313.0720 (−)	2.56	C_17_H_14_O_6_	314.0790	313.0720,271.0619, 253.0505, 197.0613,147.0086	d
41	10.78	Hederagenin	471.3492 (−)	3.82	C_30_H_48_O_4_	472.3553	517.3527 [M-H + HCOO]^-^ 471.3492 [M-H]^-^, 152.9955, 141.092, 78.9591	f
42	10.79	Alismol	203.1792 (+)	−3.94	C_15_H_24_O	220.1827	203.1792 [M + H-H_2_O]^+^, 175.1479, 160.1242,105.0702, 60.0452	f
43	11.03	Caryophyllene oxide	221.1898 (+)	−3.16	C_15_H_24_O	220.1827	176.1068, 147.1168, 165.1274, 5.0860, 81.0705	f

a: Phenols; b: Sugars and glycosides; c: Organic acids and their derivatives; d: Flavonoids; e: Phenylpropanoids; f: Terpenes; g: Others.

#### 3.1.1 Identification of phenolic compounds

**FIGURE 2:**
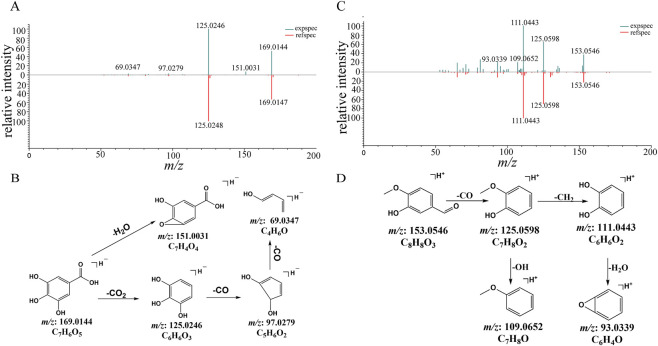
The MS/MS spectra and proposed fragmentation pathways of phenols identified in GWM. **(A)** The MS/MS spectrum of compound 3 (gallic acid). **(B)** The proposed fragmentation pathways of gallic acid. **(C)** The MS/MS spectrum of compound 20 (isovanillin). **(D)** The proposed fragmentation pathways of isovanillin.

Compound 3 displayed a [M-H]^-^ ion at *m/z* 169.0144, which showed successive losses of 44 Da, 28 Da, and 28 Da, and generated *m/z* 125.0246, *m/z* 97.0279, and *m/z* 69.0347 ions, corresponding to the [M-H-CO_2_]^−^, [M-H-CO_2_-CO]^−^, and [M-H-CO_2_-2CO]^−^ fragment ions (Figure 2A). Compound 3 was identified as gallic acid and confirmed by comparing structural information of MS/MS spectrum with reference standard. The proposed fragmentation pathway is illustrated in Figure 2B.

In the MS spectrum of compound 20, a [M + H]^+^ ion at *m/z* 153.0546 was observed, and MS/MS fragment ions appeared at *m/z* 125.0598, 111.0443, 109.0652 and 93.0339, corresponding to [M + H− CO]^+^, [M + H−CO−CH_2_]^+^, [M + H−CO−OH]^+^, and [M + H−CO−CH_2_−H_2_O]^+^ ions (Figures 2C,D). Compound 20 was definitively identified by comparison with the reference standard. Using a similar approach, other phenolic compounds (compounds 1 and 16) were characterized.

#### 3.1.2 Identification of flavonoid compounds

**FIGURE 3:**
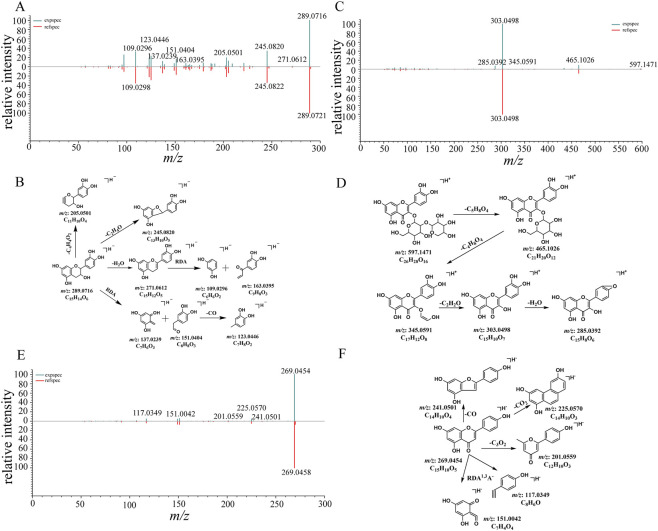
The MS/MS spectra and proposed fragmentation pathways of flavonoids identified in GWM. **(A)** The MS/MS spectrum of compound 8 (catechin). **(B)** The proposed fragmentation pathways of catechin. **(C)** The MS/MS spectrum of compound 12 (quercetin 3-sambubioside). **(D)** The proposed fragmentation pathways of quercetin 3-sambubioside. **(E)** The MS/MS spectrum of compound 30 (apigenin). **(F)** The proposed fragmentation pathways of apigenin.

Compound 8 demonstrated a deprotonated ion with mass accuracy at *m/z* 289.0716 (calculated for [M-H]^-^, 289.0721), which could lose one molecule of H_2_O to form the fragment with *m/z* 271.0612, and then underwent Retro Diels-Alder (RDA) reaction to produce ions at *m/z* 109.0296, and *m/z* 163.0395 (Figure 3A). The RDA fragment ion at *m/z* 151.0404 from the precursor ion [M-H]^-^ indicated that two hydroxyl groups were attached to the B-ring. The ion at *m/z* 151.0404 could further lose one molecule of CO to produce ion at *m/z* 123.0446. Compound 8 was unambiguously attributed to catechin by comparison with the reference standard. The proposed fragmentation pathway is presented in Figure 3B.

Compound 12 showed a [M + H]^+^ at *m/z* 597.1471 in positive-ion mode. The ions at *m/z* 303.0498 given by MS/MS spectrum suggesting the loss of sambubioside (C_11_H_18_O_9_) from the precursor ion [M + H]^+^, and then loss of H_2_O gave rise to the fragment at *m/z* 285.0392 (Figures 3C,D). Compound 12 was assigned as quercetin 3-sambubioside by comparison with the reference standard.

The MS/MS fragments of compound 30 ([M-H]^-^ at *m/z* 269.0454) produced a [M-H-C_8_H_6_O]^-^ ion with a loss of 117.0349 Da, which was consistent with the representative fragmentation pathway of flavones, and suggested the presence of a hydroxyl group in the B-ring (Figures 3E,F). Compound 30 was unambiguously identified as apigenin by comparison with the reference standard.

#### 3.1.3 Identification of sugar and glycoside compounds

**FIGURE 4:**
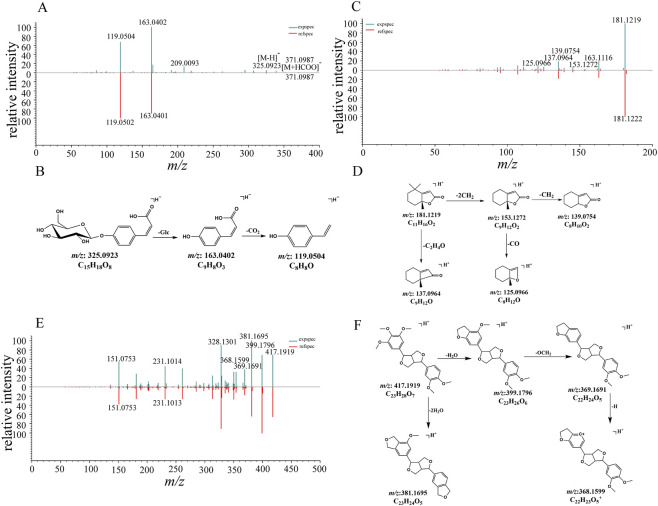
The MS/MS spectra and proposed fragmentation pathways of sugars and glycosides, terpenes, and phenylpropanoids identified in GWM. **(A)** The MS/MS spectrum of compound 5 (4-O-beta-Glucopyranosyl-cis-coumaric acid). **(B)** The proposed fragmentation pathways of 4-O-beta-Glucopyranosyl-cis-coumaric acid. **(C)** The MS/MS spectrum of compound 35 (dihydroactinidiolide). **(D)** The proposed fragmentation pathways of dihydroactinidiolide. **(E)** The MS/MS spectrum of compound 36 (magnolin). **(F)** The proposed fragmentation pathways of magnolin.

Compound 5 yielded [M-H]^-^ at *m/z* 325.0923 and [M-H + HCOO]^-^ at *m/z* 371.0987 in the negative ion mode. The fragment ion at *m/z* 163.0402 was produced from deprotonated ion owing to neutral elimination of a glucose unit (162 Da), and subsequent loss of one CO_2_ molecule from aglycone ion could also be detected at *m/z* 119.0504 (Figures 4A,B). Compound 5 was assigned as 4-O-beta-glucopyranosyl-cis-coumaric acid by comparison with the reference standard.

#### 3.1.4 Identification of terpene compounds

Compound 35 exhibited a [M + H]^+^ at *m/z* 181.1219, predominant fragment ions of *m/z* 153.1272 [M + H−2CH_2_]^+^, *m/z* 139.0754 [M + H−3CH_2_]^+^, *m/z* 137.0964 [M + H−C_2_H_4_O]^+^, and *m/z* 125.0966 [M + H−2CH_2_−CO]^+^ were observed (Figure 4C). By referring the literature data (Zeng et al., 2023), it was identified as dihydroactinidiolide by its characteristic fragmentation pattern (Figure 4D).

#### 3.1.5 Identification of phenylpropanoid compounds

Compound 36 gave a [M + H]^*+*^ ion at *m/z* 417.1919, the predominant ions at *m/z* 399.1796, 381.1695, 369.1691, and 368.1599 were monitored (Figure 4E). The fragment ions at *m/z* 399.1796 and *m/z* 369.1691 corresponded to the elimination of one H_2_O molecule followed by the loss of a neutral fragment with a molecule mass of 30 Da (-OCH_2_) from the protonated ion. The ion at *m/z* 381.1695 was produced by the successive loss of two H_2_O groups from [M + H]^*+*^ ion (Figure 4F). Compound 36 was tentatively attributed to magnolin by comparing the fragmentation pathway with literature data (Li, 2020).

The MS/MS spectra and proposed fragmentation pathways of other compounds were represented in Supplementary Figures S1–S36.

### 3.2 Prediction of compound targets

The target prediction for GWM compounds using BATMAN-TCM, DrugBank, PharmMapper, SuperPred, and SwissTargetPrediction identified 1,202 potential targets. The constructed GWM-ingredient-target network (Supplementary Figure S37) contains 1,246 nodes and 11,963 edges.

### 3.3 Asthma-related disease target identification

**FIGURE 5:**
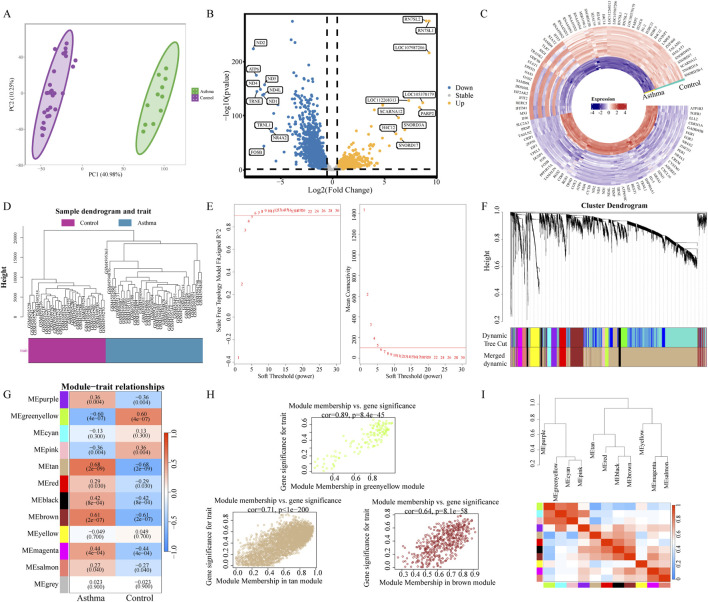
Identification of asthma-related disease targets. **(A)** PCA plot showing distinct clustering between asthma and control groups. **(B)** Volcano plot of DEGs: Yellow circles indicate upregulated genes, blue circles indicate downregulated genes, and gray circles represent non-significant genes; top 10 upregulated and top 10 downregulated genes labeled based on |log2FC|. **(C)** Heatmap visualization of the top 50 upregulated and downregulated DEGs between asthma and control samples. **(D)** Sample dendrogram and trait heatmap showing hierarchical clustering of samples with no outliers detected. **(E)** Plots of scale-free topology model fit (R^2^) and mean connectivity (right) for various soft-thresholding powers. A scale-free network was constructed when β was set to 6. **(F)** Gene clustering visualization depicting constructed vector complex modules in distinct colors, with a gene dendrogram demonstrating hierarchical sample clustering and module-color correspondence. **(G)** Heatmap of module-trait relationships, highlighting the correlation between individual modules and asthma-related traits. **(H)** Scatter plot showing the correlation between GS and MM for the asthma-associated module. Statistical significance was set at *P* < 0.05. **(I)** Gene module clustering tree and corresponding heatmap of module eigengene correlations, with greenyellow, tan, and brown modules showing strong associations.

GeneCards search using asthma identified 9,503 potential targets. PCA plot demonstrated clear separation between asthma and control groups (Figure 5A). Analysis of the GSE118875 dataset revealed 4,426 DEGs, with 2,195 upregulated and 2,231 downregulated genes. A volcano plot (Figure 5B) shows their distribution, and a heatmap (Figure 5C) highlights expression changes in the top 50 up- and downregulated genes between asthma and control groups.

After filtering the expression data, hierarchical clustering (Figure 5D) showed no outlier samples. A soft threshold power of 6 achieved scale-free topology (R^2^ > 0.9) (Figure 5E). Topological overlap matrix analysis identified 6 merged modules (Figure 5F), with greenyellow showing strong negative asthma correlation (r = −0.60, *P* < 0.01), while the tan and brown modules demonstrated positive correlations (r = 0.68, *P* < 0.01; r = 0.61, *P* < 0.01, respectively) (Figure 5G). Scatter plots confirmed a strong relationship between Gene Significance (GS) and Module Membership (MM) (Figure 5H), while correlation analysis demonstrated significant inter-module relationships (Figure 5I).

### 3.4 Identification of overlapping disease and compound targets and PPI network analysis

**FIGURE 6:**
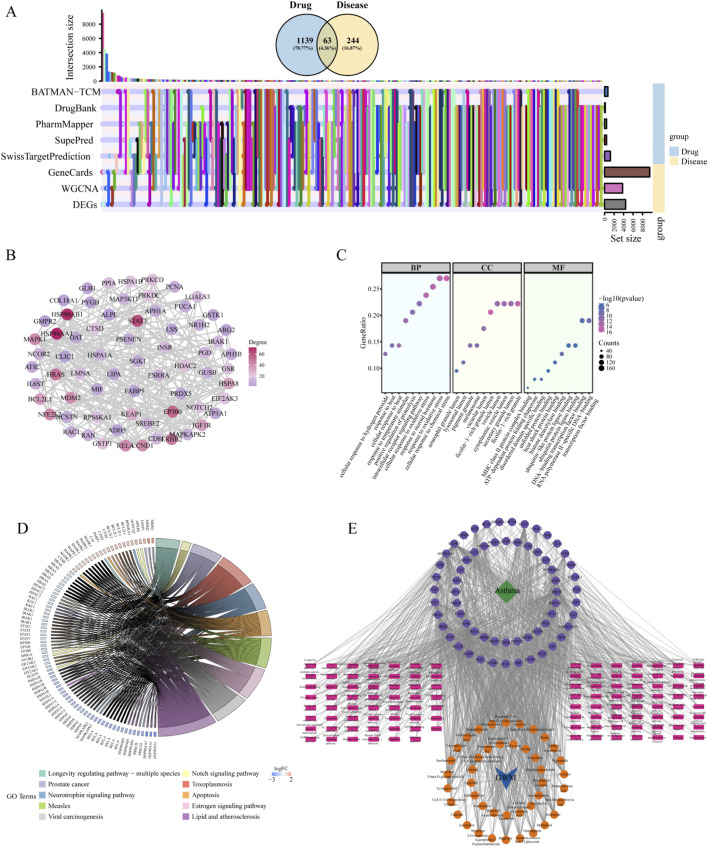
Network pharmacology analysis. **(A)** UpSet plot showing gene sets distribution and Venn diagram of intersection of 63 overlapping targets between GWM and asthma. **(B)** PPI network of the 63 overlapping targets. The color gradient from purple to red indicates the degree of node connectivity, with darker red representing higher degree values and darker purple representing lower degree values. **(C)** GO enrichment analysis of overlapping targets. **(D)** KEGG pathway enrichment analysis. **(E)** Integrated drug-compounds-targets-pathways-disease interaction network. Blue V-shape represents GWM (drug), orange circles represent active compounds, purple circles represent overlapping target genes, pink rectangles represent enriched pathways, and green square represents asthma.

Integration of GeneCards, DEGs and WGCNA identified 307 disease targets (Supplementary Figure S38), with UpSetR analysis revealing 63 compound-disease overlapping targets (Figure 6A). STRING-based PPI network construction visualized these 63 targets in Figure 6B, where node connectivity is visualized, with darker red indicating higher degree values and darker purple representing lower degree values.

### 3.5 GO and pathway enrichment analysis

In Figure 6C, GO analysis identifies overlapping targets participating in key biological processes such as cellular response to chemical stress, oxidative stress, and steroid hormone response. These genes are associated with cellular components like ficolin-1-rich granule lumen and secretory granule lumen, and they exhibit molecular functions such as MHC class II protein complex binding. KEGG pathway analysis (Figure 6D) revealed significant enrichment in pathways, particularly Lipid and atherosclerosis, the Notch signaling pathway, and Apoptosis.

### 3.6 Construction of drug-compounds-targets-pathways-disease interaction network

Figure 6E illustrates a comprehensive network depicting the relationships among GWM, its compounds, target genes, pathways, and asthma. The network displays a blue V-shape for drug, orange circles for ingredients, pink rectangles for pathways, purple circles for overlapping targets, and a green square for disease.

### 3.7 Identification of hub genes

**FIGURE 7:**
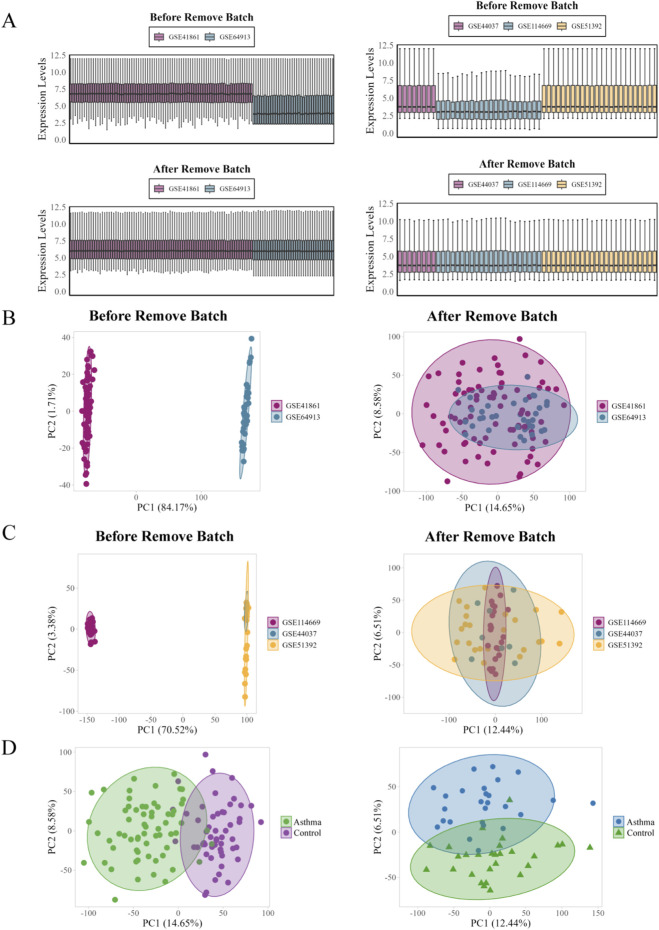
PCA and boxplot analysis of GEO datasets before and after batch effect removal. **(A)** Boxplots demonstrating gene expression distribution before and after batch effect removal. **(B)** Two-dataset PCA visualization before (Left) and after (Right) batch effect removal. **(C)** Three-dataset PCA visualization before (Left) and after (Right) batch effect removal. **(D)** PCA plots comparing asthma and control groups (Left: GSE44061 and GSE64913; Right: GSE114669, GSE44067, and GSE53192).

Figures 7A–D shows PCA and boxplots for GEO datasets before and after batch effect removal and outlier exclusion. Data cleaning minimized the differences between batches, while highlighting significant differences in the distribution of the asthma and control groups.

**FIGURE 8:**
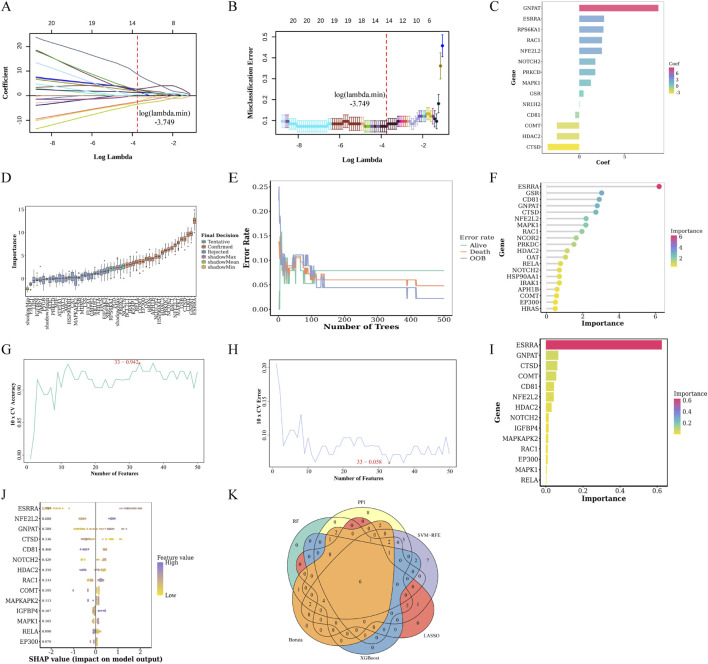
Identification of hub genes through multi-algorithm machine learning and PPI network analysis. **(A)** LASSO regression coefficient profiles. **(B)** Cross-validation error against log (lambda) with the optimal lambda value (vertical dashed line) at the point of minimal error. **(C)** Feature importance from LASSO regression. **(D)** Boruta algorithm result showing confirmed targets (orange). **(E)** The error rate with the number of trees. **(F)** Top 20 genes based on RF algorithm. **(G)** Cross-validation accuracy curve for SVM-RFE algorithm. **(H)** Cross-validation error rate for SVM-RFE algorithm. **(I)** Identified genes feature importance based on XGBoost algorithm. **(J)** SHAP value plot showing feature impact on model predictions. **(K)** Venn diagram showing overlap of key targets identified by different algorithms and PPI network analysis.

LASSO regression with 10-fold cross-validation generated coefficient profiles (Figure 8A) and plotted cross-validation errors against log (lambda) (Figure 8B). The optimal parameter (lambda.min) minimized misclassification error (Figure 8B), identifying 14 features. Figure 8C shows LASSO feature importance, while Figure 8D presents Boruta analysis results, confirming 22 significant targets. Using the RF algorithm, Figure 8E demonstrates decreasing error rates as tree numbers increase, stabilizing thereafter. Figure 8F ranks the top 20 targets. SVM-RFE identified 33 key targets, with Figures 8G,H showing that selecting 33 variables minimizes predictive error (5.8%) and maximizes accuracy (94.2%). XGBoost identified 14 key targets and ranked them by Gain values (Figure 8I), while Figure 8J visualizes SHAP values to assess feature impacts. PPI network analysis pinpointed the top 30 targets by degree centrality. Combining all model features and PPI rankings revealed six hub genes: CD81, NFE2L2, CTSD, NOTCH2, HDAC2, and MAPK1 (Figure 8K). All identified key targets are summarized in Supplementary Table S3.

**FIGURE 9:**
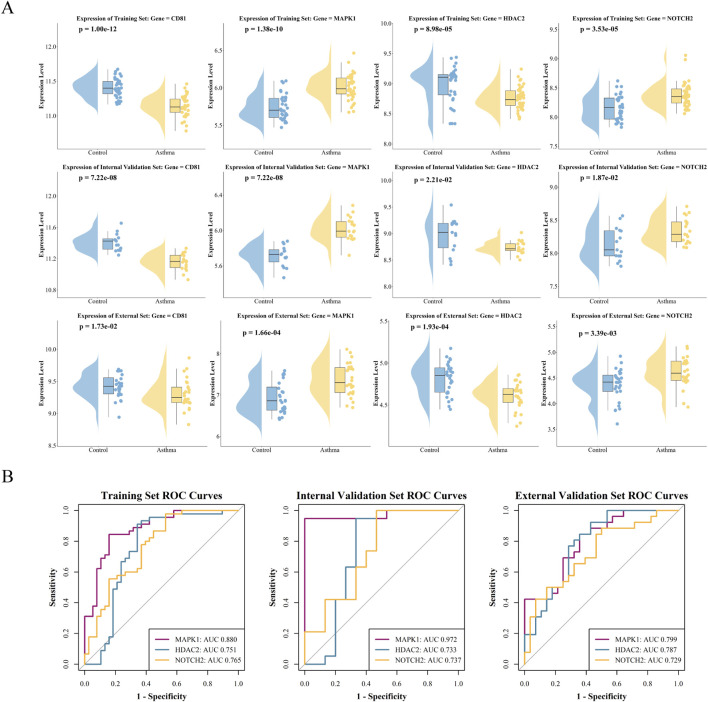
Expression and diagnostic performance of hub genes in the training, internal validation, and external validation datasets. **(A)** Differential expression analysis of core target genes comparing asthma patients and healthy controls. Each point represents the gene expression level of an individual sample, visualized using a combination of violin and box plots. Statistical significance was determined by the Wilcoxon rank-sum test (*P* < 0.05). **(B)** ROC curve analysis evaluating the diagnostic performance of the hub genes. The discriminative power is quantified by the Area Under the Curve (AUC) and 95% Confidence Intervals (CI). The AUC (95% CI) values in the training set were: MAPK1 0.880 (0.806–0.954), HDAC2 0.751 (0.630–0.872), and NOTCH2 0.765 (0.660–0.870). In the internal validation set, the values were: MAPK1 0.972 (0.915–1.000), HDAC2 0.733 (0.525–0.942), and NOTCH2 0.737 (0.556–0.918). In the external validation set, the values were: MAPK1 0.799 (0.683–0.915), HDAC2 0.787 (0.665–0.909), and NOTCH2 0.729 (0.594–0.865).

CD81, MAPK1, HDAC2, and NOTCH2 showed significant regulation in asthma patients across training, internal validation, and external validation datasets (all *P* < 0.05; Figure 9A). NOTCH2 and MAPK1 were upregulated, while HDAC2 and CD81 were downregulated. Additionally, MAPK1, HDAC2, and NOTCH2 exhibited strong discriminatory power (AUC >0.7) in all three datasets (Figure 9B).

In summary, the 1202 GWM targets were intersected with 307 asthma-related targets to yield 63 overlapping targets. These candidates were then sequentially filtered based on their degree of connectivity in the PPI network (top 30), five machine learning algorithms (6 candidates), and expression/ROC validation. This stepwise screening ultimately identified NOTCH2, HDAC2, and MAPK1 as the 3 hub genes.

### 3.8 Predictive nomogram and validation

**FIGURE 10:**
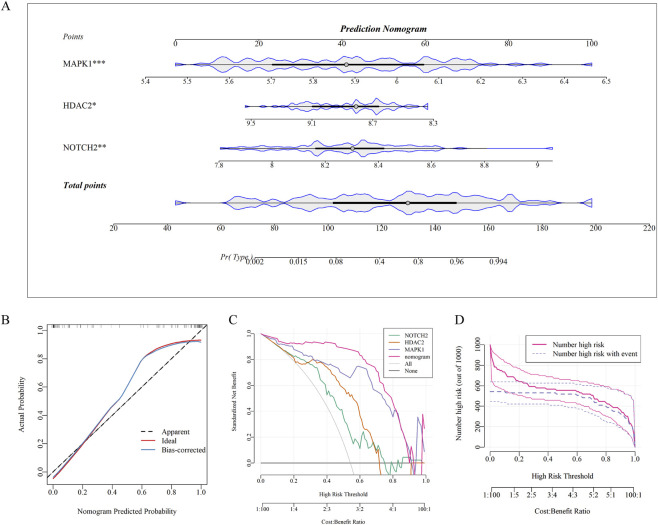
Development and validation of the nomogram for asthma risk prediction. **(A)** Nomogram model for hub genes, consisting of four horizontally aligned panels. Each panel represents the scoring system for an individual hub gene, along with the total score. The bottom x-axis indicates the asthma risk probability calculated from the total score. **(B)** Calibration curves assessed via bootstrap resampling with 1,000 iterations. The proximity to the red ideal line demonstrates the accuracy of nomogram predictions. **(C)** DCA showing net benefits of the combined nomogram compared with individual gene models (NOTCH2, HDAC2, and MAPK1) and baseline strategies (“Treat All” and “Treat None”) across a risk threshold range of 0–1. **(D)** CIC demonstrating the model’s ability to identify high-risk populations across a risk threshold range of 0–1 (assuming a standardized population size of 1,000).

A diagnostic nomogram incorporating the hub genes NOTCH2, HDAC2, and MAPK1 was developed to predict asthma occurrence (Figure 10A). Each gene was assigned a score based on its expression level, with the total score indicating cumulative disease risk. The predictive performance of the nomogram was evaluated using calibration curves (Figure 10B) and DCA (Figure 10C). The calibration curves showed minimal deviation between predicted and observed probabilities, indicating high accuracy. DCA confirmed the clinical value, as it provided greater net benefit compared to single-gene models, supporting its utility in clinical decision-making. At lower high-risk thresholds (Figure 10D), the nomogram identified more high-risk cases but had higher false positives; increasing the thresholds improved accuracy and reduced false positives, though fewer high-risk individuals were detected.

### 3.9 eQTL-based MR reveals hub genes for asthma risk

**FIGURE 11:**
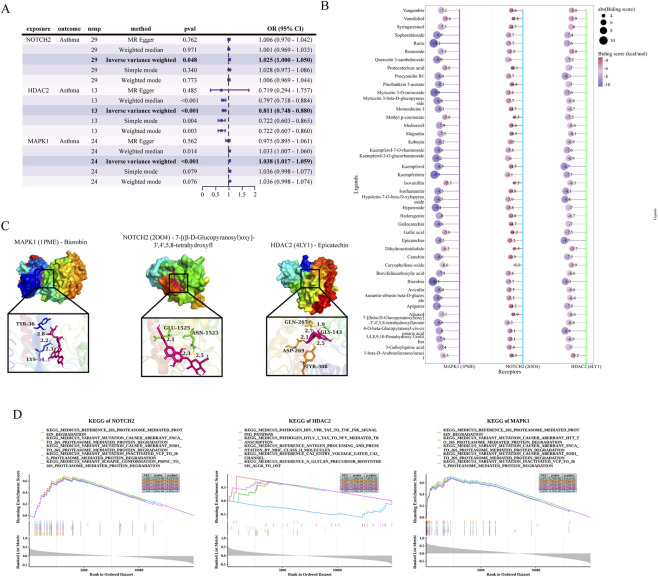
Causal effects, molecular docking, and GSEA of hub genes in asthma. **(A)** Forest plot demonstrating the causal effects of hub genes on asthma risk. **(B)** Molecular docking analysis showing binding scores between GWM components and three hub target proteins (MAPK1, PDB: 1PME; NOTCH2, PDB: 2OO4; and HDAC2, PDB: 4LY1). **(C)** 3D visualization of binding sites and molecular interactions for the hub gene-compound complexes (left to right): MAPK1 (PDB: 1PME) with Biorobin, NOTCH2 (PDB: 2OO4) with 7-[(β-D-Glucopyranosyl)oxy]-3',4',5,8-tetrahydroxyflavone, and HDAC2 (PDB: 4LY1) with Epicatechin. **(D)** GSEA results showing top five significantly enriched pathways ranked by |NES|, which were identified by ranking all genes based on their Spearman correlation coefficients with the respective hub target.

The causal effects of hub gene expression on asthma risk were assessed using the IVW method. NOTCH2 expression was positively associated with asthma risk (OR = 1.025, 95% CI = 1.000–1.050, *P* < 0.05), while HDAC2 showed an inverse association (OR = 0.811, 95% CI = 0.748–0.880, *P* < 0.01). MAPK1 expression also exhibited a significant positive correlation (OR = 1.038, 95% CI = 1.017–1.059, *P* < 0.01) (Figure 11A). Sensitivity analyses confirmed these findings’ robustness, with no horizontal pleiotropy detected by the MR-Egger intercept test (all *P* > 0.05). Heterogeneity was absent after removing outliers, as indicated by Cochran’s *Q* test (all *P* > 0.05). Reverse causality was excluded via Steiger filtering (all *P* < 0.001). Supporting plots, such as scatter, funnel, forest, and leave-one-out sensitivity analyses, further validated these associations (Supplementary Figures S39–S41).

### 3.10 Molecular docking results

Figure 11B shows binding energies of GWM components with three hub targets (MAPK1, PDB ID: 1PME; NOTCH2, PDB ID: 2OO4; and HDAC2, PDB ID: 4LY1). Lower energies indicate stronger interactions. Values < −7 kcal/mol suggest strong binding, while < −4 kcal/mol indicate moderate affinity. Notably, 1PME with Biorobin (−10.5 kcal/mol), 2OO4 with 7-[(β-D-Glucopyranosyl)oxy]-3',4',5,8-tetrahydroxyfl (−6.2 kcal/mol), and 4LY1 with Epicatechin (−8.7 kcal/mol) exhibit the strongest binding to their respective targets. Figure 11C illustrates the 3D structures, showcasing binding sites and conformations of target proteins interacting with these molecules.

### 3.11 Gene set enrichment analysis

Figure 11D shows the KEGG analysis results for NOTCH2, HDAC2, and MAPK1. NOTCH2 and MAPK1 were significantly enriched in the 26S proteasome-mediated protein degradation pathway, while HDAC2 primarily participated in the antigen processing and presentation via MHC class II molecules pathway.

### 3.12 Experimental validation

#### 3.12.1 Pulmonary histopathological analysis

**FIGURE 12:**
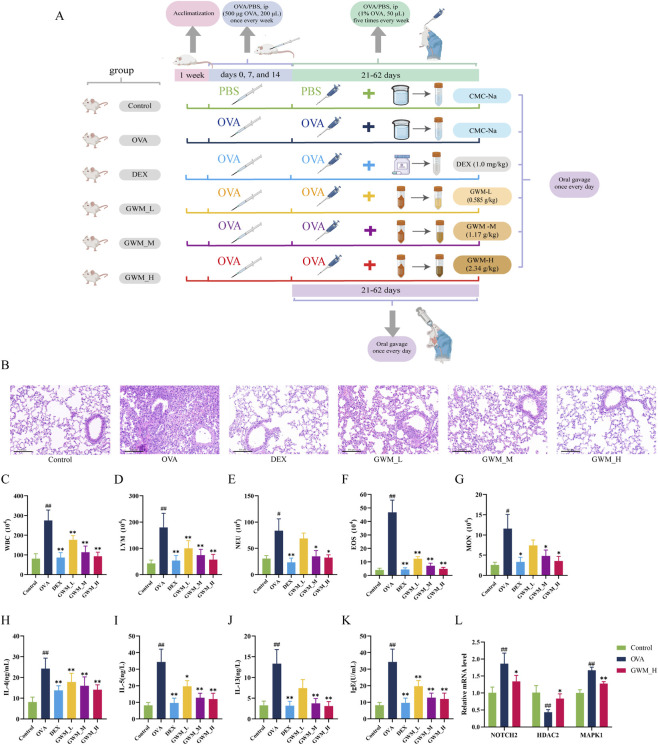
Experimental validation in an OVA-induced asthma model. **(A)** Experimental timeline showing the sensitization and challenge phases in the asthma model development. **(B)** HE staining of lung sections. Scale bar: 100 μm. **(C–G)** Analysis of inflammatory cells in BALF: **(C)** Total WBC, **(D)** Lymphocytes, **(E)** Neutrophils, **(F)** Eosinophils, and **(G)** Monocytes. Data presented as mean ± SD. ^##^ *P* < 0.01, ^#^ *P* < 0.05 compared with Control group; ^**^ *P* < 0.01, ^*^ *P* < 0.05 compared with OVA group. **(H–K)** Cytokine and IgE levels in serum and BALF: Serum IL-4 levels (**(H)**, unit: ng/mL) and BALF cytokine concentrations of IL-5 (**(I)**, unit: ng/L), IL-13 (**(J)**, unit: ng/L), and IgE (**(K)**, unit: U/mL). Data presented as mean ± SD. ^##^ *P* < 0.01, ^#^ *P* < 0.05 compared with Control group; ^**^ *P* < 0.01, ^*^ *P* < 0.05 compared with OVA group. **(L)** RT-qPCR analysis of hub genes (NOTCH2, MAPK1, and HDAC2) mRNA expression levels in Control, OVA, and GWM_H groups. Data presented as mean ± SD. ^##^ *P* < 0.01, ^#^ *P* < 0.05 compared with Control group; ^**^ *P* < 0.01, ^*^ *P* < 0.05 compared with OVA group.

Histological analysis (Figure 12B) showed normal bronchial and vascular morphology with minimal inflammation in the control group. OVA exposure caused airway remodeling, including narrowed airway lumina, thickened airway walls, and extensive inflammatory cell infiltration predominantly composed of eosinophils, and fibrosis of the alveolar walls. Dexamethasone treatment alleviated these changes, while GWM demonstrated dose-dependent effects.

#### 3.12.2 Inflammatory cell infiltration in BALF

OVA challenge significantly increased total WBC counts and differential inflammatory cells (lymphocytes, neutrophils, eosinophils, monocytes) in BALF compared to the control group (*P* < 0.05; Figures 12C–G). Relative to the OVA group, DEX treatment effectively reduced all measured cell types with *P* < 0.05. GWM dose-dependently attenuated inflammation, with the high-dose group (GWM_H) showing showing maximal reduction at *P* < 0.05, confirming its therapeutic potential. Specific results are presented in Supplementary Table S4.

#### 3.12.3 Cytokine levels in BALF and serum

ELISA results showed that the OVA challenge significantly elevated serum IL-4 levels (Figure 12H) and BALF concentrations of IL-5, IL-13, and IgE (Figures 12I–K) compared to the control group (*P* < 0.01). Both DEX and GWM treatments markedly reduced these biomarkers (*P* < 0.01). GWM treatment demonstrated a dose-dependent suppression of serum IL-4 and BALF IL-5, IL-13, and IgE, with the GWM_H group exhibiting the greatest reductions compared to the OVA group (*P* < 0.01). These results suggest that GWM effectively attenuates OVA-induced Th2-driven inflammation. Specific results are presented in Supplementary Table S5.

#### 3.12.4 mRNA expression levels for hub genes

The control, OVA, and GWM_H groups were selected for real-time quantitative polymerase chain reaction (RT-qPCR) analysis due to the GWM_H group showing optimal therapeutic effects in managing asthma in OVA-challenged mice. In the OVA group, NOTCH2 and MAPK1 mRNA levels were significantly upregulated compared to the control group (*P* < 0.05), while HDAC2 expression was notably decreased (*P* < 0.05). High-dose GWM treatment reduced NOTCH2 and MAPK1 mRNA levels and increased HDAC2 expression compared to the OVA group (*P* < 0.05), as shown in Figure 12L and Supplementary Table S6. Amplification and melting curves for primers are shown in Supplementary Figures S42, 43.

### 3.13 Single-cell RNA sequencing analysis reveals dynamic changes and interactions of immune cell subpopulations

**FIGURE 13:**
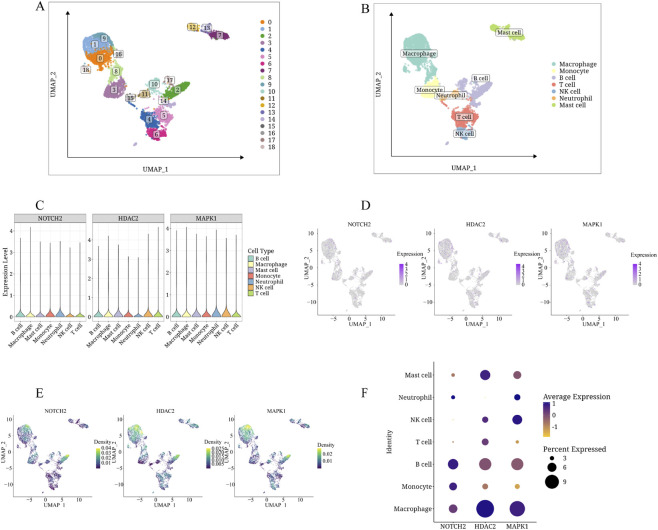
scRNA-seq data analysis. **(A)** UMAP visualization of Seurat clusters, revealing 18 distinct cell clusters. **(B)** Cellular annotations reveal seven distinct cell phenotypes. **(C)** Violin plots depicting the expression distribution of hub genes (NOTCH2, MAPK1, and HDAC2) across identified cell populations. **(D)** Feature plots showing the spatial distribution of hub gene expression mapped onto UMAP coordinates. **(E)** Density plots illustrating the expression intensity of hub genes across different cell types. **(F)** Dot plot representation showing the percentage of cells expressing each hub gene (dot size) and relative expression levels (color intensity) across different cell populations. The dot size reflects the percentage of cells with expression >0, while the color gradient indicates the scaled average expression (Z-score) centered at zero.

After quality control filtering, cellular expression profiles across samples are shown in Supplementary Figure S44A. PCA (Supplementary Figure S44B) indicated relatively stable cell distribution across samples. From Supplementary Figure S44C, D, the top 15 principal components were selected for analysis. UMAP analysis classified cells into 18 clusters (Figure 13A). Manual annotation using classical marker genes and the SingleR package identified six cell types: macrophage, monocyte, B cell, T cell, NK cell, neutrophil, and mast cell (Figure 13B). NOTCH2 is highly expressed in macrophages, neutrophils, and B cells (Figures 13C–E), with the highest average expression in B cells (Figure 13F). MAPK1 is expressed in macrophages, B cells, NK cells, and neutrophils, with macrophages showing the highest average expression. HDAC2 is highly expressed in T cells, NK cells, and macrophages, with macrophages having the highest average expression.

**FIGURE 14:**
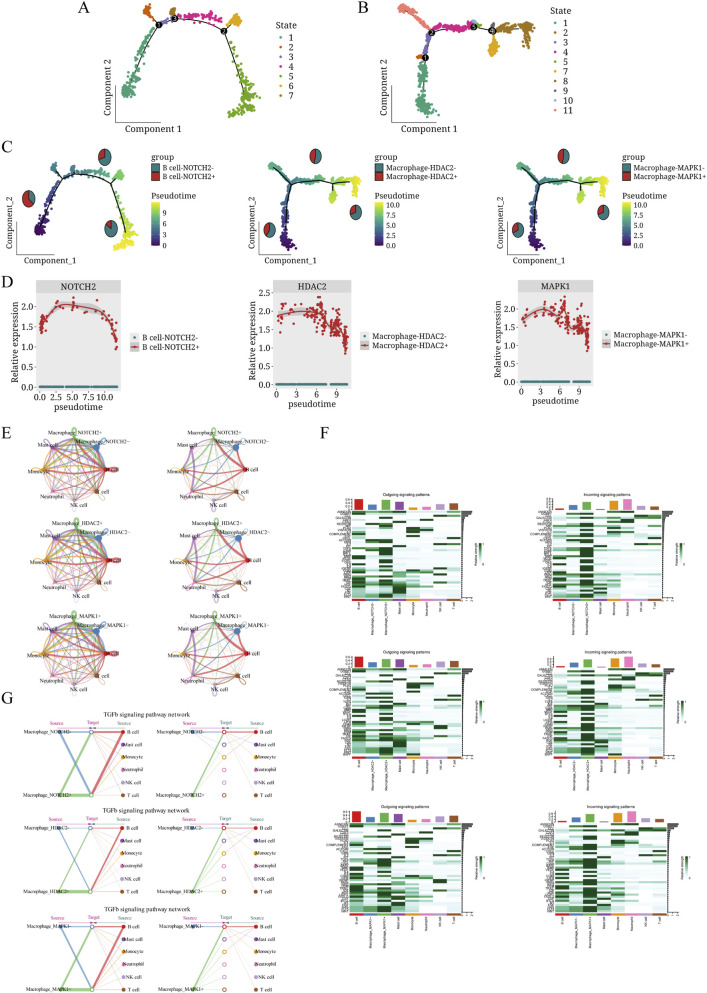
Cell trajectory and cell communication analysis. **(A)** Pseudo-time trajectory showing B cell developmental progression. Dots represent individual cells colored by subcluster identity, with numbered states along the trajectory. **(B)** Pseudo-time trajectory showing macrophage developmental progression. Dots represent individual cells colored by subcluster identity, with numbered states along the trajectory. **(C)** The cell proportions of NOTCH2^+^ B cells, HDAC2^+^ macrophages, and MAPK1^+^ macrophages shifted dynamically and in coordination as the quasitemporal process unfolded. **(D)** Pseudotime trajectories depicting the dynamic gene expression levels within NOTCH2^+^ B cells, HDAC2^+^ macrophages, and MAPK1^+^ macrophages during disease progression. **(E)** Circle visualization illustrating cellular interaction weights and the number of interactions between NOTCH2^+^ B cells, HDAC2^+^ macrophages, and MAPK1^+^ macrophages, and other cell types. **(F)** Signaling patterns between NOTCH2^+^ B cells, HDAC2^+^ macrophages, and MAPK1^+^ macrophages with other cell types are organized to show outgoing signaling on the left and incoming signaling on the right. **(G)** Hierarchical plot: The left portion depicts autocrine and paracrine signaling for the cells of interest (NOTCH2^+^ B cells, HDAC2^+^ macrophages, and MAPK1^+^ macrophages, arranged from top to bottom). The right portion shows autocrine and paracrine signaling patterns for other, secondary cell types within the dataset.

We segregated cells into HDAC2^+^ and HDAC2^-^ macrophage groups, MAPK1^+^ and MAPK1^-^ macrophage groups, as well as NOTCH2^+^ and NOTCH2^-^ B cell groups. Trajectory analysis revealed distinct developmental paths and pseudotime progression of cellular clusters (Figures 14A,B). Early stages showed predominant NOTCH2^+^ B cells with low HDAC2^+^ and MAPK1^+^ macrophage populations. As pseudotime progressed, HDAC2^+^ and MAPK1^+^ macrophages increased while NOTCH2^+^ B cells decreased. Later stages showed peak followed by decline in HDAC2^+^ and MAPK1^+^ macrophage populations (Figure 14C). In the pseudotemporal analysis, the relative expression levels of MAPK1 and NOTCH2 were shown in Figure 14D.

Cellular communication analysis identified NOTCH2^+^ B cells, HDAC2^+^ macrophages, and MAPK1^+^ macrophages as subpopulations with significantly enhanced outgoing and incoming interaction strengths compared to their negative counterparts. NOTCH2^+^ B cells displayed stronger interactions with both macrophages and monocytes, while HDAC2^+^ and MAPK1^+^ macrophages predominantly interacted more with monocytes (Figure 14E). Compared to their negative counterparts, MAPK1^+^ and HDAC2^+^ macrophages displayed stronger interaction likelihood, particularly in IL-4 pathways, while NOTCH2^+^ B cells showed enhanced interaction through LIGHT pathways (Figure 14F). In the TGF-β signaling network, HDAC2^+^ macrophages interacted more extensively with B cells than HDAC2^-^ macrophages, while MAPK1^+^ macrophages showed broader communication with B cells and mast cells in the FGF signaling network. NOTCH2^+^ B cells also had more interactions with macrophages in the TGFβ pathway compared to NOTCH2^-^ B cells (Figure 14G). Supplementary Figures S45–S47 illustrate ligand-receptor interactions between NOTCH2^+^ B cells, HDAC2^+^ macrophages, and MAPK1^+^ macrophages, as well as their negative counterparts, and other cell types.

### 3.14 Immune cell infiltration and correlation

**FIGURE 15:**
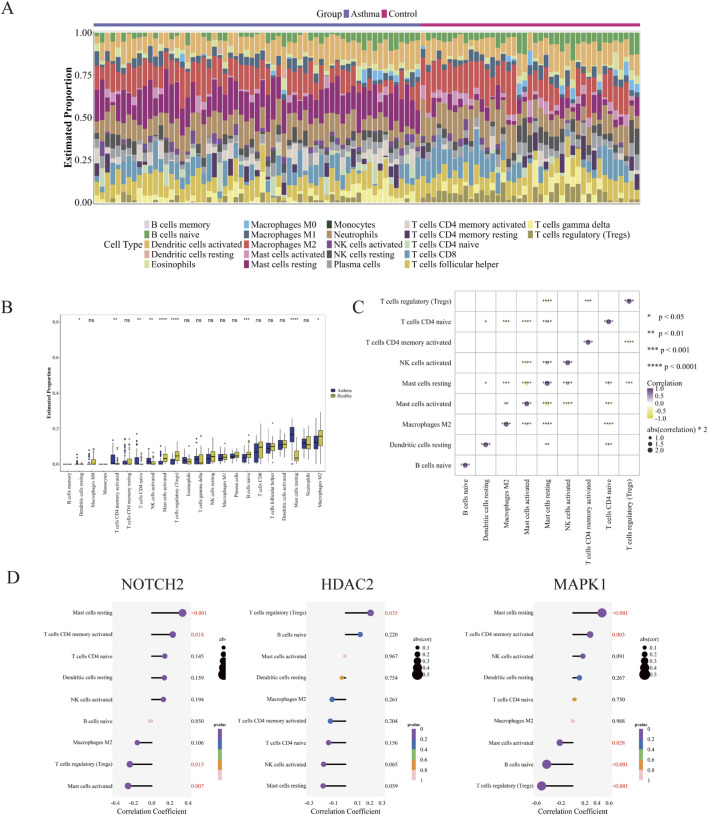
Immune cell infiltration and correlation analysis. **(A)** Bar charts of 22 immune cell proportions in asthma and control groups. **(B)** Differential analysis of immune cell types between asthma and control groups. ^****^ *P* < 0.0001, ^***^ *P* < 0.001, ^**^ *P* < 0.01, ^*^ *P* < 0.05, ns, no significant, Wilcoxon rank-sum test. **(C)** Correlation heatmap among the 22 immune cell types. **(D)** Lollipop plot depicting correlations between hub genes (NOTCH2, MAPK1, HDAC2) and differentially abundant immune cells.

Using the CIBERSORT method, we analyzed immune cell profiles in asthma and control groups, identifying relative proportions of 22 immune cell types (Figure 15A). The asthma group exhibited the highest levels of Mast cells resting, while the control group had the greatest proportions of Macrophages M2. Nine immune cell types showed significant differences between the groups: Mast cells resting, Tregs, Mast cells activated, B cells naive, T cells CD4 memory activated, NK cells activated, T cells CD4 naive, Dendritic cells resting, and Macrophages M2 (Figure 15B). Correlation analysis revealed a strong inverse relationship between Mast cells activated and Mast cells resting (cor = −0.755, *P* < 0.01) (Figure 15C). We explored the relationships between hub genes and differential immune cells. NOTCH2 positively correlated with Mast cells resting and T cells CD4 memory activated, but negatively associated with Mast cells activated and Tregs. Similarly, MAPK1 showed positive correlations with Mast cells resting and T cells CD4 memory activated but negative associations with Mast cells activated, B cells naive, and Tregs. HDAC2 was positively associated with Tregs (Figure 15D).

### 3.15 Results of mediation analysis

Reverse MR analysis found no causal effects of asthma risk on the expression of the three hub genes (all *P* > 0.05; Supplementary Figure S48). Sensitivity analyses indicated no heterogeneity (Cochran’s *Q* test) and no signs of horizontal pleiotropy (MR-Egger intercept method, all *P* > 0.05). Visualizations, including scatter plots, funnel plots, forest plots, and leave-one-out analyses, are shown in Supplementary Figures S49–S51.

MR analysis revealed that changes in NOTCH2, HDAC2, and MAPK1 genes directly influenced immune cell features (Supplementary Figure S52). NOTCH2 levels were positively correlated with CD25^++^ CD45RA^−^ CD4 not regulatory T cell %CD4^+^ T cell (OR = 1.062, 95% CI = 1.001–1.127, *P* < 0.05). Similarly, MAPK1 also exhibited a positive association with this subset (OR = 1.071, 95% CI = 1.003–1.145, *P* < 0.05). HDAC2 displayed positive associations with CD28 on CD39^+^ secreting CD4 regulatory T cell (OR = 1.114, 95% CI = 1.013–1.224, *P* < 0.05). No heterogeneity or horizontal pleiotropy was detected (all *P* > 0.05), supported by scatter, funnel, and forest plots, along with leave-one-out analyses (Supplementary Figures S53–S55).

CD25^++^ CD45RA^−^ CD4 not regulatory T cell %CD4^+^ T cell was positively correlated with asthma risk (OR = 1.024, 95% CI = 1.015–1.034, *P* < 0.01), while CD28 on CD39^+^ secreting CD4 regulatory T cell showed a negative correlation (OR = 0.978, 95% CI = 0.972–0.985, *P* < 0.01), as shown in Supplementary Figure S56. Given the presence of pleiotropy, an IVW random-effects model was employed. However, no pleiotropy was observed. Detailed scatter plots, funnel plots, forest plots, and leave-one-out sensitivity analyses are included (Supplementary Figures S57, S58).

The mediation effect of hub genes on asthma risk through immune cells, as shown in Supplementary Table S7, includes the following key findings: CD25^++^ CD45RA^−^ CD4 not regulatory T cell, as a proportion of CD4^+^ T cell, exhibited a mediated proportion of 5.98% for NOTCH2 expression levels. For the same cell subset, MAPK1 levels showed a mediated proportion of 4.48%. Additionally, CD28 on CD39^+^ secreting CD4 regulatory T cell displayed a mediated proportion of 1.12% in the context of HDAC2 levels influencing asthma risk.

## 4 Discussion

Asthma, a chronic respiratory disease characterized by airway inflammation and hyperresponsiveness, imposes substantial burdens on patients and healthcare systems. While current therapies like inhaled corticosteroids provide symptomatic control, herbal medicines continue to serve as complementary options due to their multi-component nature and holistic effects (Liu et al., 2016). GWM, a traditional medicinal plant, contains bioactive compounds with reported anti-inflammatory and immunomodulatory properties, yet its potential role in asthma remains uncharacterized. Using UPLC-QE-Orbitrap-MS, 43 compounds were identified in GWM, with some demonstrating potential for alleviating asthma symptoms and reducing AHR (Chen and Ko, 2021; Xu et al., 2023; Kim et al., 2006). It is widely recognized that botanical extracts often achieve enhanced therapeutic effects through the synergistic interactions of their multiple constituents, targeting multiple pathways simultaneously (Chen et al., 2024). Therefore, our research aims to evaluate GWM as a phytocomplex and identify its key therapeutic targets in asthma treatment through a comprehensive approach combining network pharmacology, machine learning, experimental validation, and MR.

Initial screening identified 9,503 asthma-related targets from GeneCards and 4,426 DEGs in asthmatic patients. WGCNA prioritized three gene modules strongly linked to asthma. By intersecting these sets, 307 high-confidence asthma targets were identified, with 63 overlapping GWM compound targets. PPI network analysis highlighted dense interactions, and machine learning-driven feature selection along with centrality analysis pinpointed NOTCH2, HDAC2, and MAPK1 as core therapeutic targets. Multi-cohort validation showed NOTCH2 and MAPK1 were significantly upregulated, while HDAC2 was downregulated in asthma (*P* < 0.05), all with strong diagnostic performance (AUC >0.7). Incorporating these targets into a multi-marker nomogram improved predictive accuracy for asthma, while MR analysis confirmed causal roles: NOTCH2 and MAPK1 as risk factors, and HDAC2 as protective factor. Molecular docking also indicated that these genes had strong binding affinity with GWM components, supporting their potential as candidate targets worthy of further experimental verification.

Functional enrichment reveals the Notch signaling pathway as an important mediator for GWM intervention in asthma pathogenesis. A pivotal mechanism in asthma involves the Th1/Th2 imbalance, where Notch signaling plays a crucial regulatory role. Specifically, Notch signaling in CD4^+^ T lymphocytes directly modulates this Th1/Th2 differentiation, and studies have shown that its inhibition can reduce airway inflammation and prevent AHR onset (Wang J. et al., 2025). Among the Notch receptors, NOTCH2 has emerged as a particularly significant player, showing marked upregulation in both asthma patients and experimental models (Tindemans et al., 2019; Zhang et al., 2023). NOTCH2, working in concert with NOTCH1, is essential for Th2 cell differentiation and the regulation of allergic airway inflammation. Studies have demonstrated that combined Notch1/Notch2 deficiency in T cells prevents allergic airway inflammation, an effect that persists even with forced GATA3 expression (Amsen et al., 2007; Tindemans et al., 2020). NOTCH2 is also critical for immune cell development and function, particularly in B cell lineage determination, where it guides the fate of transitional B cells through interaction with DLL-1 on follicular fibroblasts, determining their development into either follicular B cells or marginal zone B cells (Saito et al., 2003; Hozumi et al., 2004). A finding supported by scRNA-seq results that reveal predominant NOTCH2 expression in B cells. Beyond immune regulation, the Notch pathway is also involved in airway remodeling, a key pathological feature of asthma (Zhang et al., 2025). Through regulation of epithelial-mesenchymal transition and interactions with TGF-β and Wnt pathways and microRNAs, Notch signaling influences markers like E-cadherin and α-smooth muscle actin (Yang et al., 2017; Deshmukh et al., 2021). Knockdown studies targeting NOTCH2 showed reduced goblet cell hyperplasia, mucus production, and airway remodeling, underscoring its potential as a therapeutic target for asthma (Carrer et al., 2020).

HDAC2, a class I histone deacetylase, plays a crucial role in regulating inflammatory responses in asthma, with its activity significantly reduced in alveolar macrophages and blood mononuclear cells of asthma patients compared to healthy individuals (Cosío et al., 2004; Potaczek et al., 2024). Consistent with the importance of HDAC2 in macrophage function, our scRNA-seq analysis demonstrated that macrophages exhibit the highest average expression of HDAC2 among all cell types, highlighting the potential significance of maintaining proper HDAC2 levels in macrophages for effective immune responses. The reduction in HDAC2 activity is intricately linked to the oxidative stress response, a key driver of inflammation in asthma. Specifically, inflammatory cells in asthmatic airways produce excessive reactive oxygen species (ROS) in response to environmental triggers such as pollutants, infections, and smoke (Jesenak et al., 2017; Vincenzo et al., 2023). Excessive ROS generation activates p38 mitogen-activated protein kinase (p38MAPK), which phosphorylates and inactivates the glucocorticoid receptor while simultaneously suppressing HDAC2 (Mishra et al., 2018; Bi et al., 2020). Additionally, ROS excess activates phosphoinositide 3-kinase δ (PI3Kδ), further inhibiting HDAC2 and driving inflammation through T cell signaling and mast-cell activity (Mukherjee et al., 2017; Bi et al., 2020). These mechanisms are highly consistent with our GO analysis results, which indicate that oxidative stress response is significantly involved in asthma pathogenesis and can be modulated by GWM intervention. Beyond immune modulation, TGF-β1 signaling suppresses MUC5AC production in airway epithelial cells through SMAD3/HDAC2 complex-mediated NF-κB deacetylation, modulating mucus hypersecretion (Lee et al., 2021). Interestingly, our scRNA-seq analysis revealed that HDAC2^+^ macrophages exhibited enhanced interactions with B cells compared to HDAC2^-^ macrophages in the TGF-β signaling pathway network, suggesting a broader role of HDAC2 in both epithelial regulation and immune cell communication within the TGF-β signaling context.

MAPK1 (also known as ERK2) is a crucial signaling protein that plays a significant role in asthma pathophysiology through multiple mechanisms. Research indicates that the cg11335969 locus shows reduced methylation in both regular and refractory asthma, which may lead to MAPK1 overexpression (Lin and Yang, 2023). Whereas downregulating MAPK1, for instance through interventions such as allergen reduction, can decrease the secretion of Th2 inflammatory factors and mitigate airway inflammation (Xia et al., 2022). Additionally, knockdown of MAPK1 has been shown to suppress neutrophil, dendritic cell, and macrophage infiltration in asthma, underscoring its pivotal role in mediating immune responses (Lin and Yang, 2023). Consistently, scRNA-seq results reveal that macrophages exhibit the highest average expression of MAPK1, further highlighting the crucial role of this molecule in driving macrophage-mediated immune responses in asthma. Furthermore, MAPK1/3 isoforms are involved in the TGF-β1 signaling pathway via ALK5 receptors, functioning independently of Smad proteins to inhibit collagenase, matrix metalloproteinases, MHC class II antigen expression, and surfactant synthesis in type II pneumocytes (Panek et al., 2016, Panek et al., 2022).

The GSEA pathway enrichment analysis highlighted that NOTCH2 and MAPK1 are primarily associated with the 26S proteasome-mediated protein degradation pathway, which may reflect their shared role in orchestrating protein turnover critical for immune cell activation. Under physiological conditions, the NF-κB pathway is regulated by IκB, which undergoes phosphorylation, ubiquitination, and subsequent proteasomal degradation, allowing NF-κB activation (Yaagoubi et al., 2021). While this mechanism has not been specifically studied in the context of asthma, the inhibition of PI3K/AKT/mTOR and TLR4/MyD88/NF-κB signaling pathways has been shown to contribute to reducing airway inflammation and mitigating asthma symptoms (Ma et al., 2021). Notably, the MHC class II molecules pathway aligns with our scRNA-seq findings of HDAC2^+^ macrophage-B cell crosstalk, potentially modulating Th2 epitope presentation. This alignment can be attributed to the critical function of macrophages in antigen processing. As professional antigen-presenting cells, macrophages are responsible for processing antigens and loading them onto MHC class II molecules (Kawasaki et al., 2022). This process enables the presentation of extracellular antigens to CD4^+^ T cells, thereby driving adaptive immune responses (Pishesha et al., 2022).

Furthermore, the potential therapeutic effects of these hub genes were further verified through animal experiments in OVA-induced asthmatic mice. In this study, GWM treatment effectively reduced OVA-induced inflammatory cell aggregation in asthmatic mice. Since inflammatory leukocytes, including eosinophils, neutrophils, lymphocytes, and monocytes, critically contribute to asthma pathogenesis by amplifying airway inflammation and hyperresponsiveness through their recruitment and activation during the allergen-driven Th2 cytokine cascade (Bao and Zhu, 2022), we assessed inflammatory cell infiltration to further elucidate the potential mechanisms underlying the anti-inflammatory effects of GWM treatment. IL-4, IL-5, and IL-13 are key in asthma pathogenesis, driving Th2 inflammation and IgE production. IL-4, considered the master Th2 switch, plays a central role by inducing IgE production in plasma cells and upregulating various immune cell receptors (Yamanishi et al., 2017; Yip et al., 2021). It also promotes the generation of other pro-allergic cytokines, including IL-5 and IL-13 (Nur Husna et al., 2022). Working in concert, IL-4 and IL-13 activate B cells to synthesize IgE, trigger airway hyperresponsiveness, and induce goblet cell hyperplasia and mucus hypersecretion (Wills-Karp and Finkelman, 2008; Sahoo et al., 2016). Therefore, we measured the levels of IL-4, IL-5, IL-13, and IgE in our study. Our results demonstrated that GWM treatment dose-dependently inhibited OVA-induced expression levels of the correlator in asthmatic mice, indicating its potential therapeutic effects in mitigating Th2 cytokine-mediated inflammation and the associated airway remodeling and hyperresponsiveness in asthma. Asthma is characterized by airway remodeling, including thickening of airway walls due to increased airway smooth muscle mass, glandular hypertrophy, connective tissue deposition, edema, and inflammatory cell infiltration (Huang and Qiu, 2022). Our findings demonstrated that mice in the model group exhibited significant airway remodeling, characterized by luminal narrowing, airway wall thickening, and prominent inflammatory cell infiltration surrounding the bronchi and blood vessels. Treatment with GWM resulted in dose-dependent mitigation of these pathological changes, with higher doses showing greater therapeutic effects. To further elucidate the molecular mechanisms underlying the therapeutic effects of GWM, we evaluated the mRNA expression of hub genes involved in asthma pathogenesis. Our analysis revealed significant upregulation of NOTCH2 and MAPK1, alongside decreased expression of HDAC2, in the model group. However, after GWM treatment, these expression patterns were altered. These changes in gene expression align with the established roles of these genes in promoting airway inflammation and remodeling.

This study pioneers the integration of MR mediation analysis to disentangle the causal pathways linking genetic variants, immunophenotypes, and asthma risk. Our findings reveal that MAPK1 and NOTCH2 mediate the expansion of CD25^++^ CD45RA^−^ CD4 not regulatory T cells as a proportion of CD4^+^ T cells, contributing to the increased risk of asthma, while HDAC2 regulates CD28 on CD39^+^ secreting CD4 regulatory T cells, thereby influencing asthma risk. These genetic to immune cell regulations align with the immune cell infiltration findings, thereby reinforcing their potential contributions to asthma pathogenesis. These results advance current understanding of asthma pathogenesis by mapping molecular drivers to specific immune cell subsets, offering mechanistic insights with translational implications.

Naive T cells express CD45RA, but when activated and differentiated, they transition to expressing CD45RO (Machura et al., 2008). This transition indicates that CD45RA^−^ T cells have already undergone activation and are not in their naive state. While CD25^high^ expression is associated with natural Treg cells, there are also cells that express CD25 which are not Tregs (Hoffmann et al., 2006). After antigen encounter, T cells lose CD45RA and become either central memory or effector memory cells, both being CD45RA^−^ (Carrasco et al., 2006). Therefore, CD25^++^ CD45RA^−^ CD4 not regulatory T cell could represent a subset of activated CD4^+^ T cells with a memory or effector phenotype, such as Th1 and Th2 subsets, which play an important role in chronic airway inflammation and immune dysregulation in asthma. In allergic asthma, airway inflammation is characterized by Th2 cells and type 2 innate lymphoid cells producing Th2-associated cytokines such as IL-4, IL-13, and IL-5, along with mast cell activation, eosinophil infiltration, and increased IgE production by B cells (León and Ballesteros-Tato, 2021). This cellular activity is reflected during acute asthma exacerbations, when Th2 cell markers in peripheral blood rise (Shrestha Palikhe et al., 2021). As mentioned above, CD25^++^ CD45RA^−^ CD4 not regulatory T cell are implicated in asthma pathogenesis and contribute to inflammatory processes. Our MR analysis identifies both MAPK1 (ERK2) and NOTCH2 as promoters of CD25^++^ CD45RA^−^ CD4 not regulatory T cells, with higher levels of these cells correlating positively with the proportion of CD4^+^ T cells and increased asthma risk. GWM reduced OVA-induced increases in MAPK1 and NOTCH2 expression, and its therapeutic effect is likely achieved by downregulating the expression of these molecules, which subsequently suppresses the expansion of CD25^++^ CD45RA^−^ CD4 not regulatory T cell relative to the CD4^+^ T cell population.

Tregs employ multiple mechanisms to suppress allergic inflammation. CD39 on Tregs participates in immunosuppressive activity by converting ATP into adenosine, while CD28 serves as a crucial costimulatory molecule in T cell activation (Timperi and Barnaba, 2021). CTLA4 on Tregs competes with CD28 on effector T cells for binding to CD80/CD86, blocking CD28 costimulation and thereby inhibiting antigen presenting cells from activating effector T cells (Huang N. et al., 2020). Tregs preferentially sequester IL-2 via constitutively high IL-2R (CD25) expression, limiting this proliferation factor for effector T cells (Huang N. et al., 2020). Additionally, Tregs suppress the activation and function of eosinophils, basophils, mast cells, NKT cells, and ILC2s, and reduce IgE production by B cells (Zhang et al., 2022). Given the critical role of these molecular mechanisms in Treg function, understanding their regulation is essential for developing therapeutic strategies for allergic diseases. Through mediation analysis, HDAC2 influences CD28 expression on CD39^+^ secreting CD4 regulatory T cells, which in turn affects asthma severity. GWM reversed OVA-induced HDAC2 downregulation, suggesting that its therapeutic effect is likely achieved by upregulating HDAC2 expression, which subsequently suppresses CD28 expression on CD39^+^ secreting CD4 regulatory T cells.

Despite integrating multiple methodologies, several limitations remain. Reliance on public datasets introduces potential biases and limits generalizability due to the exclusive use of European-ancestry GWAS data. The murine model recapitulates Th2-dominant asthma but does not represent the full spectrum of human disease or endotypes; future studies will address this by expanding the sample size and comparing findings across additional asthma models e.g., IL-33-driven or HDM-induced asthma. Mediation effects observed were modest, and the effects of GWM on specific immune cell populations were not directly validated *in vivo*; subsequent experiments involving flow cytometric analysis of relevant immune cell subsets deserve further validation. Additionally, this study did not include dedicated safety assessments such as detailed toxicological or histopathological analyses; nevertheless, no deaths or overt adverse effects were observed in the present study. Comprehensive safety evaluations will be conducted in future studies. The study also did not quantify active compounds in the GWM extract, confirm direct target binding, or assess pharmacokinetics, leaving uncertainties about bioavailability and mechanism; ongoing work will include comprehensive quantification of bioactive compounds, surface plasmon resonance or cellular thermal shift assays, as well as pharmacokinetic profiling to address these gaps.

## 5 Conclusion

This study is the first to reveal the therapeutic potential of GWM in asthma treatment, expanding its application beyond the rheumatism and dermatological disorders previously reported. By integrating network pharmacology, machine learning, Mendelian randomization, and experimental validation, we explored the possible mechanisms underlying its effects. We identified 43 bioactive compounds in GWM that collectively target multiple pathological pathways in asthma. The hub targets NOTCH2, HDAC2, and MAPK1 were verified to play crucial roles in asthma pathogenesis by regulating immune responses and airway remodeling. Single-cell RNA sequencing further elucidated the cellular mechanisms underlying the immunomodulatory effects of GWM. These findings not only advance our understanding of the pharmacological mechanisms of GWM but also emphasize the importance of combining computational and experimental approaches to investigate the molecular basis of herbal medicine.

## Data Availability

The original contributions presented in the study are included in the article/Supplementary Material, further inquiries can be directed to the corresponding authors.

## References

[B1] Amsen D. Antov A. Jankovic D. Sher A. Radtke F. Souabni A. (2007). Direct regulation of Gata3 expression determines the T helper differentiation potential of Notch. Immunity 27 (1), 89–99. 10.1016/j.immuni.2007.05.021 17658279 PMC2062505

[B2] Anderson I. I. W. C. Baptist A. P. Eakin M. N. Federman A. Murphy V. E. (2024). Adherence challenges and strategies in specific groups with asthma: adolescents, pregnancy, and older adults. J. Allergy Clin. Immunol. Pract. 12 (12), 3216–3222. 10.1016/j.jaip.2024.07.031 39122111

[B3] Bao Y. Zhu X. (2022). Role of chemokines and inflammatory cells in respiratory allergy. J. Asthma Allergy 15, 1805–1822. 10.2147/JAA.S395490 36575714 PMC9790160

[B4] Barnes P. J. (2013). Corticosteroid resistance in patients with asthma and chronic obstructive pulmonary disease. J. Allergy Clin. Immunol. 131 (3), 636–645. 10.1016/j.jaci.2012.12.1564 23360759

[B5] Bi J. Min Z. Yuan H. Jiang Z. Mao R. Zhu T. (2020). PI3K inhibitor treatment ameliorates the glucocorticoid insensitivity of PBMCs in severe asthma. Clin. Transl. Med. 9 (1), 22. 10.1186/s40169-020-0262-5 32112175 PMC7048898

[B6] Bian B. Kelton C. M. L. Wigle P. R. Guo J. J. (2010). Evaluating safety of long-acting beta agonists (LABAs) in patients with asthma. Curr. Drug Saf. 5 (3), 245–250. 10.2174/157488610791698316 20210730

[B7] Burgess S. Bowden J. Fall T. Ingelsson E. Thompson S. G. (2017). Sensitivity analyses for robust causal inference from Mendelian randomization analyses with multiple genetic variants. Epidemiology 28 (1), 30–42. 10.1097/EDE.0000000000000559 27749700 PMC5133381

[B8] Carrasco J. Godelaine D. Van Pel A. Boon T. van der Bruggen P. (2006). CD45RA on human CD8 T cells is sensitive to the time elapsed since the last antigenic stimulation. Blood 108 (9), 2897–2905. 10.1182/blood-2005-11-007237 16857986

[B9] Carrer M. Crosby J. R. Sun G. Zhao C. Damle S. S. Kuntz S. G. (2020). Antisense oligonucleotides targeting Jagged 1 reduce house dust mite–induced goblet cell metaplasia in the adult murine lung. Am. J. Respir. Cell Mol. Biol. 63 (1), 46–56. 10.1165/rcmb.2019-0257OC 32176858

[B10] Cesarone M. R. Belcaro G. Hu S. Dugall M. Hosoi M. Ledda A. (2019). Supplementary prevention and management of asthma with quercetin phytosome: a pilot registry. Minerva Med. 110 (6), 524–529. 10.23736/S0026-4806.19.06319-5 31578841

[B11] Chen L.-W. Ko W.-C. (2021). Suppressive effects of rutin, quercitrin, and isoquercitrin on atypical allergic asthma in an animal model. Med. Drug Discov. 12, 100106. 10.1016/j.medidd.2021.100106

[B12] Chen S. Lan W. (2025). Composition analysis of seeds of Petroselinum crispum(Mill.)Fuss by UPLC-QE-Orbitrap-MS. Chin. J. Hosp. 45 (04), 410–417. 10.13286/j.1001-5213.2025.04.07

[B13] Chen C.-Y. Wu K.-H. Guo B.-C. Lin W.-Y. Chang Y.-J. Wei C.-W. (2023). Personalized medicine in severe asthma: from biomarkers to biologics. Int. J. Mol. Sci. 25 (1), 182. 10.3390/ijms25010182 38203353 PMC10778979

[B14] Chen Z. Yu J. Wang H. Xu P. Fan L. Sun F. (2024). Flexible scaffold-based cheminformatics approach for polypharmacological drug design. Cell 187 (9), 2194–2208.e2122. 10.1016/j.cell.2024.02.034 38552625

[B15] Cosío B. G. Mann B. Ito K. Jazrawi E. Barnes P. J. Chung K. F. (2004). Histone acetylase and deacetylase activity in alveolar macrophages and blood mononocytes in asthma. Am. J. Respir. Crit. Care Med. 170 (2), 141–147. 10.1164/rccm.200305-659OC 15087294

[B16] Davey Smith G. Holmes M. V. Davies N. M. Ebrahim S. (2020). Mendel’s laws, Mendelian randomization and causal inference in observational data: substantive and nomenclatural issues. Eur. J. Epidemiol. 35 (2), 99–111. 10.1007/s10654-020-00622-7 32207040 PMC7125255

[B17] Deshmukh A. P. Vasaikar S. V. Tomczak K. Tripathi S. Den Hollander P. Arslan E. (2021). Identification of EMT signaling cross-talk and gene regulatory networks by single-cell RNA sequencing. Proc. Natl. Acad. Sci. 118 (19), e2102050118. 10.1073/pnas.2102050118 33941680 PMC8126782

[B18] Feng G. He N. Gao J. Li X. Zhang F. Liu C. (2024). Causal relationship between key genes and metabolic dysfunction‐associated fatty liver disease risk mediated by immune cells: a Mendelian randomization and mediation analysis. Diabetes Obes. Metab. 26, 5590–5599. 10.1111/dom.15925 39228284

[B19] Gul S. Zhang C. (2025). Single cell RNA sequencing and its impact on understanding human embryo development. Diabetes Obes. Metab. 26 (16), 7741. 10.3390/ijms26167741 40869062 PMC12386559

[B20] He C. Chen J. Liu J. Li Y. Zhou Y. Mao T. (2022). Geranium wilfordii maxim.: a review of its traditional uses, phytochemistry, pharmacology, quality control and toxicology. J. Ethnopharmacol. 285, 114907. 10.1016/j.jep.2021.114907 34896206

[B21] He S. Chen Y. W. Ye J. Wang Y. Chen Q. K. Liu S. Y. (2025). Investigating the metabolic reprogramming mechanisms in diabetic nephropathy: a comprehensive analysis using bioinformatics and machine learning. Front. Cell Dev. Biol. 13, 1630708. 10.3389/fcell.2025.1630708 40950408 PMC12426288

[B22] Hemani G. Tilling K. Davey Smith G. (2017). Orienting the causal relationship between imprecisely measured traits using GWAS summary data. PLOS Genet. 13 (11), e1007081. 10.1371/journal.pgen.1007081 29149188 PMC5711033

[B23] Hoffmann P. Eder R. Boeld T. J. Doser K. Piseshka B. Andreesen R. (2006). Only the CD45RA+ subpopulation of CD4+ CD25high T cells gives rise to homogeneous regulatory T-cell lines upon in vitro expansion. Blood 108 (13), 4260–4267. 10.1182/blood-2006-06-027409 16917003

[B24] Hozumi K. Negishi N. Suzuki D. Abe N. Sotomaru Y. Tamaoki N. (2004). Delta-like 1 is necessary for the generation of marginal zone B cells but not T cells in vivo. Nat. Immunol. 5 (6), 638–644. 10.1038/ni1075 15146182

[B25] Huang Y. Qiu C. (2022). Research advances in airway remodeling in asthma: a narrative review. Ann. Transl. Med. 10 (18), 1023. 10.21037/atm-22-2835 36267708 PMC9577744

[B26] Huang M. Yao P.-W. Chang M.D.-T. Ng S.-K. Yu C.-H. Zhang Y.-f. (2015). Identification of anti-inflammatory fractions of Geranium wilfordii using tumor necrosis factor-alpha as a drug target on Herbochip®–an array-based high throughput screening platform. BMC Complement. Altern. Med. 15 (1), 146. 10.1186/s12906-015-0665-9 25963543 PMC4443519

[B27] Huang M. Wei Y. Dong J. (2020). Epimedin C modulates the balance between Th9 cells and Treg cells through negative regulation of noncanonical NF-κB pathway and MAPKs activation to inhibit airway inflammation in the ovalbumin–induced murine asthma model. Pulm. Pharmacol. Ther. 65, 102005. 10.1016/j.pupt.2021.102005 33636365

[B28] Huang N. Chi H. Qiao J. (2020). Role of regulatory T cells in regulating fetal-maternal immune tolerance in healthy pregnancies and reproductive diseases. Front. Immunol. 11, 1023. 10.3389/fimmu.2020.01023 32676072 PMC7333773

[B29] Jesenak M. Zelieskova M. Babusikova E. (2017). Oxidative stress and bronchial asthma in children—causes or consequences? Front. Pediatr. 5, 162. 10.3389/fped.2017.00162 28791280 PMC5523023

[B30] Kawasaki T. Ikegawa M. Kawai T. (2022). Antigen presentation in the lung. Front. Immunol. 13, 860915. 10.3389/fimmu.2022.860915 35615351 PMC9124800

[B31] Kim S.-H. Jun C.-D. Suk K. Choi B.-J. Lim H. Park S. (2006). Gallic acid inhibits histamine release and pro-inflammatory cytokine production in mast cells. Toxicol. Sci. 91 (1), 123–131. 10.1093/toxsci/kfj063 16322071

[B32] Lalman C. Stabler K. R. Yang Y. Walker J. L. (2025). Supervised machine-based learning and computational analysis to reveal unique molecular signatures associated with wound healing and fibrotic outcomes to lens injury. Int. J. Mol. Sci. 26 (15), 7422. 10.3390/ijms26157422 40806551 PMC12347510

[B33] Lan M. (1959). dian nan ben cao. Available online at: https://agris.fao.org/search/en/providers/122376/records/647473002d5d435c424ef976 (Accessed April 5, 2025).

[B34] Lee S. U. Kim M.-O. Kang M.-J. Oh E. S. Ro H. Lee R. W. (2021). Transforming growth factor β inhibits muc5ac expression by smad3/hdac2 complex formation and nf-κb deacetylation at k310 in nci-h292 cells. Mol. Cells 44 (1), 38–49. 10.14348/molcells.2020.0188 33510050 PMC7854180

[B35] León B. Ballesteros-Tato A. (2021). Modulating Th2 cell immunity for the treatment of asthma. Front. Immunol. 12, 637948. 10.3389/fimmu.2021.637948 33643321 PMC7902894

[B36] Li J. (2020). An application study of comprehensive quality evaluation on Magnolia liliflora by near-infrared spectroscopy. Guangdong Pharmaceutical University.

[B37] Li L. Deng X. Wang S. Huang T. (2025a). Integrating traditional omics and machine learning approaches to identify microbial biomarkers and therapeutic targets in pediatric inflammatory bowel disease. Front. Pharmacol. 16, 1545392. 10.3389/fphar.2025.1545392 40297136 PMC12034630

[B38] Li L. Guan Y. Du Y. Chen Z. Xie H. Lu K. (2025b). Exploiting omic-based approaches to decipher Traditional Chinese medicine. J. Ethnopharmacol. 337, 118936. 10.1016/j.jep.2024.118936 39413937

[B39] Lin S. Yang X. (2023). MAPK1 knockdown ameliorated immune and inflammatory abnormalities in a mouse model of refractory asthma. Trop. J. Pharm. Res. 22 (9), 1831–1840. 10.4314/tjpr.v22i9.9

[B40] Liu F. Xuan N.-X. Ying S.-M. Li W. Chen Z.-H. Shen H.-H. (2016). Herbal medicines for asthmatic inflammation: from basic researches to clinical applications. Mediat. Inflamm. 2016 (1), 6943135. 10.1155/2016/6943135 27478309 PMC4958455

[B41] Liu X. Zhang C. Fu Y. Dai J. Lu J. Liu G. (2025). Huangkui capsule combined with finerenone attenuates diabetic nephropathy by regulating the JAK2/STAT3 signaling pathway based on network pharmacology, molecular docking, and experimental verification. Front. Pharmacol. 16, 1625286. 10.3389/fphar.2025.1625286 40832613 PMC12358392

[B42] Ma B. Athari S. S. Mehrabi Nasab E. Zhao L. (2021). PI3K/AKT/mTOR and TLR4/MyD88/NF-κB signaling inhibitors attenuate pathological mechanisms of allergic asthma. Inflammation 44, 1895–1907. 10.1007/s10753-021-01466-3 33860870

[B43] Machura E. Mazur B. Pieniążek W. Karczewska K. (2008). Expression of naive/memory (CD45RA/CD45RO) markers by peripheral blood CD4+ and CD8+ T cells in children with asthma. Arch. Immunol. Ther. Exp. 56 (1), 55–62. 10.1007/s00005-008-0005-6 18250971 PMC2734248

[B44] Mishra V. Banga J. Silveyra P. (2018). Oxidative stress and cellular pathways of asthma and inflammation: therapeutic strategies and pharmacological targets. Pharmacol. Ther. 181, 169–182. 10.1016/j.pharmthera.2017.08.011 28842273 PMC5743757

[B45] Morgan B. W. Grigsby M. R. Siddharthan T. Chowdhury M. Rubinstein A. Gutierrez L. (2019). Epidemiology and risk factors of asthma-chronic obstructive pulmonary disease overlap in low-and middle-income countries. J. Allergy Clin. Immunol. 143 (4), 1598–1606. 10.1016/j.jaci.2018.06.052 30291842 PMC7079232

[B46] Mortimer K. Reddel H. K. Pitrez P. M. Bateman E. D. (2022). Asthma management in low and middle income countries: case for change. Eur. Respir. J. 60 (3), 2103179. 10.1183/13993003.03179-2021 35210321 PMC9474897

[B47] Mukherjee M. Svenningsen S. Nair P. (2017). Glucocortiosteroid subsensitivity and asthma severity. Curr. Opin. Pulm. Med. 23 (1), 78–88. 10.1097/MCP.0000000000000337 27801710

[B48] Nur Husna S. M. Md Shukri N. Mohd Ashari N. S. Wong K. K. (2022). IL-4/IL-13 axis as therapeutic targets in allergic rhinitis and asthma. PeerJ 10, e13444. 10.7717/peerj.13444 35663523 PMC9161813

[B49] Orrù V. Steri M. Sidore C. Marongiu M. Serra V. Olla S. (2020). Complex genetic signatures in immune cells underlie autoimmunity and inform therapy. Nat. Genet. 52 (10), 1036–1045. 10.1038/s41588-020-0684-4 32929287 PMC8517961

[B50] Panek M. Jonakowski M. Zioło J. Wieteska Ł. Małachowska B. Pietras T. (2016). A novel approach to understanding the role of polymorphic forms of the NR3C1 and TGF-β1 genes in the modulation of the expression of IL-5 and IL-15 mRNA in asthmatic inflammation. Clin. Transl. Allergy 13 (6), 4879–4887. 10.1002/clt2.12172 27081784

[B51] Panek M. G. Karbownik M. S. Górski K. M. Koćwin M. Kardas G. Marynowski M. (2022). New insights into the regulation of TGF‐β/Smad and MPK signaling pathway gene expressions by nasal allergen and methacholine challenge test in asthma. Mol. Med. Rep. 12 (7), e12172. 10.3892/mmr.2016.5104 35800124 PMC9250282

[B52] Pishesha N. Harmand T. J. Ploegh H. L. (2022). A guide to antigen processing and presentation. Nat. Rev. Immunol. 22 (12), 751–764. 10.1038/s41577-022-00707-2 35418563

[B53] Potaczek D. P. Bazan-Socha S. Wypasek E. Wygrecka M. Garn H. (2024). Recent developments in the role of histone acetylation in asthma. Int. Arch. Allergy Immunol. 185 (7), 641–651. 10.1159/000536460 38522416

[B54] Quan J. Peng J. Sun J. Fan B. Li Z. Wang X. (2025). Rapid identification of components in houyanqing oral liquid by UHPLC Q-Exactive orbitrap MS and molecular networking. Mod. Chin. Med. 27 (03), 472–487. 10.13313/j.issn.1673-4890.20240717003

[B55] Rajvanshi N. Kumar P. Goyal J. P. (2024). Global initiative for asthma guidelines 2024: an update. Indian Pediatr. 61, 781–786. 10.1007/s13312-024-3260-7 39051318

[B56] Sahoo A. Wali S. Nurieva R. (2016). T helper 2 and T follicular helper cells: regulation and function of interleukin-4. Cytokine Growth Factor Rev. 30, 29–37. 10.1016/j.cytogfr.2016.03.011 27072069 PMC5110032

[B57] Saito T. Chiba S. Ichikawa M. Kunisato A. Asai T. Shimizu K. (2003). Notch2 is preferentially expressed in mature B cells and indispensable for marginal zone B lineage development. Immunity 18 (5), 675–685. 10.1016/S1074-7613(03)00111-0 12753744

[B58] Shang W. Wang G. Wang Y. Han D. (2022). The safety of long-term use of inhaled corticosteroids in patients with asthma: a systematic review and meta-analysis. Clin. Immunol. 236, 108960. 10.1016/j.clim.2022.108960 35218965

[B59] Shrestha Palikhe N. Wu Y. Konrad E. Gandhi V. D. Rowe B. H. Vliagoftis H. (2021). Th2 cell markers in peripheral blood increase during an acute asthma exacerbation. Allergy 76 (1), 281–290. 10.1111/all.14543 32750154

[B60] Singh D. Oosterholt S. Pavord I. Garcia G. Abhijith P. Della Pasqua O. (2023). Understanding the clinical implications of individual patient characteristics and treatment choice on the risk of exacerbation in asthma patients with moderate–severe symptoms. Adv. Ther. 40 (10), 4606–4625. 10.1007/s12325-023-02590-2 37589831 PMC10499702

[B61] Thomas D. McDonald V. M. Pavord I. D. Gibson P. G. (2022). Asthma remission: what is it and how can it be achieved? Eur. Respir. J. 60 (5), 2102583. 10.1183/13993003.02583-2021 35361633 PMC9630609

[B62] Timperi E. Barnaba V. (2021). CD39 regulation and functions in T cells. Int. J. Mol. Sci. 22 (15), 8068. 10.3390/ijms22158068 34360833 PMC8348030

[B63] Tindemans I. Vroman H. Lukkes M. van Nimwegen M. de Bruijn M. J. Li B. W. (2019). Increased surface expression of NOTCH on memory T cells in peripheral blood from patients with asthma. J. Allergy Clin. Immunol. 143 (2), 769–771.e763. 10.1016/j.jaci.2018.09.012 30296524

[B64] Tindemans I. van Schoonhoven A. KleinJan A. de Bruijn M. J. Lukkes M. van Nimwegen M. (2020). Notch signaling licenses allergic airway inflammation by promoting Th2 cell lymph node egress. J. Clin. Invest. 130 (7), 3576–3591. 10.1172/JCI128310 32255764 PMC7324208

[B65] Tong S. Yin Y. Bao Y. (2022). Climatotherapy for asthma: research progress and prospect. Environ. Res. 214, 113988. 10.1016/j.envres.2022.113988 35964665

[B66] Vincenzo S. D. Ferrante G. Ferraro M. Cascio C. Malizia V. Licari A. (2023). Oxidative stress, environmental pollution, and lifestyle as determinants of asthma in children. Biology 12 (1), 133. 10.3390/biology12010133 36671825 PMC9856068

[B67] Walter H. Sadeque-Iqbal F. Ulysse R. Castillo D. Fitzpatrick A. Singleton J. (2015). The effectiveness of school-based family asthma educational programs on the quality of life and number of asthma exacerbations of children aged five to 18 years diagnosed with asthma: a systematic review protocol. JBI Evid. Synth. 13 (10), 69–81. 10.11124/jbisrir-2015-2335 26571284

[B68] Wang S. Zhu H. Pan L. Zhang M. Wan X. Xu H. (2023). Systemic inflammatory regulators and risk of acute-on-chronic liver failure: a bidirectional mendelian-randomization study. Front. Cell Dev. Biol. 11, 1125233. 10.3389/fcell.2023.1125233 36743413 PMC9892464

[B69] Wang J. Yan K. Ma L. Yan X. Meng Z. Li J.-a. (2025). Notch signaling pathway interfering as a possible asthma treatment: a narrative review. J. Asthma Allergy 18, 437–446. 10.2147/JAA.S504925 40129884 PMC11932036

[B70] Wang Y. Fan Y. Li M. Song Z. Wang P. Xie F. (2025). Cornus officinalis protects against steroid-induced osteonecrosis of the femoral head through inhibiting inflammatory responses and apoptosis *via* network pharmacology and experimental validation. DDDT 19, 5871–5898. 10.2147/DDDT.S518115 40661830 PMC12256781

[B71] Wills-Karp M. Finkelman F. D. (2008). Untangling the complex web of IL-4–and IL-13–mediated signaling pathways. Sci. Signal. 1 (51), pe55. 10.1126/scisignal.1.51.pe55 19109238 PMC4446705

[B72] Xia T. Ma J. Sun Y. Sun Y. (2022). Androgen receptor suppresses inflammatory response of airway epithelial cells in allergic asthma through MAPK1 and MAPK14. Hum. Exp. Toxicol. 41, 09603271221121320. 10.1177/09603271221121320 35982617

[B73] Xing Y. Zhang J. (2012). The clinical applications of Geranium wilfordii maxim. in Pediatrics., (Chinese Society of pediatrics). Available online at: https://kns.cnki.net/KCMS/detail/detail.aspx?dbcode=CPFD&dbname=CPFD0914&filename=ZHZY201209001250&v (Accessed November 18, 2025).

[B74] Xu J. Yu Z. Li W. (2023). Kaempferol inhibits airway inflammation induced by allergic asthma through NOX4-Mediated autophagy. Hum. Exp. Toxicol. 42, 09603271231154227. 10.1177/09603271231154227 36803065

[B75] Yaagoubi O. M. E. Oularbi L. Bouyahya A. Samaki H. Antri S. E. Aboudkhil S. (2021). The role of the ubiquitin-proteasome pathway in skin cancer development: 26S proteasome-activated NF-κB signal transduction. Cancer Biol. Ther. 10.1080/15384047.2021.1978785 PMC8726620 34583610

[B76] Yamanishi Y. Miyake K. Iki M. Tsutsui H. Karasuyama H. (2017). Recent advances in understanding basophil‐mediated Th2 immune responses. Immunol. Rev. 278 (1), 237–245. 10.1111/imr.12548 28658549

[B77] Yang B. C. Castells M. (2024). Medical algorithm: diagnosis and treatment of drug hypersensitivity reactions to biologicals, 2024 update. Allergy 75 (12), 1534–1539. 10.1111/all.16353 39400368

[B78] Yang S. Huang H. Ji J. (2011). Synthesis of 5,6-Epoxy-β-ionol. Chem. Bull. 74 (03), 270–274. 10.14159/j.cnki.0441-3776.2011.03.013

[B79] Yang G. Zhao Z. Zhang X. Wu A. Huang Y. Miao Y. (2017). Effect of berberine on the renal tubular epithelial-to-mesenchymal transition by inhibition of the Notch/snail pathway in diabetic nephropathy model KKAy mice. Drug Des. Dev. Ther. 11, 1065–1079. 10.2147/DDDT.S124971 28408805 PMC5384688

[B80] Ying H. Kong W. Xu X. (2025). Integrated network pharmacology, machine learning and experimental validation to identify the key targets and compounds of TiaoShenGongJian for the treatment of breast cancer. Oncotargets Ther. 18, 49–71. 10.2147/OTT.S486300 39835272 PMC11745062

[B81] Yip W. Hughes M. R. Li Y. Cait A. Hirst M. Mohn W. W. (2021). Butyrate shapes immune cell fate and function in allergic asthma. Front. Immunol. 12, 628453. 10.3389/fimmu.2021.628453 33659009 PMC7917140

[B82] Yuan L. Tao J. Wang J. She W. Zou Y. Li R. (2025). Global, regional, national burden of asthma from 1990 to 2021, with projections of incidence to 2050: a systematic analysis of the global burden of disease study 2021. EClinicalMedicine 80, 103051. 10.1016/j.eclinm.2024.103051 39867965 PMC11764843

[B83] Yuan Y. Yu L. Bi C. Huang L. Su B. Nie J. (2025). A new paradigm for drug discovery in the treatment of complex diseases: drug discovery and optimization. Chin. Med. 20 (1), 40. 10.1186/s13020-025-01075-4 40122800 PMC11931805

[B84] Zeng K. Zhou X. Nie C. Zhang Y. (2023). A serum pharmacochemical study on the pharmacodynamic substances basis of the Anti- ischemic injury of Buyang Huanwu decoction. Tradit. Chin. Drug Res. Clin. Pharmacol. 34 (09), 1227–1235. 10.19378/j.issn.1003-9783.2023.09.008

[B85] Zhang J. Zou Y. Chen L. Xu Q. Wang Y. Xie M. (2022). Regulatory T cells, a viable target against airway allergic inflammatory responses in asthma. Front. Immunol. 13, 902318. 10.3389/fimmu.2022.902318 35757774 PMC9226301

[B86] Zhang X. Qiao H. Li M. Bai G. (2023). Effects of simvastatin on immunoreaction and inflammation in rats with asthma by regulating NOTCH signaling pathway. Cell. Mol. Biol. 69, 230–234. 10.14715/cmb/2023.69.15.39 38279436

[B87] Zhang B. Feng X. Tian L. Xiao B. Hou L. Mo B. (2025). Epithelial-mesenchymal transition in asthma: its role and underlying regulatory mechanisms. Front. Immunol. 16, 1519998. 10.3389/fimmu.2025.1519998 39911398 PMC11794105

