# Supplementary material for: Multifaceted mechanistic exploration of Geranium wilfordii Maxim. in asthma treatment: integrating network pharmacology, machine learning, Mendelian randomization and experimental validation
Source: Front Cell Dev Biol. 2026 Mar 25;14:1761424. doi: 10.3389/fcell.2026.1761424 (PMC13056865; doi:10.3389/fcell.2026.1761424)
Supplement: Supplementary file 1 [file DataSheet1.docx]

Supplementary Material

**Table S1.** Elution gradient table of liquid chromatography

| Time (min) | Mobile phase A (%) | Mobile phase B (%) |
| --- | --- | --- |
| 0 | 95 | 5 |
| 2 | 95 | 5 |
| 4 | 70 | 30 |
| 8 | 50 | 50 |
| 10 | 20 | 80 |
| 12 | 0 | 100 |

**Table S2.** The primer sequences of each gene

| Genes | Primers |
| --- | --- |
| β-actin | Forward primer: GATGGTGGGAATGGGTCAGAAGG |
|  | Reverse primer: TTGTAGAAGGTGTGGTGCCAGATC |
| NOTCH2 | Forward primer: GACATCCGCCACACTCCATCC |
|  | Reverse primer: AGTCCTCAGCATCTTCGTCTTCATC |
| HDAC2 | Forward primer: CTGGGGACAGGCTTGGTTGTTTC |
|  | Reverse primer: CCTCCACCGAGCATCAGCAATG |
| MAPK1 | Forward primer: GCCTTCCAACCTCCTGCTGAAC |
|  | Reverse primer: GCTCTGTACCAACGTGTGGCTAC |

**Table S3.** Summary of key targets identified by multiple machine learning algorithms and PPI network analysis

| Model | Genes | n |
| --- | --- | --- |
| RF | ESRRA, CD81, GSR, GNPAT, NFE2L2, CTSD, MAPK1, NCOR2, RAC1, HDAC2, PRKDC, HSP90AA1, OAT, EP300, IRAK1, NOTCH2, COMT, RELA, RPS6KA1, HRAS | 20 |
| PPI | BCL2L1, CD81, CTSB, CTSD, EIF2AK3, EP300, GSR, GSTP1, HDAC2, HRAS, HSP90AA1, HSP90AB1, HSPA1A, HSPA1B, HSPA8, IRAK1, KEAP1, MAPK1, MDM2, NFE2L2, NOTCH2, PARP1, PGD, PPIA, PRKCD, PRKDC, RELA, RPS6KA1, SREBF2, STAT3 | 30 |
| SVM-RFE | CTSD, HDAC2, ESRRA, RPS6KA1, MDM2, GNPAT, NOTCH2, COMT, IGFBP4, NFE2L2, MAPK1, MIF, PRKCD, RAC1, ERAP1, GSR, CD81, PGD | 18 |
| LASSO | NFE2L2, CD81, CTSD, HDAC2, GNPAT, PRKCD, ESRRA, RPS6KA1, GSR, MAPK1, RAC1, COMT, NOTCH2, NR1H2 | 14 |
| XGBoost | ESRRA, GNPAT, CTSD, COMT, CD81, NFE2L2, HDAC2, NOTCH2, IGFBP4, MAPKAPK2, RAC1, EP300, MAPK1, RELA | 14 |
| Boruta | IRAK1, NFE2L2, CD81, CTSD, OAT, RELA, HDAC2, GNPAT, EP300, KEAP1, ESRRA, GSR, BCL2L1, NCOR2, MAPK1, RAC1, PRKDC, COMT, HSP90AA1, NOTCH2, HRAS, APH1B | 22 |

**Table S4.** BALF inflammatory cell counts

| Group | WBC (10^4^) | LYM (10^4^) | NEU (10^4^) | EOS (10^4^) | MON (10^4^) |
| --- | --- | --- | --- | --- | --- |
| Control | 81.181±24.967 | 42.517±12.757 | 30.593±5.885 | 4.059±1.248 | 2.595±0.643 |
| OVA | 275.000±53.104^##^ | 179.770±53.852^##^ | 83.626±22.620^#^ | 46.750±9.028^##^ | 11.574±3.514^#^ |
| DEX | 86.667±25.033^**^ | 53.735±19.438^**^ | 23.422±8.010^**^ | 4.420±1.277^**^ | 3.343±1.134^*^ |
| GWM_L | 176.667±21.602^**^ | 100.062±29.009^**^ | 68.968±10.178 | 12.367±1.512^**^ | 7.432±1.323 |
| GWM_M | 113.889±31.157^**^ | 74.290±22.025^**^ | 34.767±10.968^*^ | 7.118±1.947^**^ | 4.832±1.441^*^ |
| GWM_H | 93.125±20.943^**^ | 57.106±19.908^**^ | 32.455±5.270^*^ | 4.889±1.099^**^ | 3.563±1.151^*^ |

Values expressed as mean±SD (*n* = 6). WBC = white blood cells; LYM = lymphocytes; NEU = neutrophils; EOS = eosinophils; MON = monocytes. ^##^*P* < 0.01, ^#^*P* < 0.05 compared with Control group; ^**^*P* < 0.01, ^*^*P* < 0.05 compared with OVA group.

**Table S5.** Cytokine and IgE levels in serum and BALF

| Group | IL-4 (ng/mL) | IL-5 (ng/L) | IL-13 (ng/L) | IgE (U/mL) |
| --- | --- | --- | --- | --- |
| Control | 8.154±2.398 | 8.220±1.693 | 3.289±1.025 | 8.390±1.749 |
| OVA | 24.268±5.058^##^ | 34.397±7.704^##^ | 13.338±3.422^##^ | 14.143±1.486^##^ |
| DEX | 13.779±2.247^**^ | 9.597±2.941^**^ | 3.191±1.082^**^ | 9.878±3.165^**^ |
| GWM_L | 17.866±4.134^**^ | 19.670±3.493^*^ | 7.440±2.071 | 10.210±3.102^**^ |
| GWM_M | 15.977±4.304^**^ | 12.753±2.775^**^ | 3.751±1.178^**^ | 9.305±1.639^**^ |
| GWM_H | 14.118±2.345^**^ | 12.025±3.481^**^ | 3.119±1.073^**^ | 8.815±2.047^**^ |

Values expressed as mean±SD (*n* = 6). ^##^*P* < 0.01, ^#^*P* < 0.05 compared with Control group; ^**^*P* < 0.01, ^*^*P* < 0.05 compared with OVA group.

**Table S6.** mRNA Expression Levels of NOTCH2, HDAC2 and MAPK1

| Group | NOTCH2 | HDAC2 | MAPK1 |
| --- | --- | --- | --- |
| Control | 1.010±0.165 | 1.014±0.202^##^ | 1.003±0.094 |
| OVA | 1.868±0.312^##^ | 0.431±0.075^##^ | 1.665±0.091^##^ |
| GWM_H | 1.342±0.178^*^ | 0.836±0.142^*^ | 1.279±0.052^**^ |

Values expressed as mean±SD (*n* = 6). ^##^*P* < 0.01, ^#^*P* < 0.05 compared with Control group; ^**^*P* < 0.01, ^*^*P* < 0.05 compared with OVA group.

**Table S7.** Mediation effect of hub genes on asthma via immunophenotypes

| Exposure | Mediator | Outcome | Total effect | Direct effect | Mediation effect | Mediation Proportion (95% CI) |
| --- | --- | --- | --- | --- | --- | --- |
| NOTCH2 | CD25^++^ CD45RA^-^ CD4 not regulatory T cell %CD4^+^ T cell | asthma | 0.024 | 0.001 | 0.023 | 5.98%  (-8.74% -20.69%) |
| HDAC2 | CD28 on CD39^+^ secreting CD4 regulatory T cell | asthma | -0.209 | -0.207 | -0.002 | 1.12%  (-3.73%-5.97%) |
| MAPK1 | CD25^++^ CD45RA^-^ CD4 not regulatory T cell %CD4^+^ T cell | asthma | 0.037 | 0.035 | 0.002 | 4.48%  (-7.83%-16.78%) |


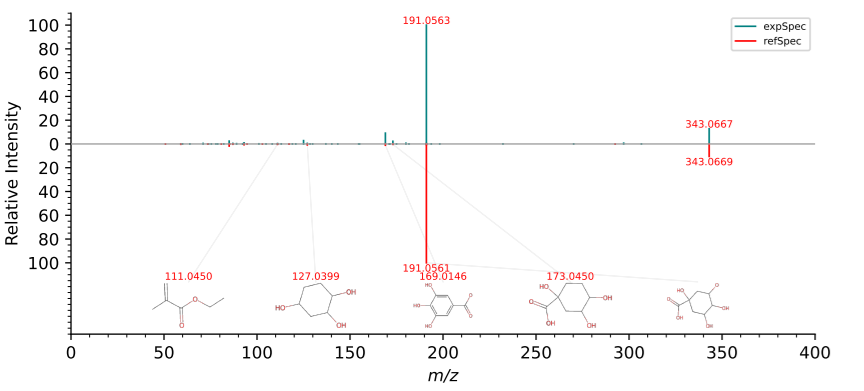


**Figure S1.** The MS/MS spectrum of 3-Galloylquinic acid (compound 1) and its potential fragmentation pathway


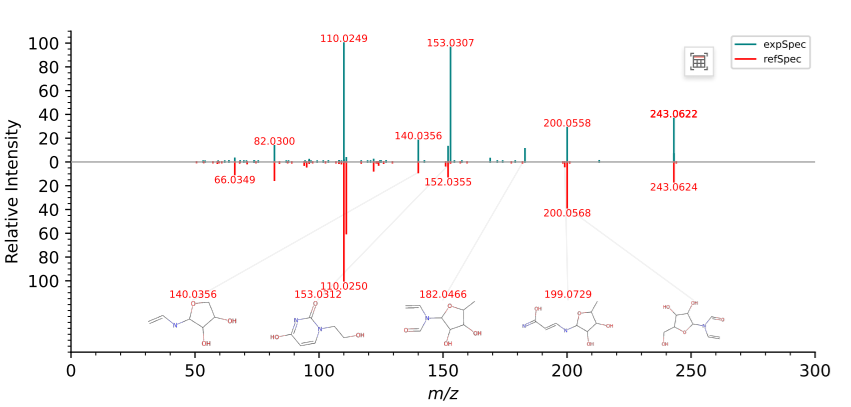


**Figure S2.** The MS/MS spectrum of 1-beta-D-Arabinofuranosyluracil (compound 2) and its potential fragmentation pathway


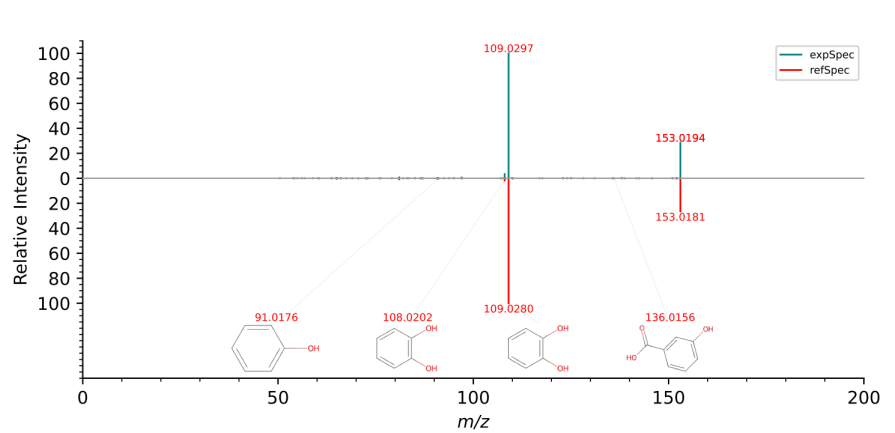


**Figure S3.** The MS/MS spectrum of Protocatechuic acid (compound 4) and its potential fragmentation pathway


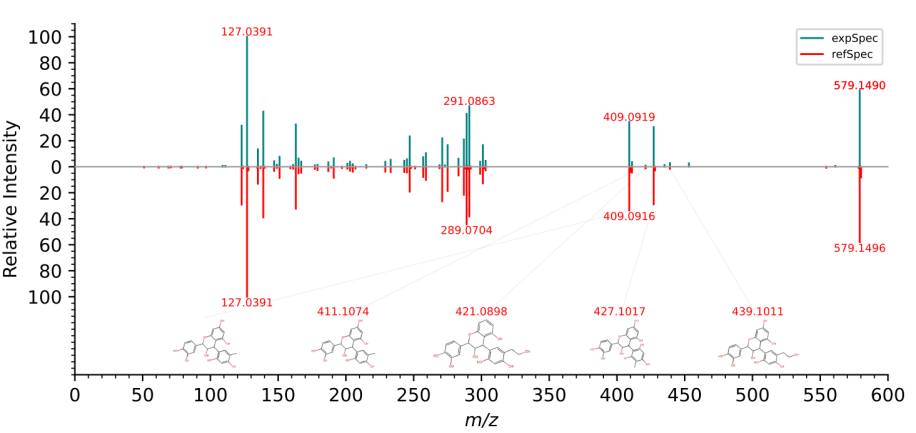


**Figure S4.** The MS/MS spectrum of Procyanidin B1 (compound 6) and its potential fragmentation pathway


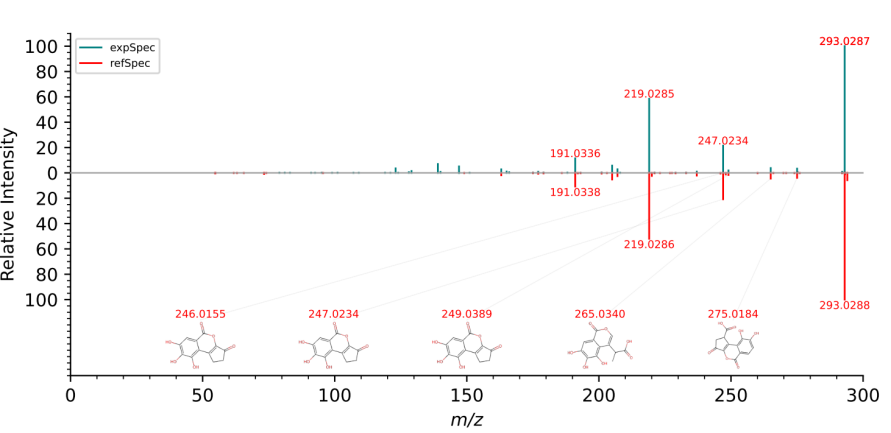


**Figure S5.** The MS/MS spectrum of Brevifolincarboxylic acid (compound 7) and its potential fragmentation pathway


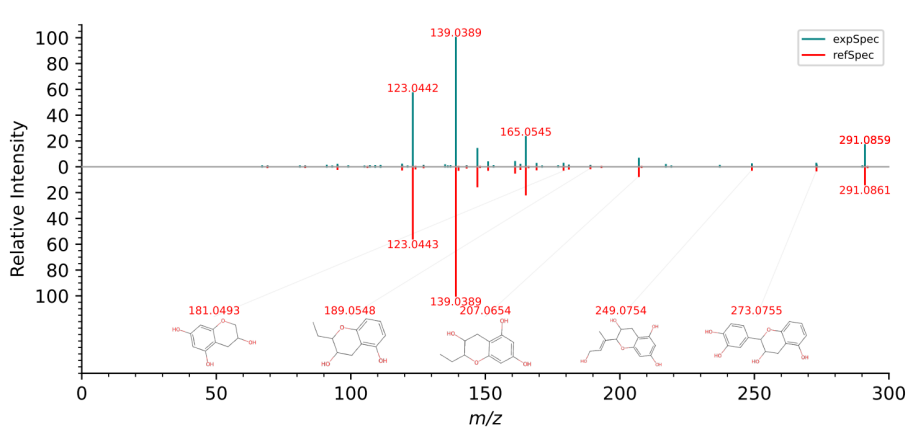


**Figure S6.** The MS/MS spectrum of Epicatechin (compound 9) and its potential fragmentation pathway


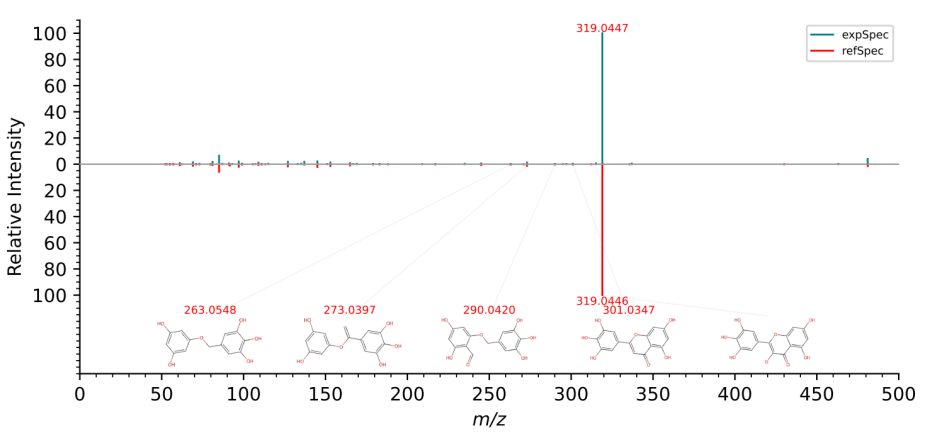


**Figure S7.** The MS/MS spectrum of Myricetin 3-beta-D-glucopyranoside (compound 10) and its potential fragmentation pathway


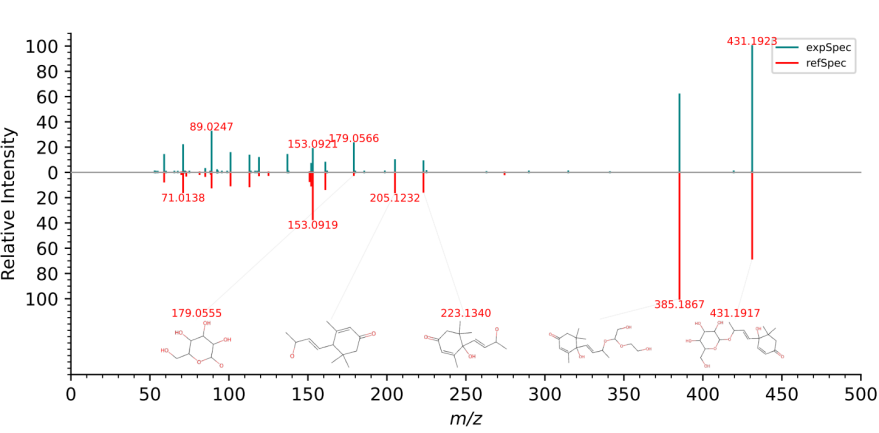


**Figure S8.** The MS/MS spectrum of Roseoside (compound 11) and its potential fragmentation pathway


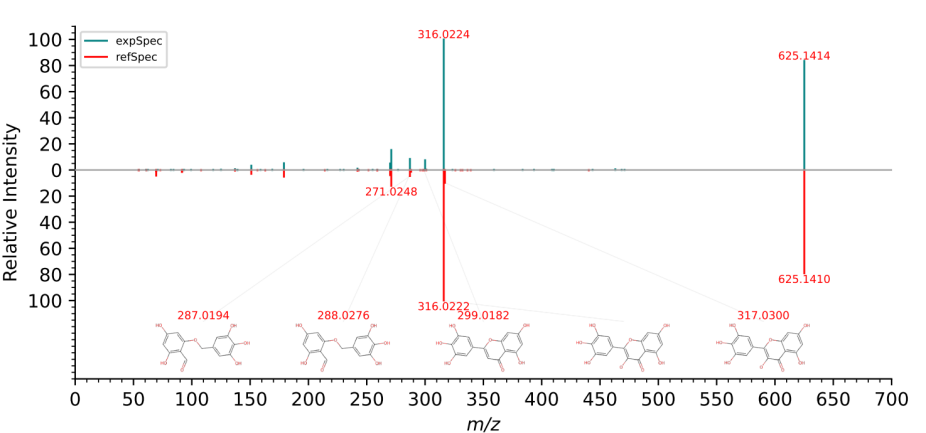


**Figure S9.** The MS/MS spectrum of Myricetin 3-O-rutinoside (compound 13) and its potential fragmentation pathway


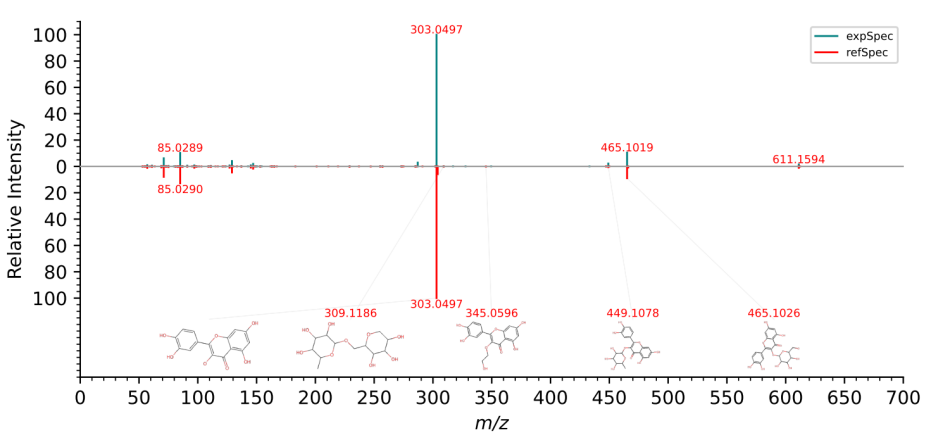


**Figure S10.** The MS/MS spectrum of Rutin (compound 14) and its potential fragmentation pathway


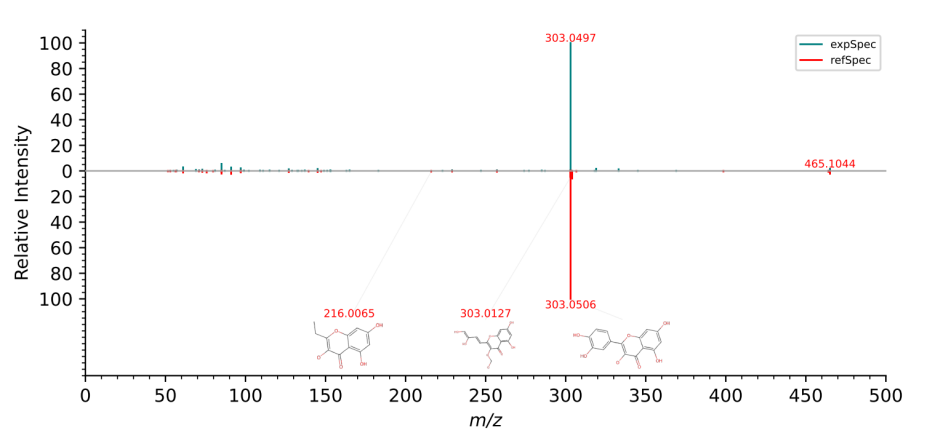


**Figure S11.** The MS/MS spectrum of Hyperoside (compound 15) and its potential fragmentation pathway


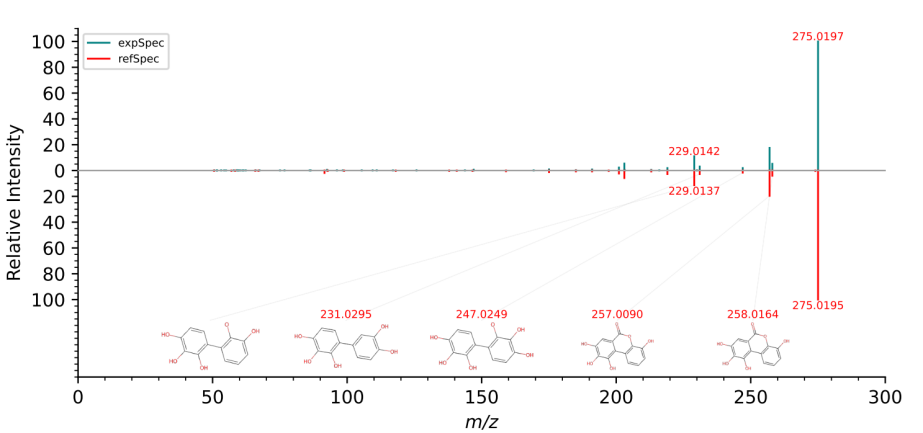


**Figure S12.** The MS/MS spectrum of 3,4,8,9,10-Pentahydroxy Urolithin (compound 16) and its potential fragmentation pathway


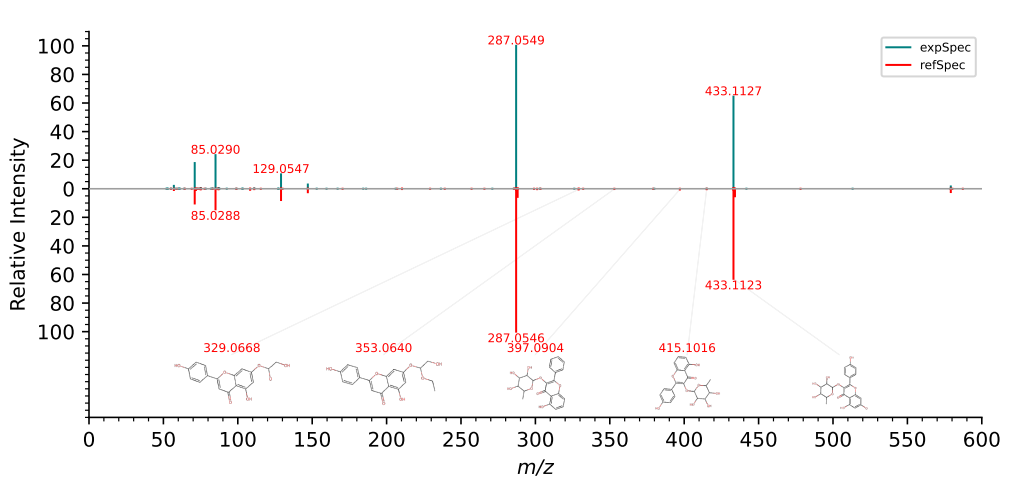


**Figure S13.** The MS/MS spectrum of Kaempferitrin (compound 17) and its potential fragmentation pathway


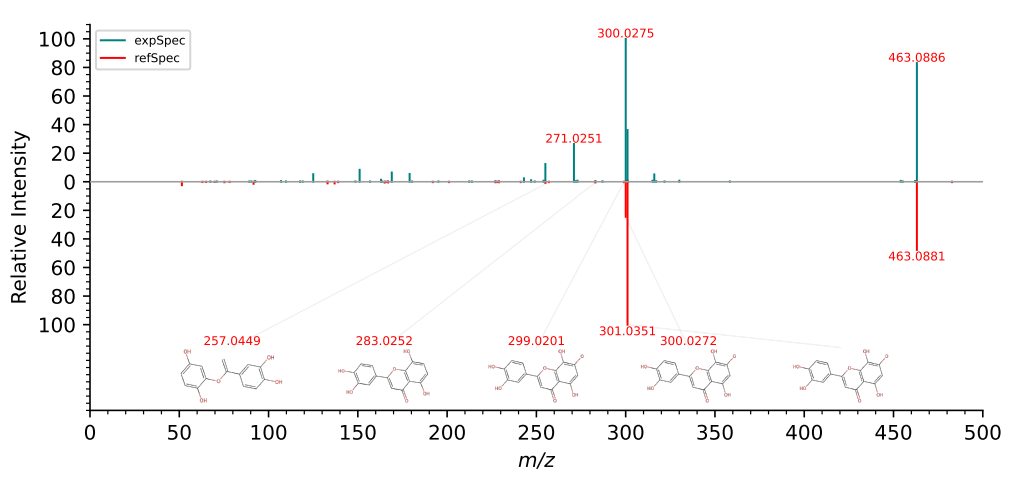


**Figure S14.** The MS/MS spectrum of 7-[(beta-D-Glucopyranosyl)oxy]-3',4',5,8-tetrahydroxyflavone (compound 18) and its potential fragmentation pathway


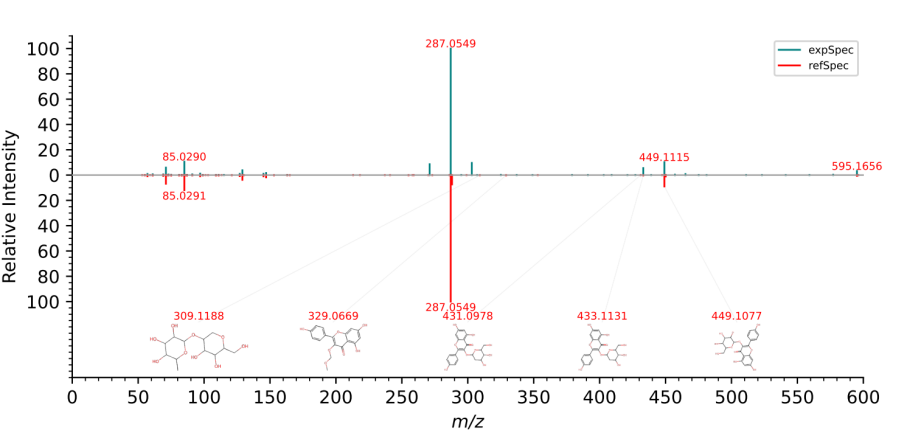


**Figure S15.** The MS/MS spectrum of Kaempferol-3-O-glucorhamnoside (compound 19) and its potential fragmentation pathway


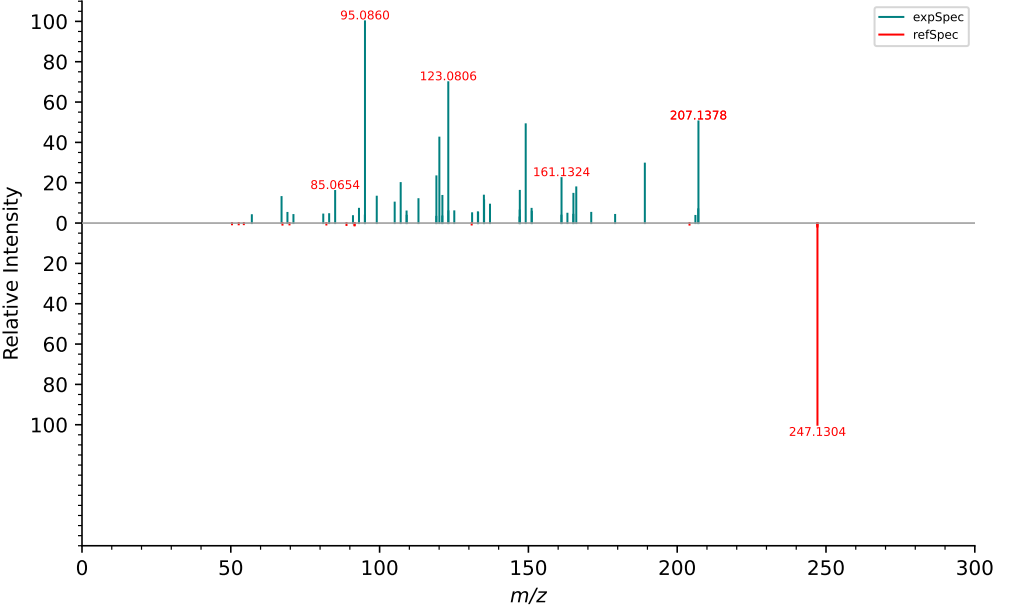


**Figure S16.** The MS/MS spectrum of Vomifoliol (compound 21) and its potential fragmentation pathway


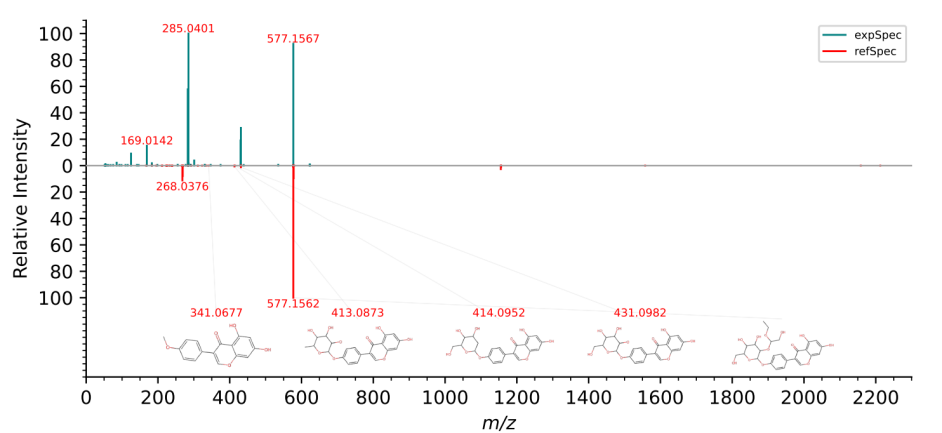


**Figure S17.** The MS/MS spectrum of Sophorabioside (compound 22) and its potential fragmentation pathway


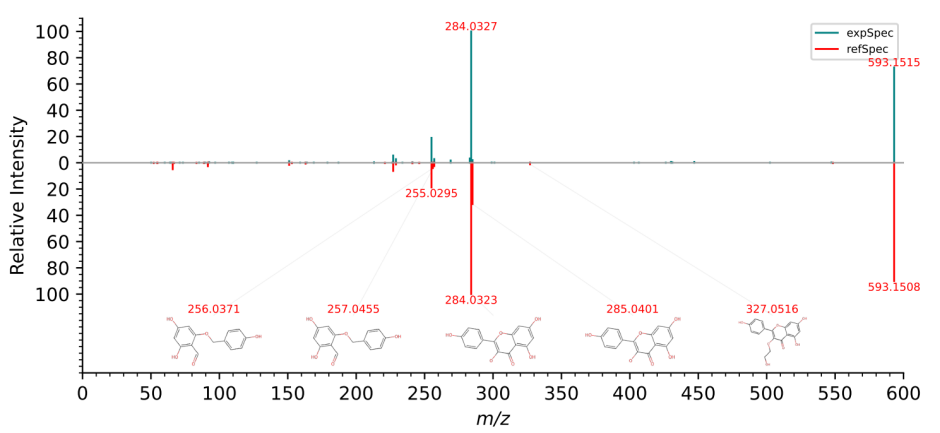


**Figure S18.** The MS/MS spectrum of Biorobin (compound 23) and its potential fragmentation pathway


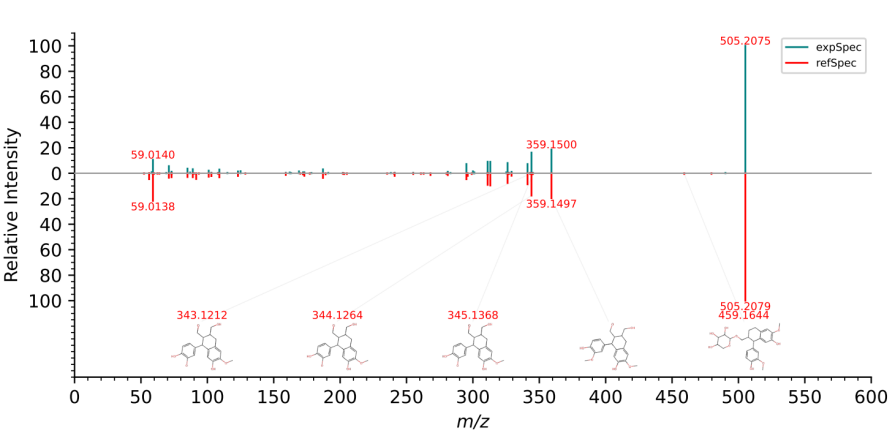


**Figure S19.** The MS/MS spectrum of Aviculin (compound 24) and its potential fragmentation pathway


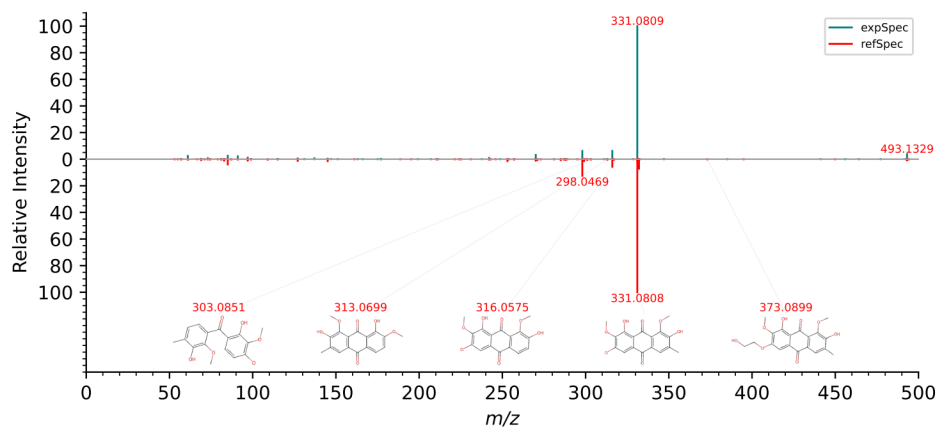


**Figure S20.** The MS/MS spectrum of Aurantio-obtusin beta-D-glucoside (compound 25) and its potential fragmentation pathway


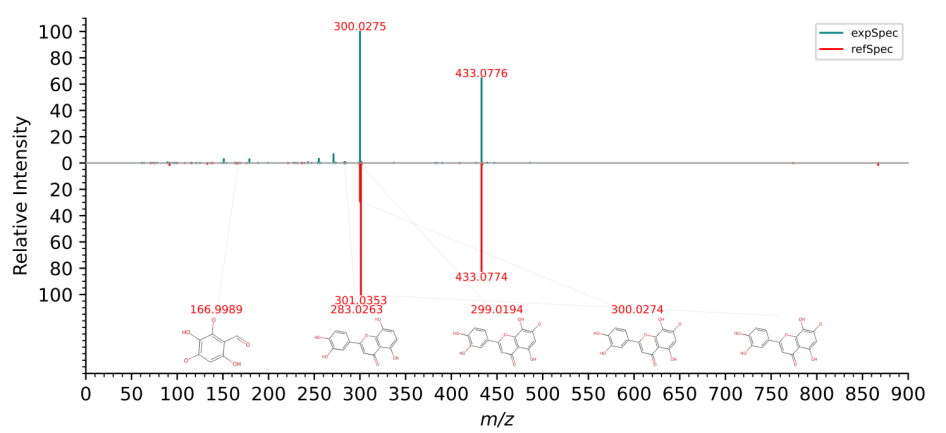


**Figure S21.** The MS/MS spectrum of Hypoletin-7-O-beta-D-Xylopyranoside (compound 26) and its potential fragmentation pathway


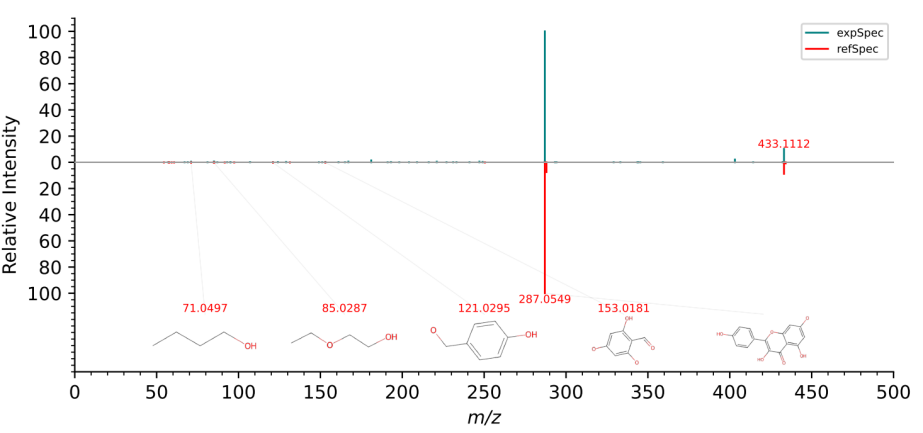


**Figure S22.** The MS/MS spectrum of Kaempferol-7-O-rhamnoside (compound 27) and its potential fragmentation pathway


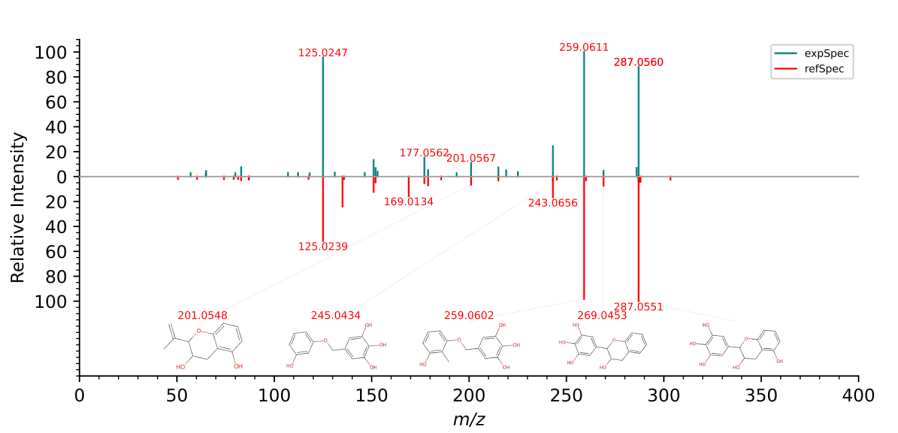


**Figure S23.** The MS/MS spectrum of Gallocatechin (compound 28) and its potential fragmentation pathway


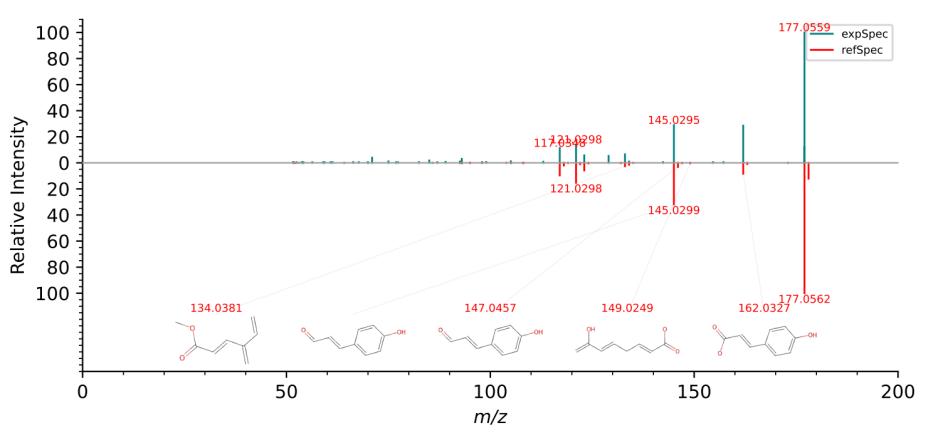


**Figure S24.** The MS/MS spectrum of Methyl p-coumarate (compound 29) and its potential fragmentation pathway


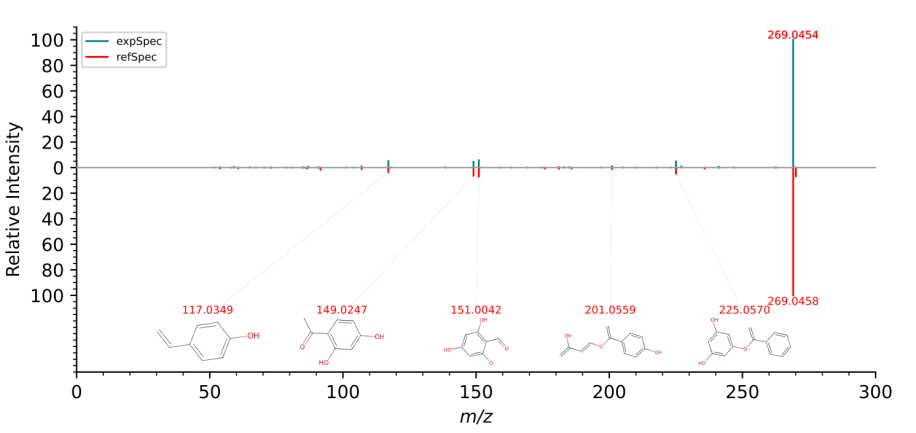


**Figure S25.** The MS/MS spectrum of Apigenin (compound 30) an0 its potential fragmentation pathway


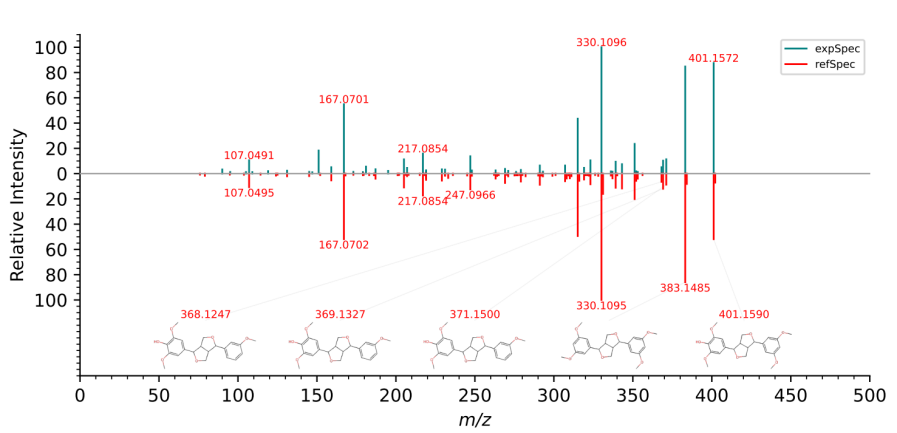


**Figure S26.** The MS/MS spectrum of Syringaresinol (compound 31) and its potential fragmentation pathway


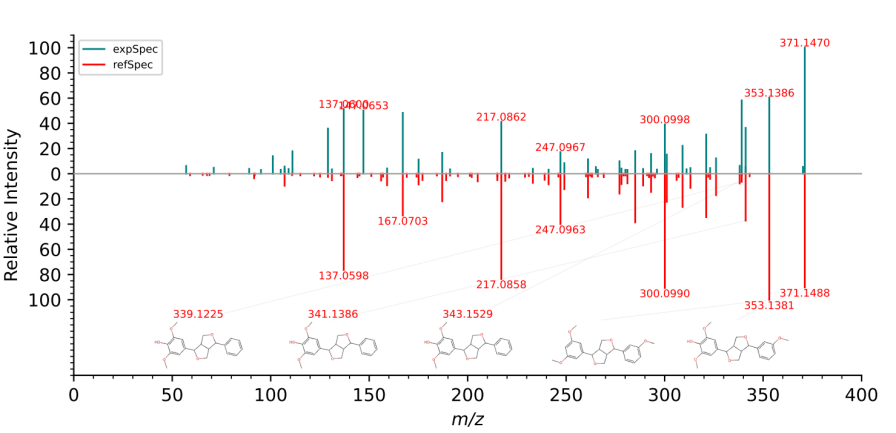


**Figure S27.** The MS/MS spectrum of Medioresil (compound 32) and its potential fragmentation pathway


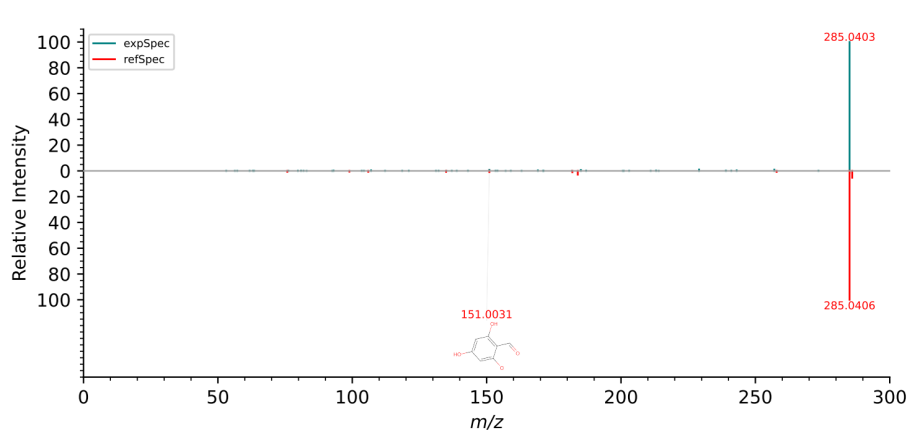


**Figure S28.** The MS/MS spectrum of Kaempferol (compound 33) and its potential fragmentation pathway


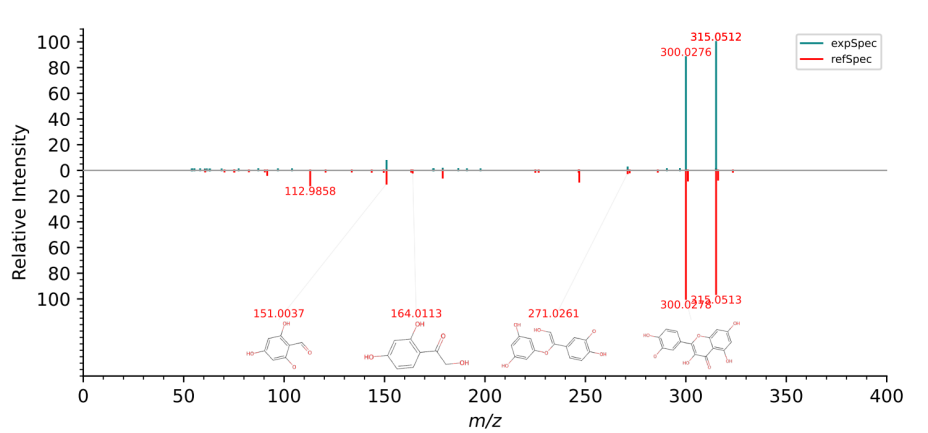


**Figure S29.** The MS/MS spectrum of Isorhamnetin (compound 34) and its potential fragmentation pathway


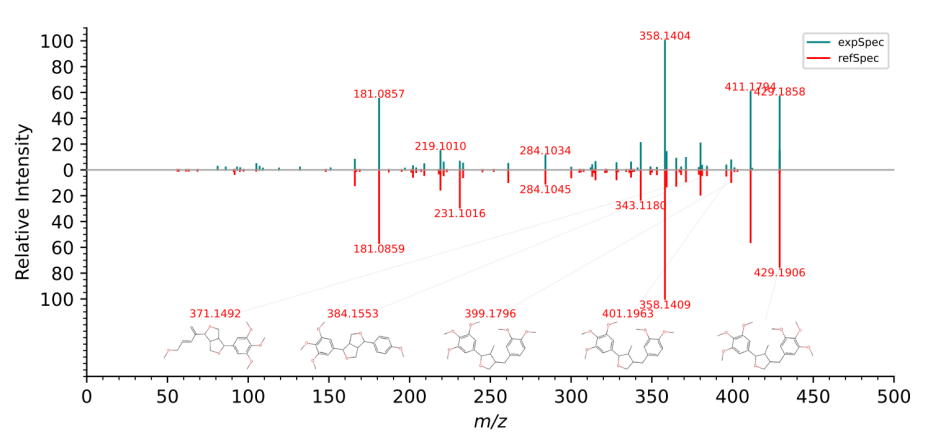


**Figure S30.** The MS/MS spectrum of Yangambin (compound 37) and its potential fragmentation pathway


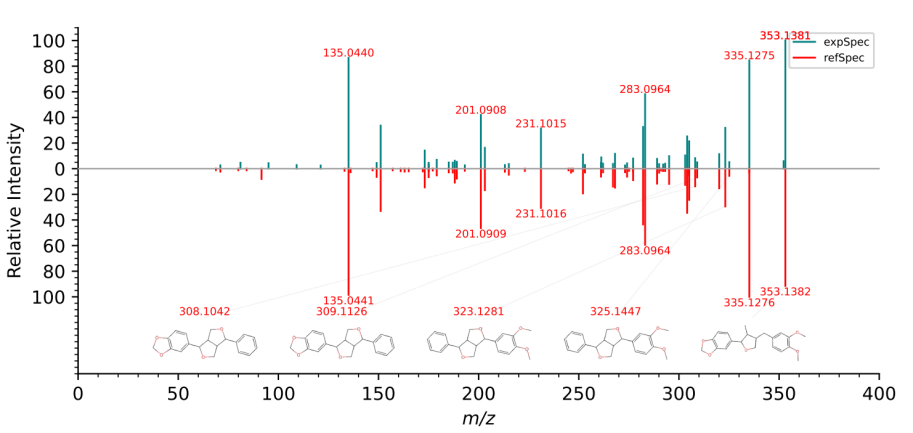


**Figure S31.** The MS/MS spectrum of Kobusin (compound 38) and its potential fragmentation pathway


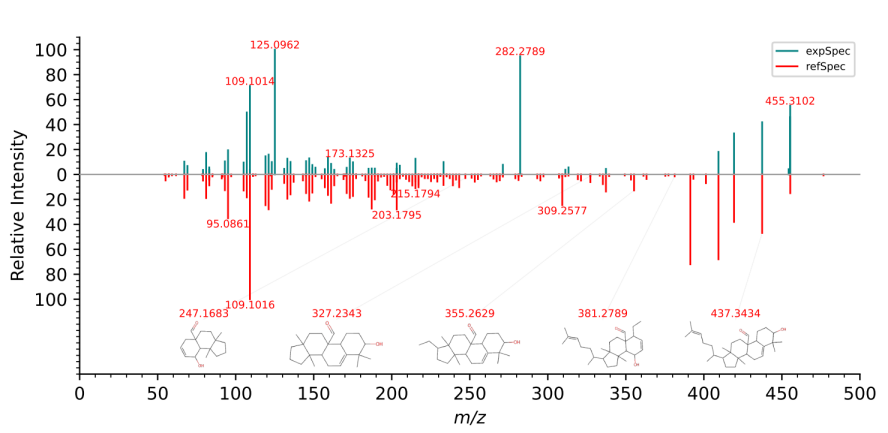


**Figure S32.** The MS/MS spectrum of Momordicine I (compound 39) and its potential fragmentation pathway


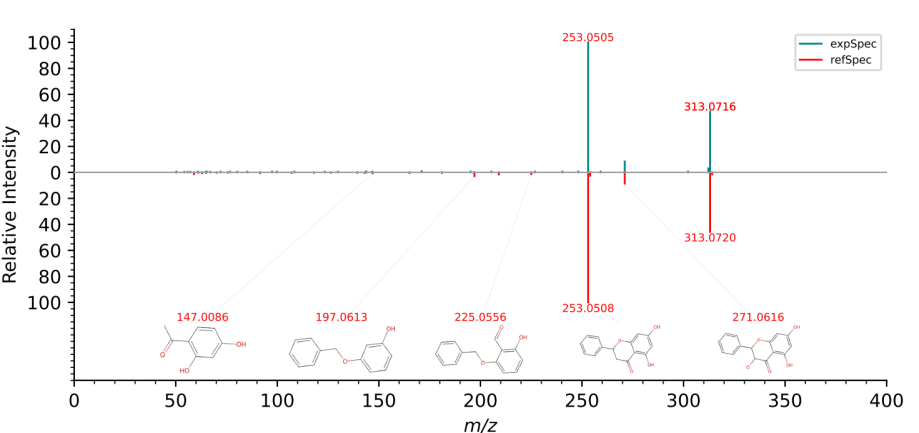


**Figure S33.** The MS/MS spectrum of Pinobanksin 3-acetate (compound 40) and its potential fragmentation pathway


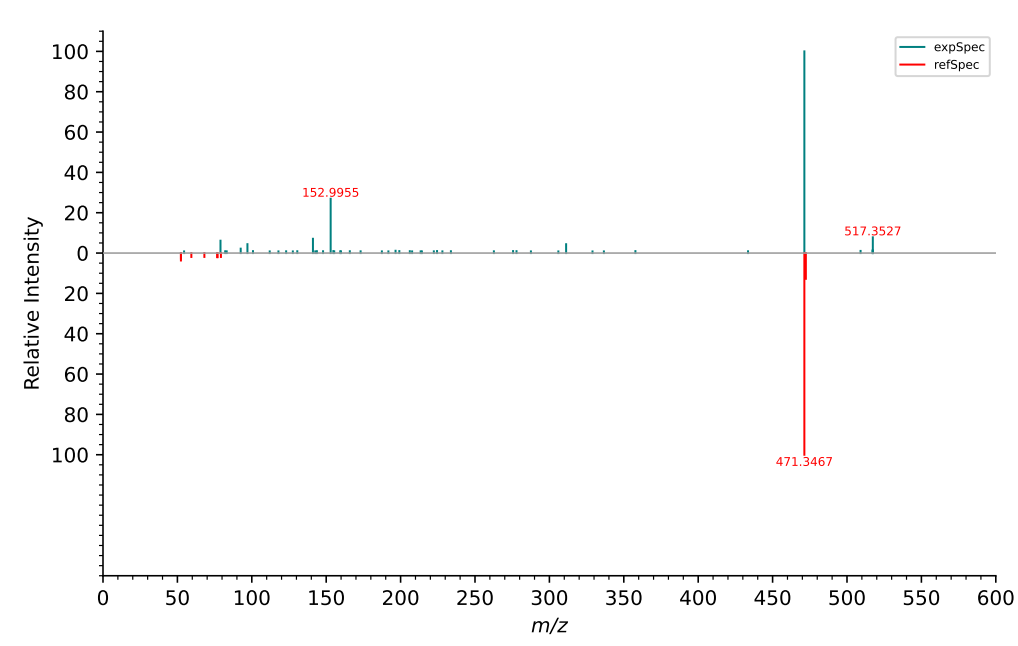


**Figure S34.** The MS/MS spectrum of Hederagenin (compound 41) and its potential fragmentation pathway


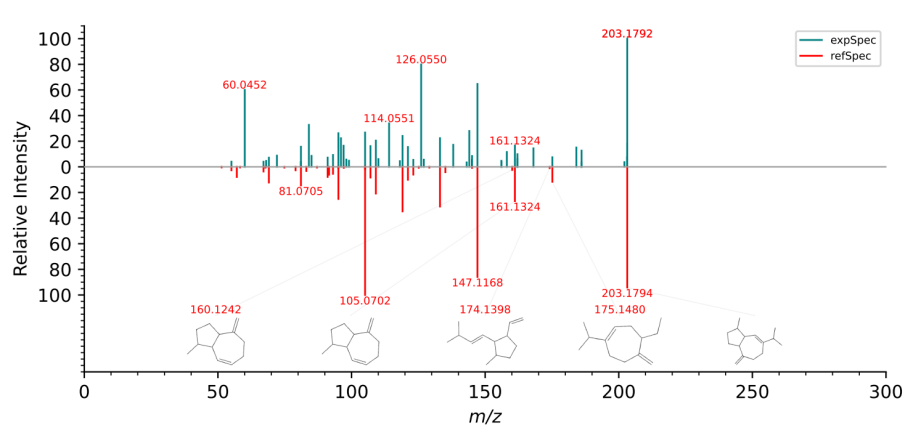


**Figure S35.** The MS/MS spectrum of Alismol (compound 42) and its potential fragmentation pathway


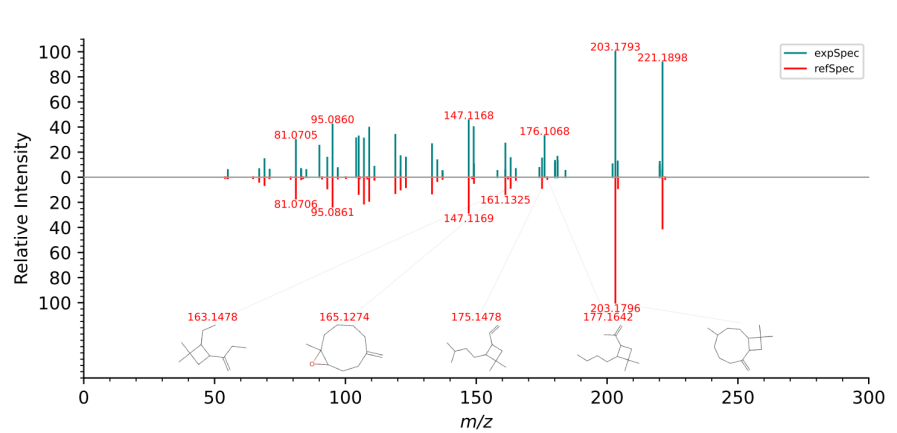


**Figure S36.** The MS/MS spectrum of Caryophyllene oxide (compound 43) and its potential fragmentation pathway


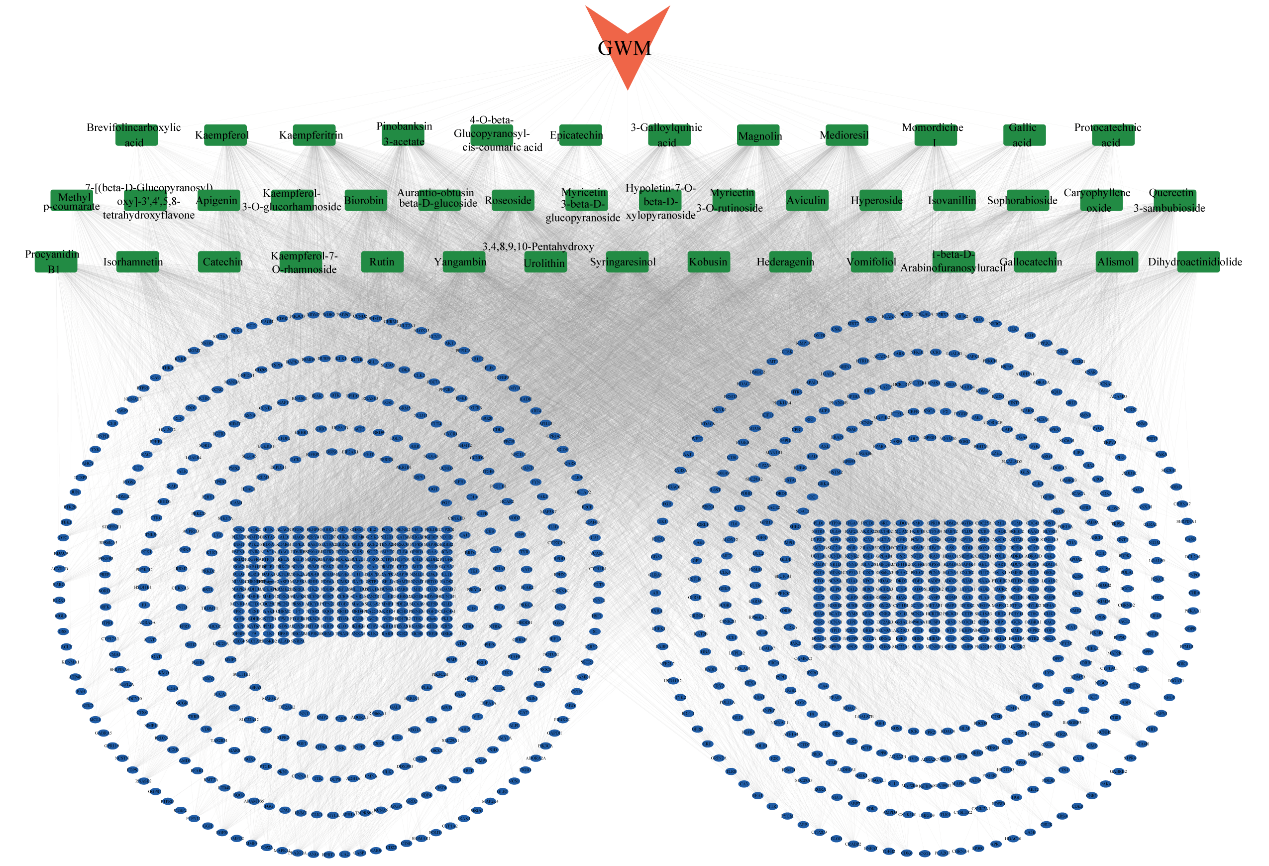


**Figure S37.** The GWM-ingredient-target interaction network (orange V-shape represents the drug GWM, green round rectangles represent ingredients, blue ellipses indicate potential drug targets, and gray lines depict the possible relationships between the drug, ingredients, and targets)


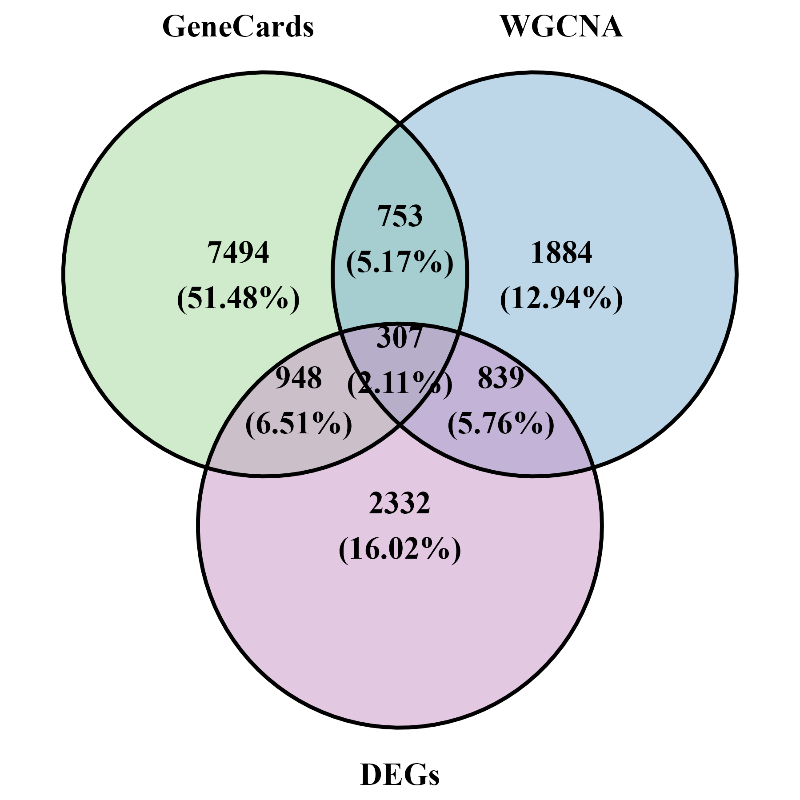


**Figure S38.** Venn diagram analysis of disease targets in asthma based on GeneCards, DEGs and WGCNA


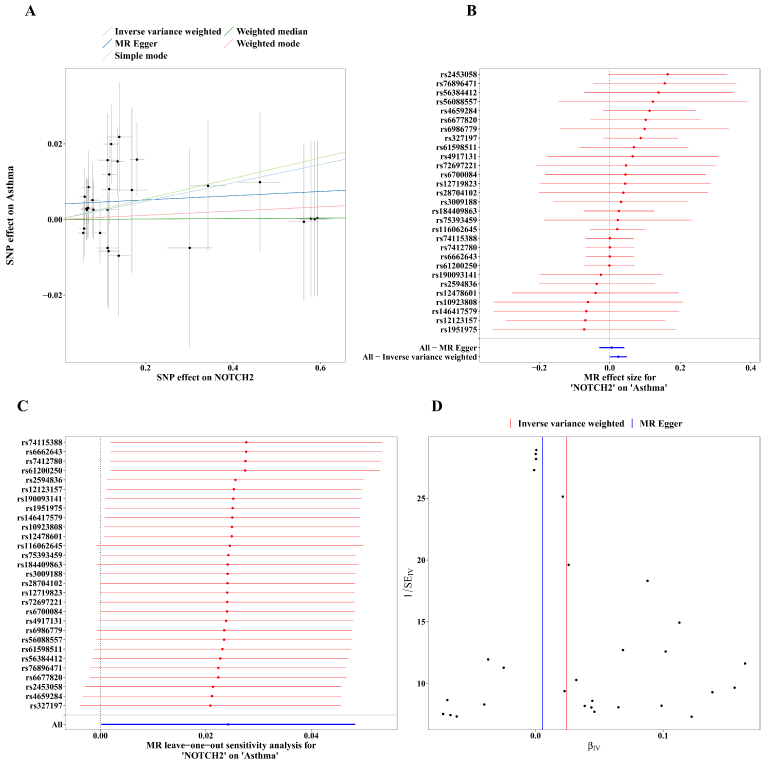


**Figure S39.** Mendelian Randomization analysis of the impact of NOTCH2 on asthma. (A) Scatter plot of genetic associations between SNPs and asthma risk. (B) Forest plot of individual SNP effects on asthma risk. (C) Leave-one-out sensitivity analysis for NOTCH2 on asthma risk. (D) Funnel plot for detecting potential horizontal pleiotropy in MR analysis.


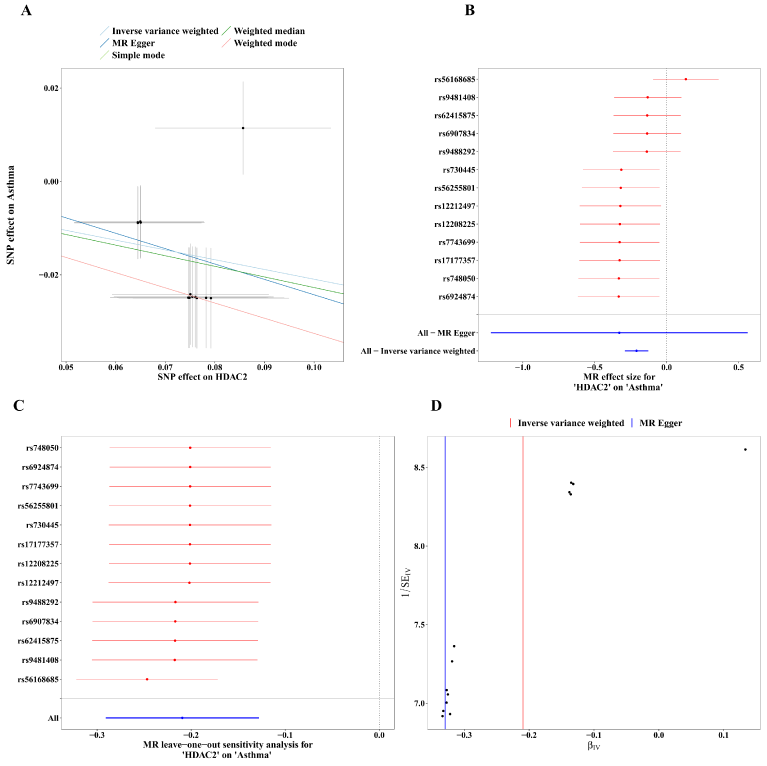


**Figure S40.**Mendelian Randomization analysis of the impact of HDAC2 on asthma. (A) Scatter plot of genetic associations between SNPs and asthma risk. (B) Forest plot of individual SNP effects on asthma risk. (C) Leave-one-out sensitivity analysis for HDAC2 on asthma risk. (D) Funnel plot for detecting potential horizontal pleiotropy in MR analysis.


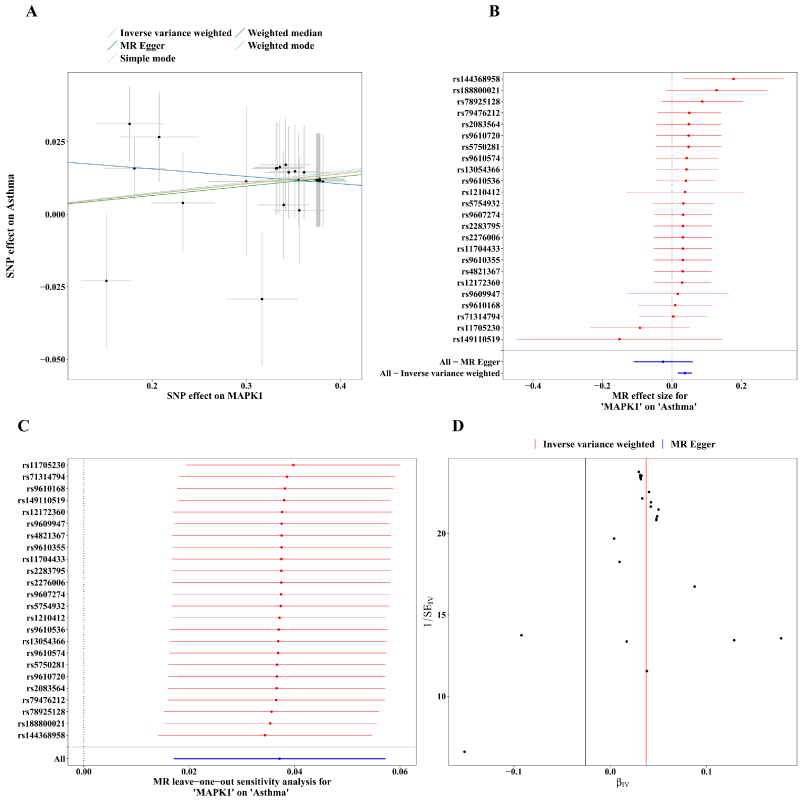


**Figure S41.** Mendelian Randomization analysis of the impact of MAPK1 on asthma. (A) Scatter plot of genetic associations between SNPs and asthma risk. (B) Forest plot of individual SNP effects on asthma risk. (C) Leave-one-out sensitivity analysis for MAPK1 on asthma risk. (D) Funnel plot for detecting potential horizontal pleiotropy in MR analysis.


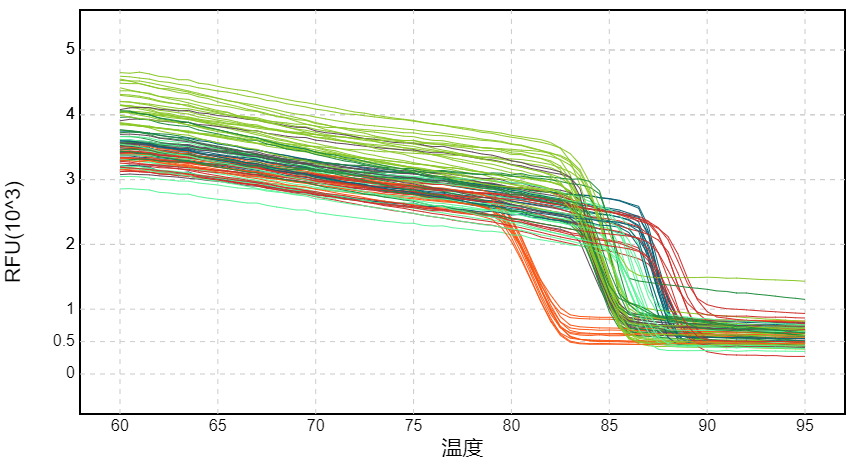


**Figure S42.** Melt curve analysis of primers: MAPK1 (#604857), HDAC2 (#f95715), NOTCH2 (#076176), and β-actin (#8bc827)


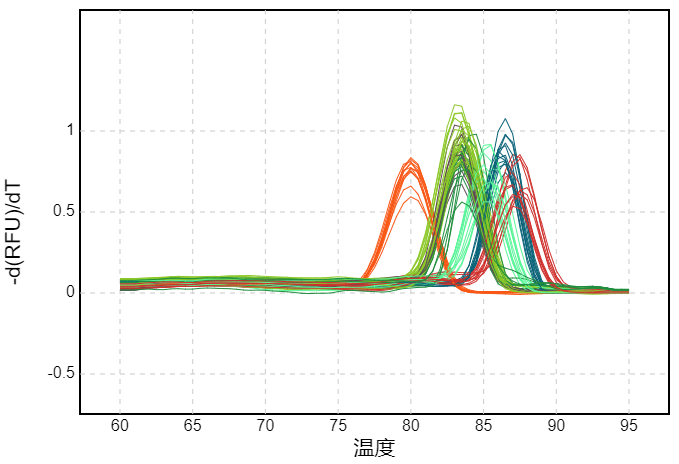


**Figure S43.** Amplification curve analysis of primers: MAPK1 (#604857), HDAC2 (#f95715), NOTCH2 (#076176), and β-actin (#8bc827)


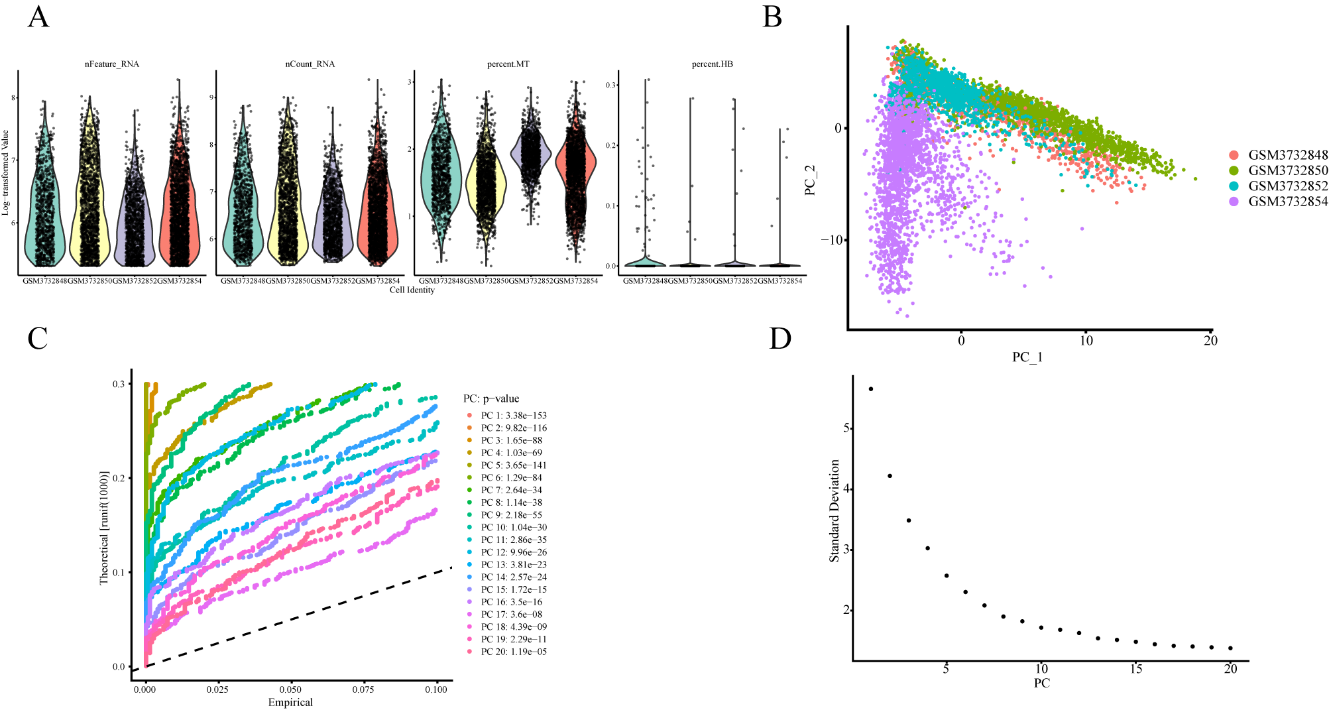


**Figure S44.** scRNA-seq data processing. (A) Quality metrics visualization of filtered cells showing the distribution of UMI counts, number of genes detected, and percentage of mitochondrial and red blood cell gene expression across samples. (B) PCA plot demonstrating the distribution of cells across different samples. (C) Statistical significance (p-values) of principal components 1-20 calculated using the JackStraw function, demonstrating the significance level of each PC. (D) Standard deviation distribution of principal components 1-20 generated by ElbowPlot function, showing the variance explained by each PC.


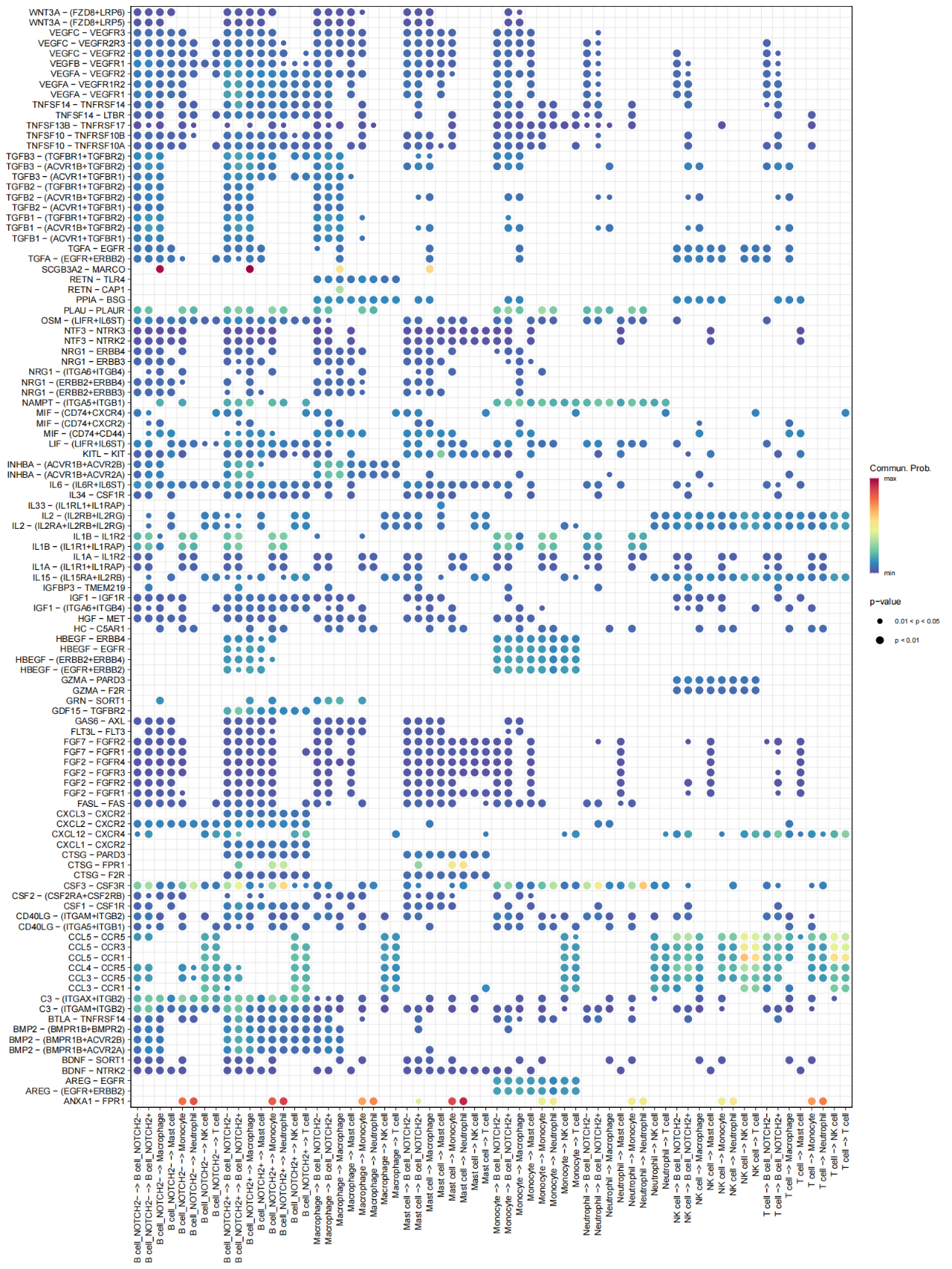


**Figure S45.** Ligand-receptor interactions of NOTCH2^+^ and NOTCH2^-^ B cells with other cell types.


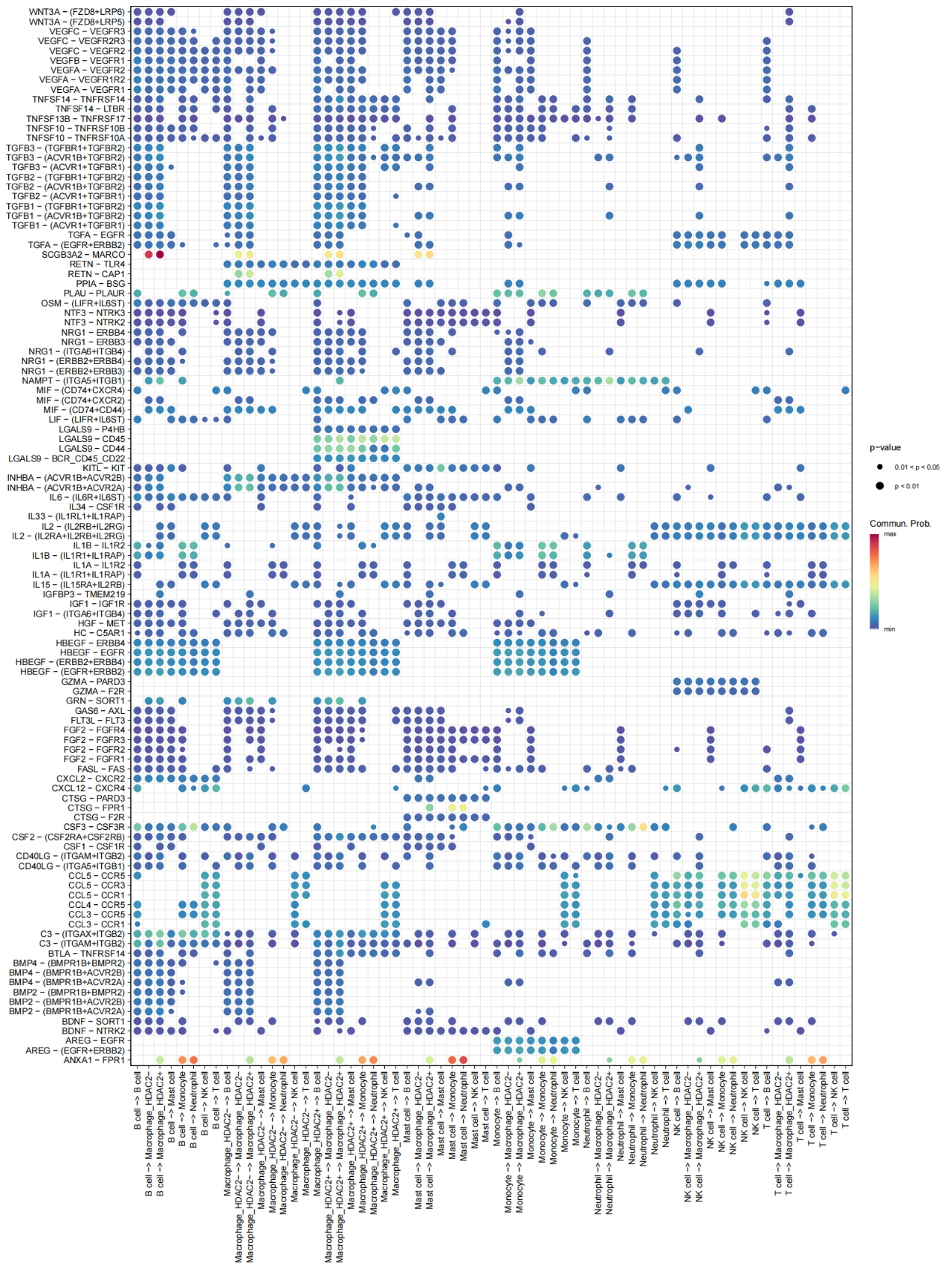


**Figure S46.** Ligand-receptor interactions of HDAC2^+^ and HDAC2^-^ macrophages with other cell types.


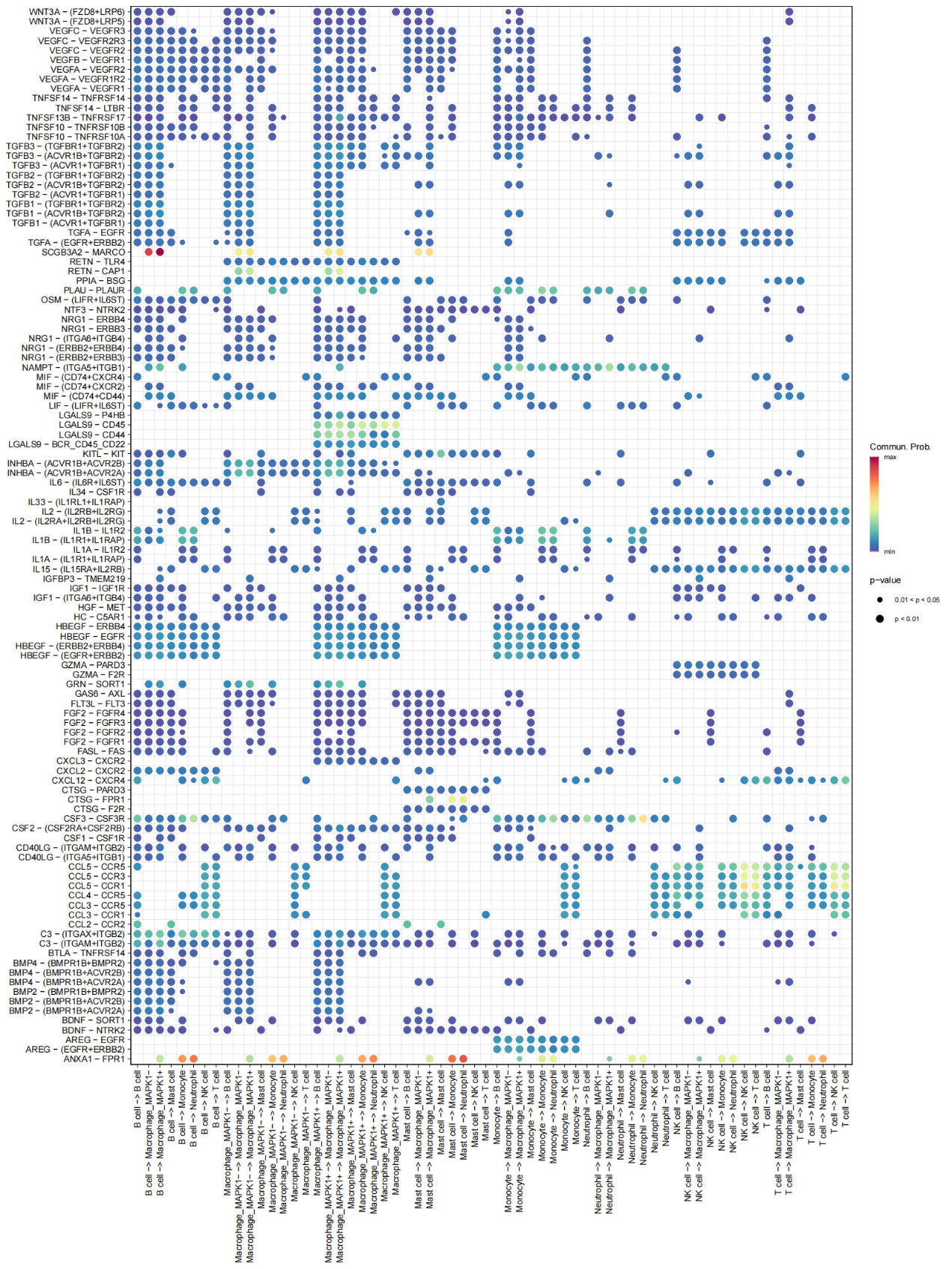


**Figure S47.** Ligand-receptor interactions of MAPK1^+^ and MAPK1^-^ macrophages with other cell types.


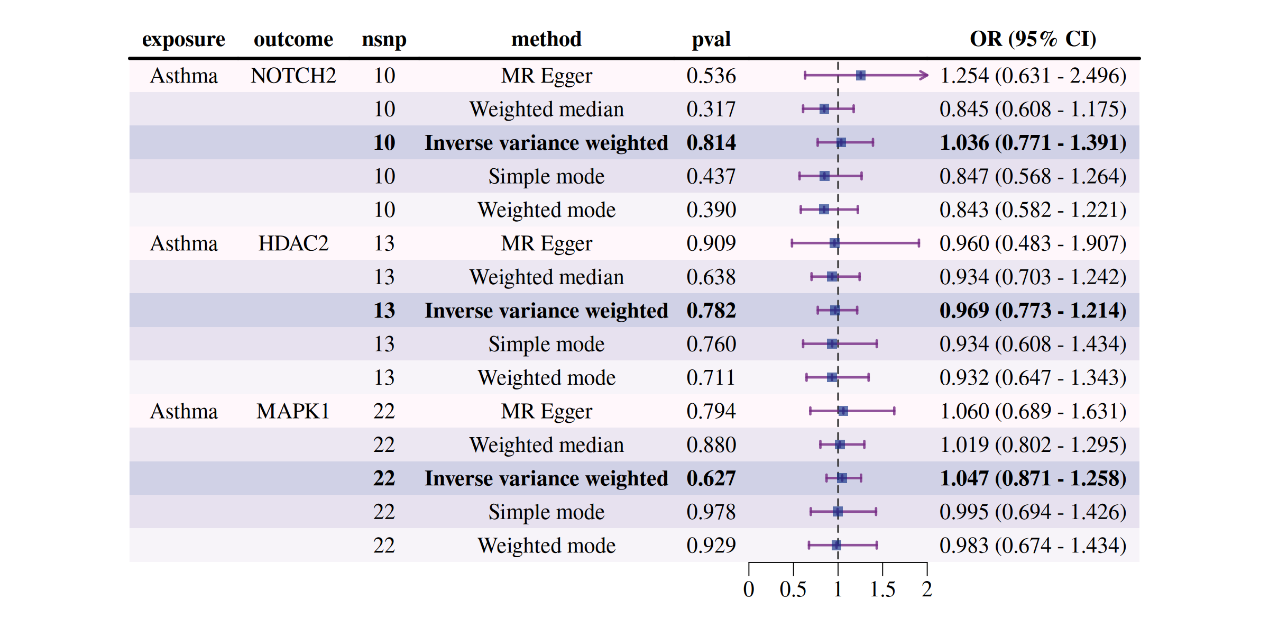


**Figure S48.** Forest plot demonstrating the causal effects of asthma on hub genes.


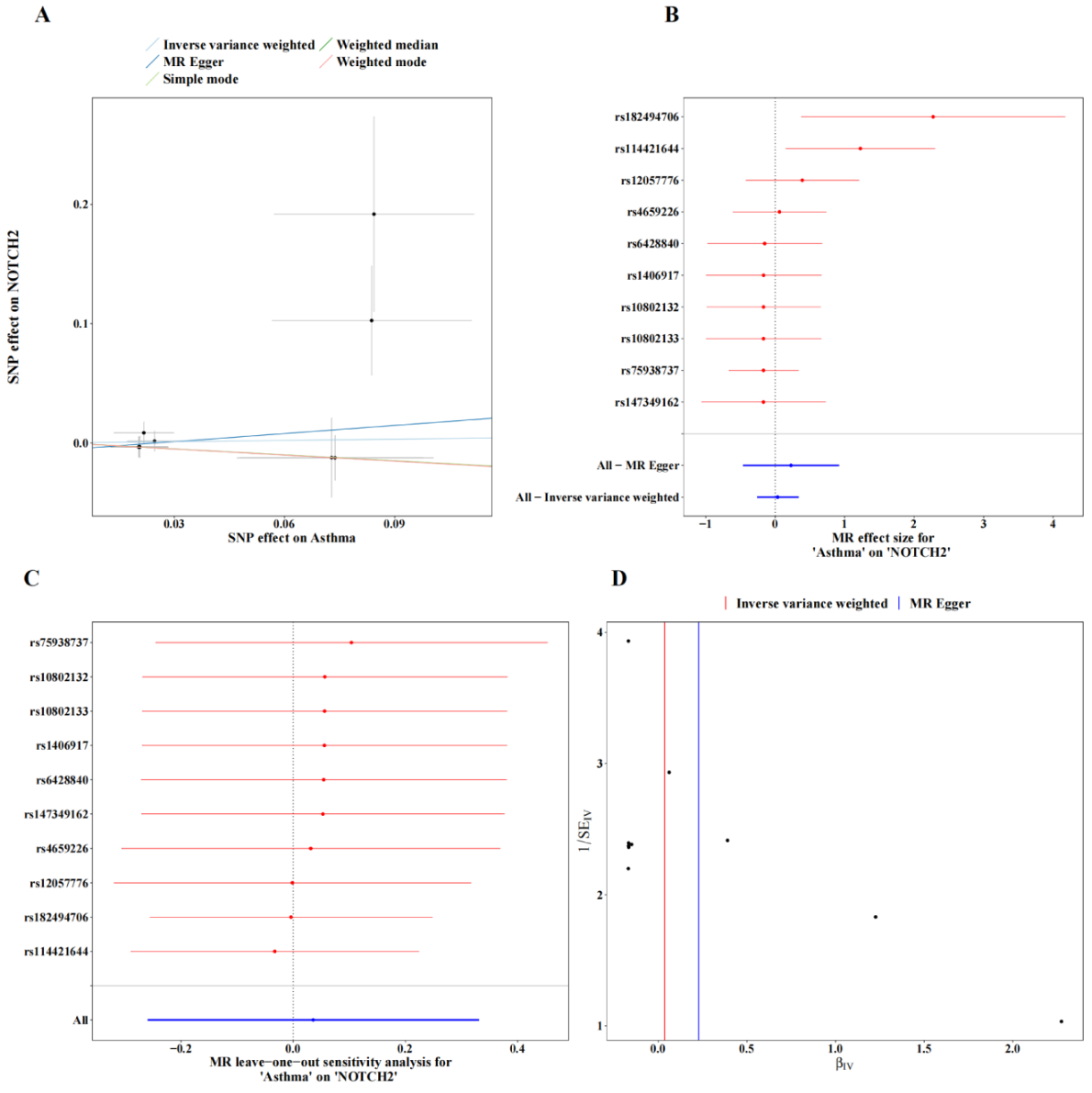


**Figure S49.** Mendelian Randomization analysis of the impact of asthma on NOTCH2. (A) Scatter plot of genetic associations between SNPs and asthma risk. (B) Forest plot of individual SNP effects on asthma risk. (C) Leave-one-out sensitivity analysis for asthma on NOTCH2. (D) Funnel plot for detecting potential horizontal pleiotropy in MR analysis.


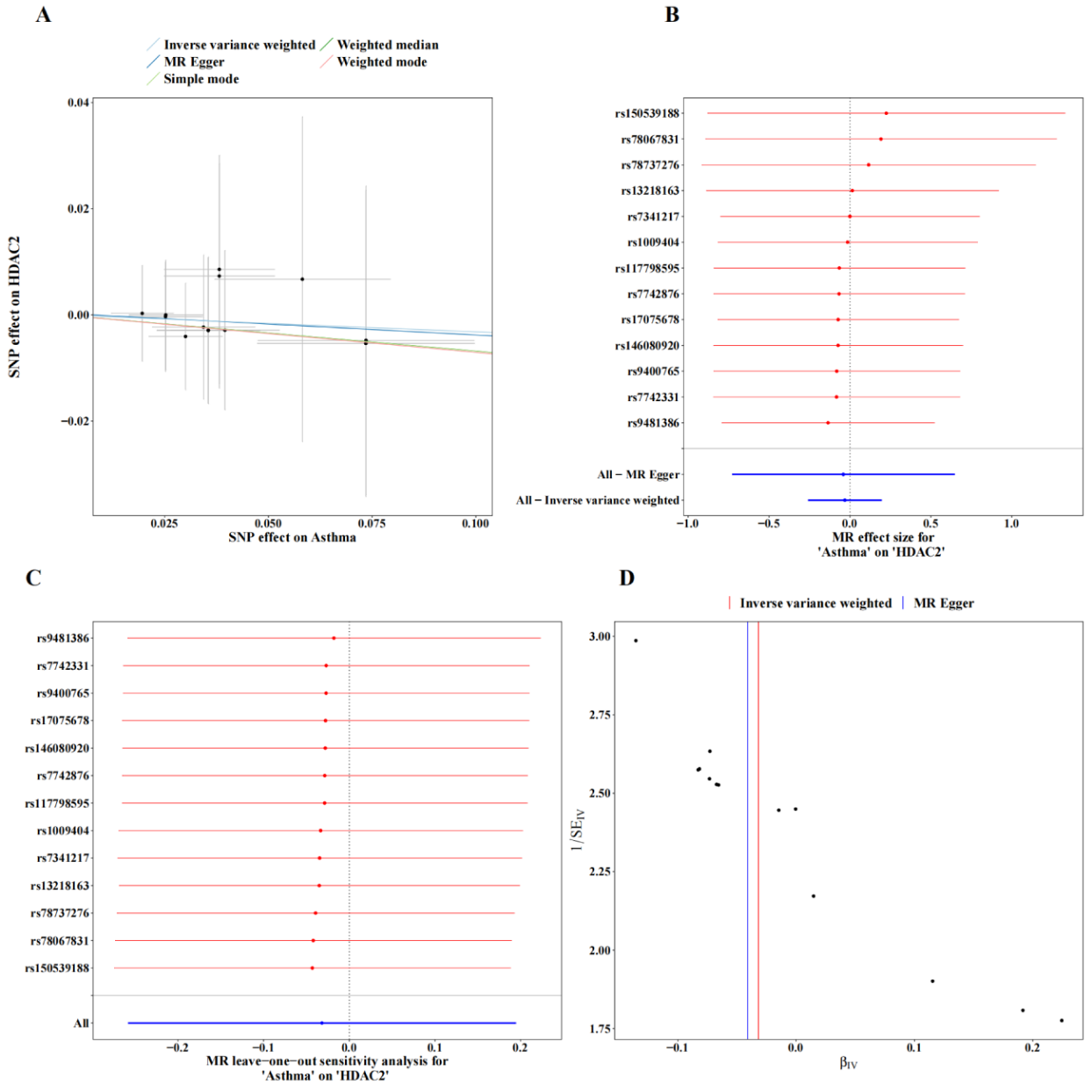


**Figure S50.** Mendelian Randomization analysis of the impact of asthma on HDAC2. (A) Scatter plot of genetic associations between SNPs and asthma risk. (B) Forest plot of individual SNP effects on asthma risk. (C) Leave-one-out sensitivity analysis for asthma on HDAC2. (D) Funnel plot for detecting potential horizontal pleiotropy in MR analysis.


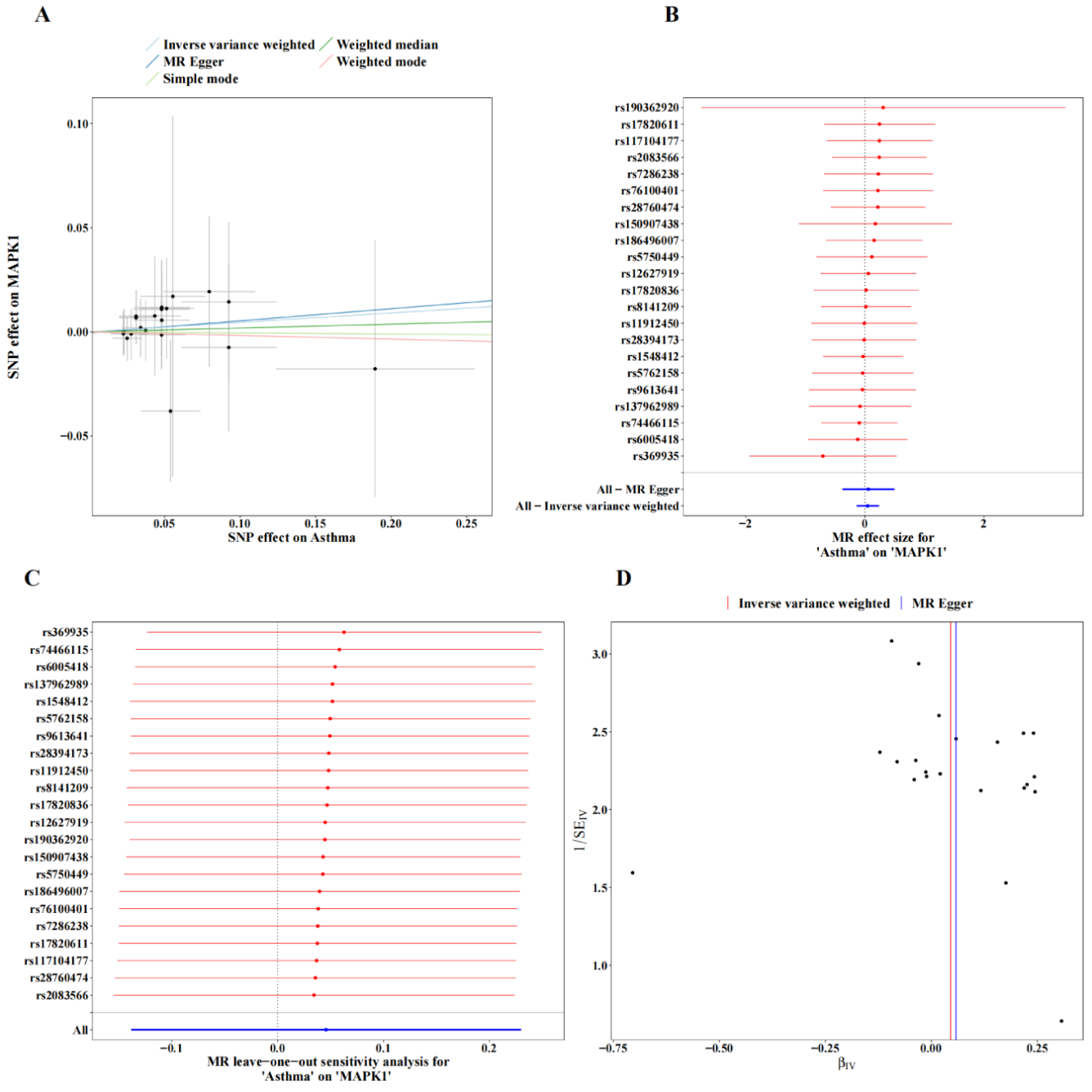


**Figure S51.** Mendelian Randomization analysis of the impact of asthma on MAPK1. (A) Scatter plot of genetic associations between SNPs and asthma risk. (B) Forest plot of individual SNP effects on asthma risk. (C) Leave-one-out sensitivity analysis for asthma on MAPK1. (D) Funnel plot for detecting potential horizontal pleiotropy in MR analysis.


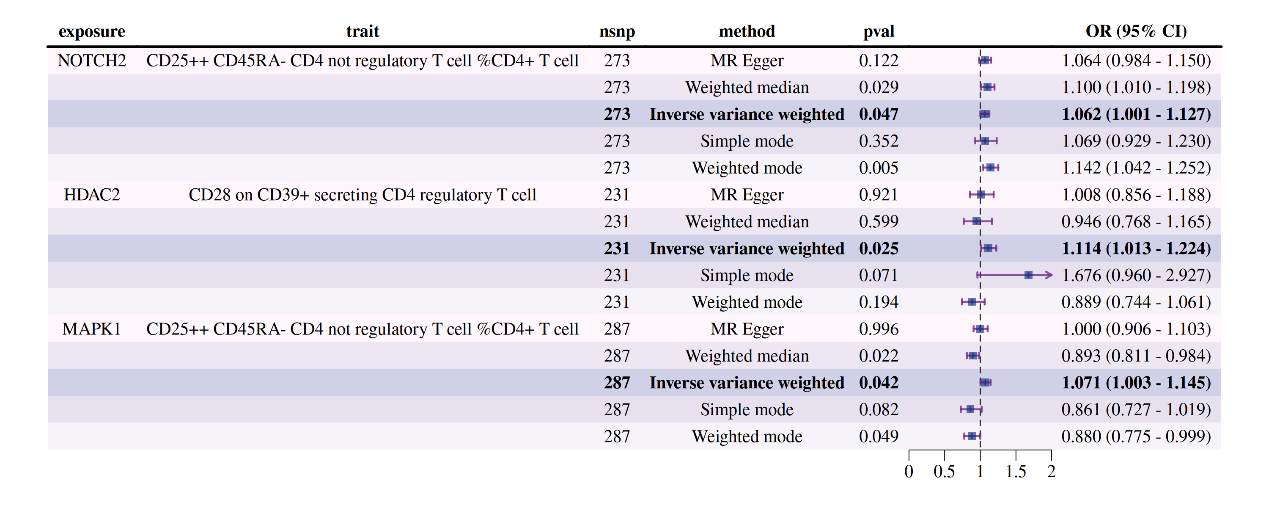


**Figure S52.** Forest plot demonstrating the causal effects of hub genes on immune cells.


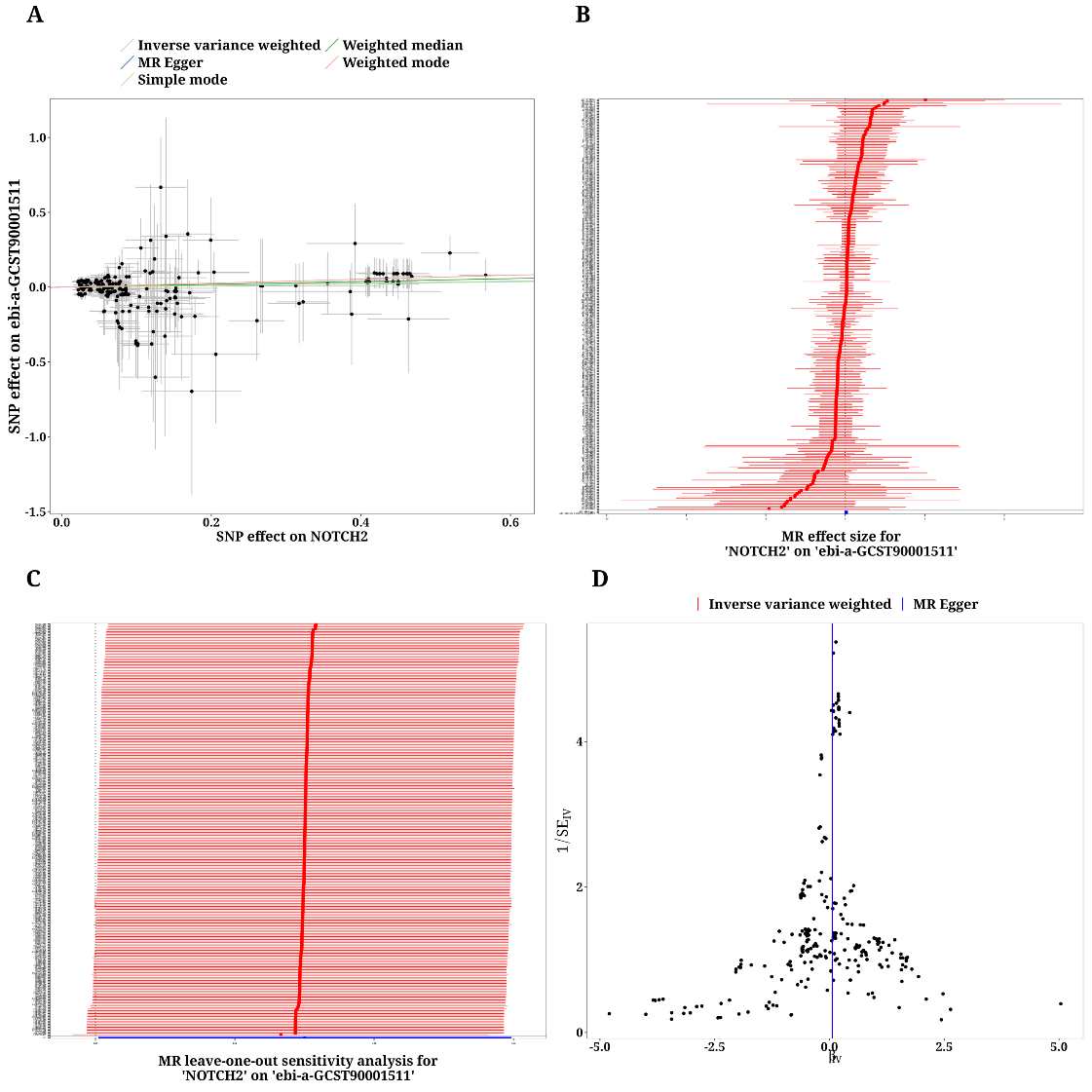


**Figure S53.** Mendelian Randomization analysis of the impact of NOTCH2 on CD25^++^ CD45RA^-^ CD4 not regulatory T cell %CD4^+^ T cell. (A) Scatter plot of genetic associations between SNPs and CD25^++^ CD45RA^-^ CD4 not regulatory T cell %CD4^+^ T cell. (B) Forest plot of individual SNP effects on CD25^++^ CD45RA^-^ CD4 not regulatory T cell %CD4^+^ T cell. (C) Leave-one-out sensitivity analysis for NOTCH2 on CD25^++^ CD45RA^-^ CD4 not regulatory T cell %CD4^+^ T cell. (D) Funnel plot for detecting potential horizontal pleiotropy in MR analysis.


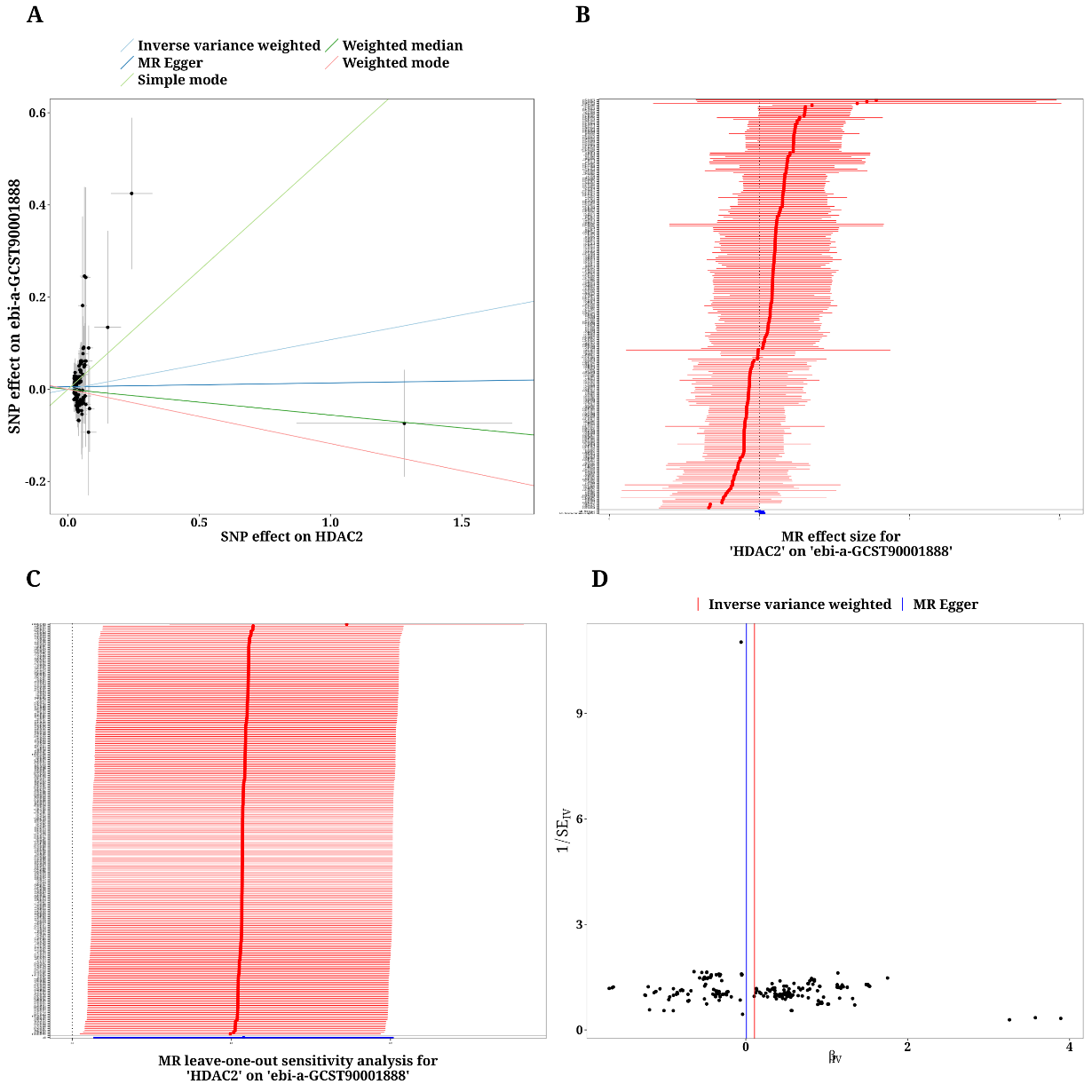


**Figure S54.** Mendelian Randomization analysis of the impact of HDAC2 on CD28 on CD39^+^ secreting CD4 regulatory T cell. (A) Scatter plot of genetic associations between SNPs and CD28 on CD39^+^ secreting CD4 regulatory T cell. (B) Forest plot of individual SNP effects on CD28 on CD39^+^ secreting CD4 regulatory T cell. (C) Leave-one-out sensitivity analysis for HDAC2 on CD28 on CD39^+^ secreting CD4 regulatory T cell. (D) Funnel plot for detecting potential horizontal pleiotropy in MR analysis.


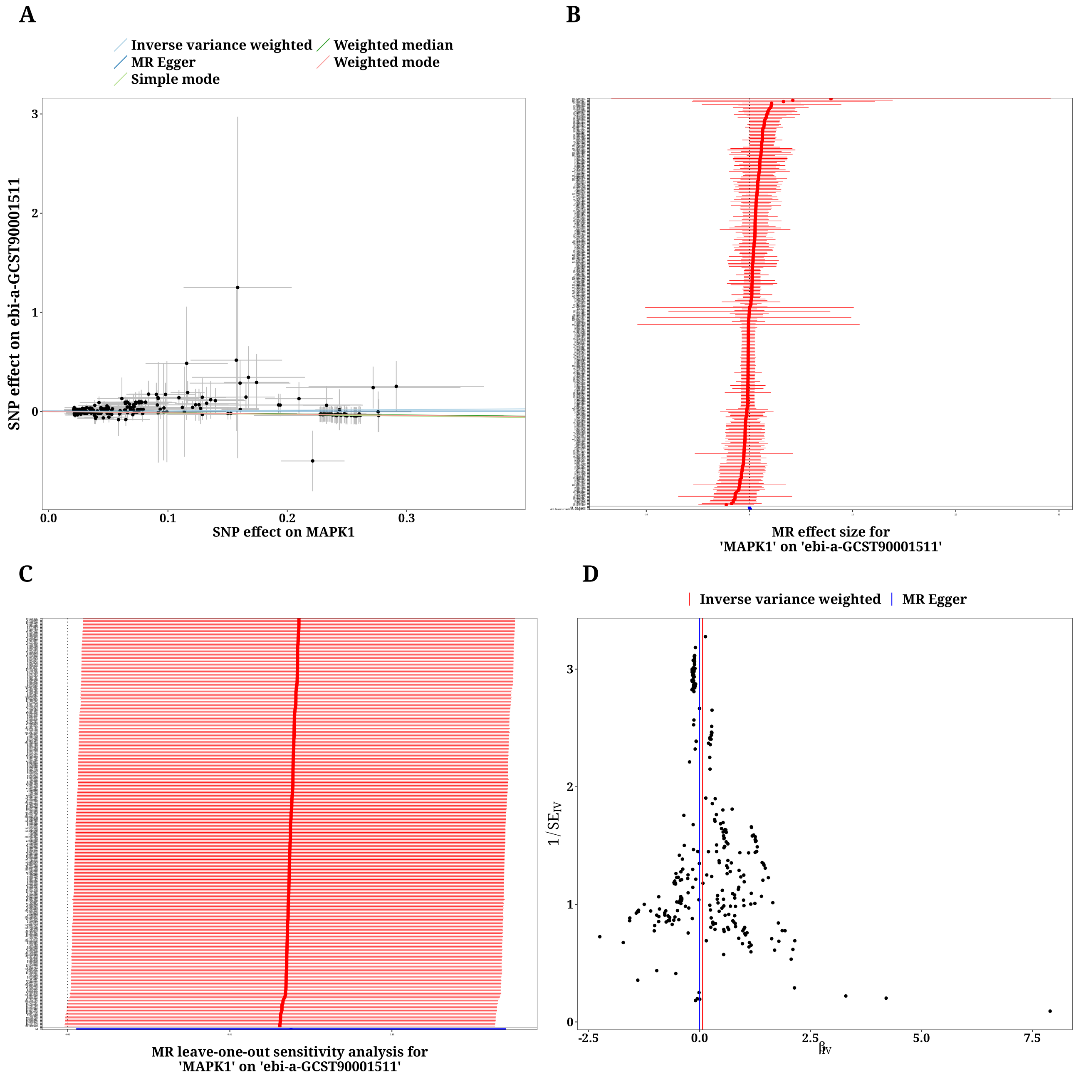


**Figure S55.** Mendelian Randomization analysis of the impact of MAPK1 on CD25^++^ CD45RA^-^ CD4 not regulatory T cell %CD4^+^ T cell. (A) Scatter plot of genetic associations between SNPs and CD25^++^ CD45RA^-^ CD4 not regulatory T cell %CD4^+^ T cell. (B) Forest plot of individual SNP effects on CD25^++^ CD45RA^-^ CD4 not regulatory T cell %CD4^+^ T cell. (C) Leave-one-out sensitivity analysis for MAPK1 on CD25^++^ CD45RA^-^ CD4 not regulatory T cell %CD4^+^ T cell. (D) Funnel plot for detecting potential horizontal pleiotropy in MR analysis.


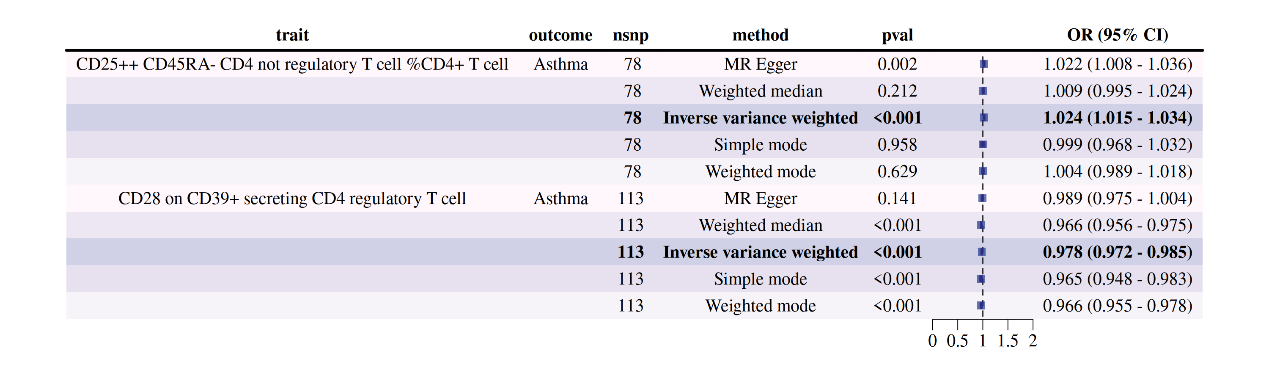


**Figure S56.** Forest plot demonstrating the causal effects of immune cells on asthma risk.


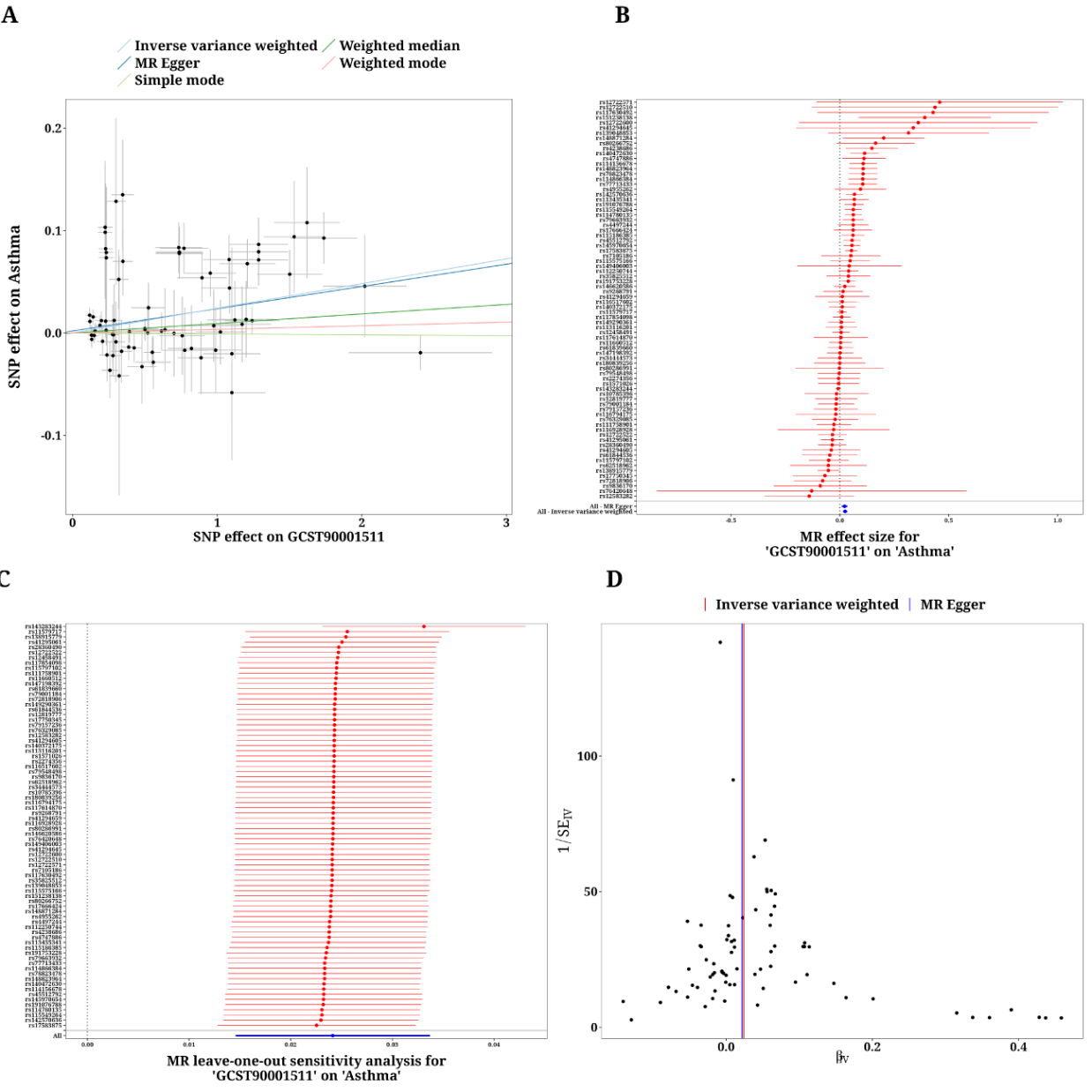


**Figure S57.** Mendelian Randomization analysis of the impact of CD25^++^ CD45RA^-^ CD4 not regulatory T cell %CD4^+^ T cell on asthma risk. (A) Scatter plot of genetic associations between SNPs and asthma risk. (B) Forest plot of individual SNP effects on asthma risk. (C) Leave-one-out sensitivity analysis for CD25^++^ CD45RA^-^ CD4 not regulatory T cell %CD4^+^ T cell on asthma risk. (D) Funnel plot for detecting potential horizontal pleiotropy in MR analysis.


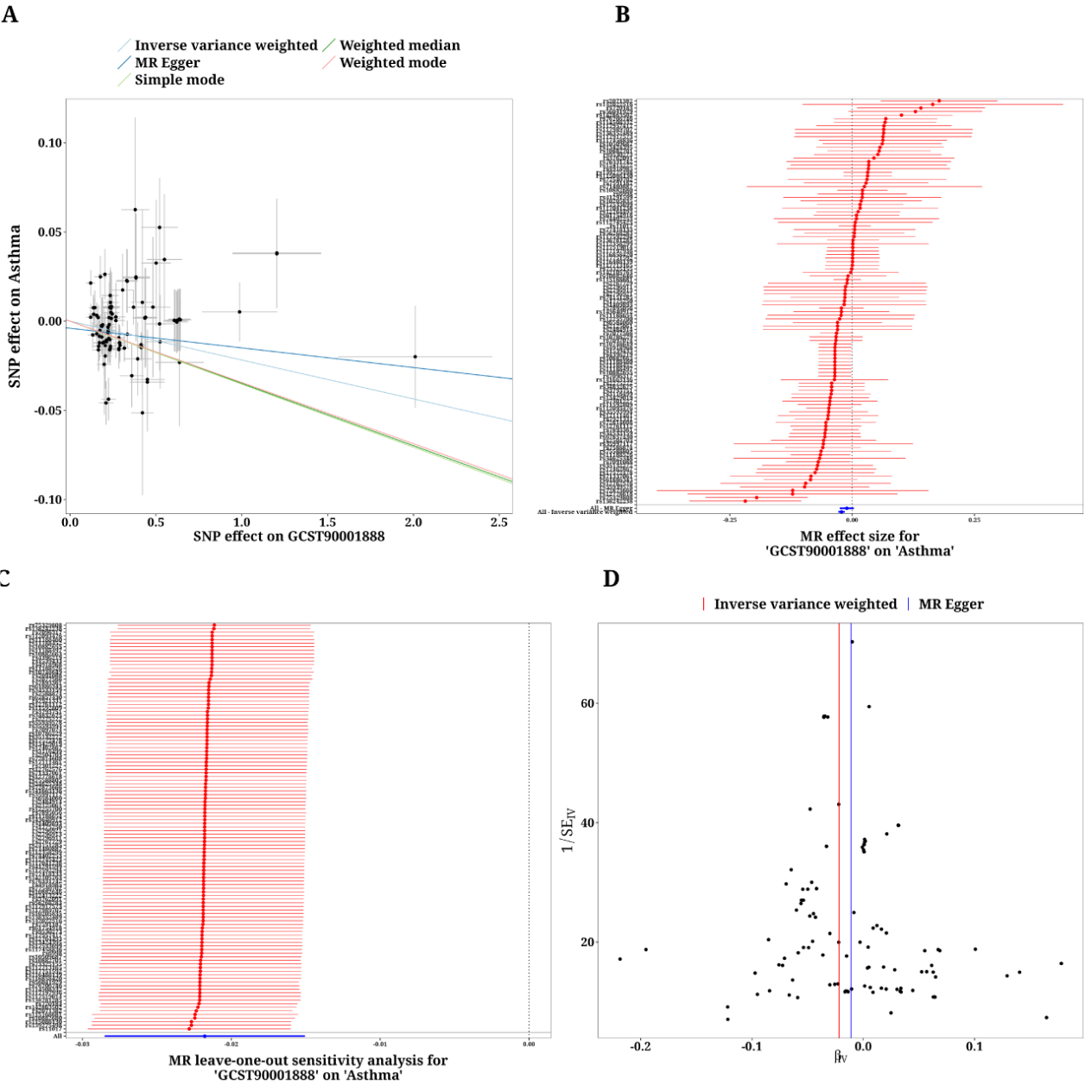


**Figure S58.** Mendelian Randomization analysis of the impact of CD28 on CD39^+^ secreting CD4 regulatory T cell on asthma risk. (A) Scatter plot of genetic associations between SNPs and asthma risk. (B) Forest plot of individual SNP effects on asthma risk. (C) Leave-one-out sensitivity analysis for CD28 on CD39^+^ secreting CD4 regulatory T cell on asthma risk. (D) Funnel plot for detecting potential horizontal pleiotropy in MR analysis.
